# Analysis of circulating microRNAs in patients with repaired Tetralogy of Fallot with and without heart failure

**DOI:** 10.1186/s12967-017-1255-z

**Published:** 2017-07-10

**Authors:** Masood Abu-Halima, Eckart Meese, Andreas Keller, Hashim Abdul-Khaliq, Tanja Rädle-Hurst

**Affiliations:** 1 Department of Human Genetics, Saarland University, 66421 Homburg/Saar, Germany; 2 Chair for Clinical Bioinformatics, Saarland University, 66041 Saarbruecken, Germany; 3 Department of Pediatric Cardiology, Saarland University Medical Center, 66421 Homburg/Saar, Germany; 4 Department of Human Genetics, Saarland University Medical Center, Kirrberger Straße 100, 66421 Homburg/Saar, Germany

**Keywords:** MicroRNA, Heart failure, Congenital heart defects, Tetralogy of Fallot

## Abstract

**Background:**

MicroRNAs (miRNAs) are a class of regulatory RNAs that regulate gene expression post-transcriptionally. Little, however, is known on the expression profile of circulating miRNAs in Tetralogy of Fallot (TOF) patients late after surgical repair. In this study, we aimed to identify the specific patterns of circulating miRNAs in blood of patients with repaired, non-syndromic TOF and to assess whether these specific miRNAs may be useful to differentiate patients with and without heart failure.

**Methods:**

SurePrint™ 8 × 60 K Human v16 miRNA arrays were used to determine miRNA expression profiles in 15 healthy controls and 37 patients after TOF repair of whom 3 had symptomatic right heart failure. The expression levels of selected miRNAs have been validated by quantitative reverse transcription polymerase chain reaction (RT-qPCR). Enrichment analyses of altered miRNA expression were predicted using bioinformatic tools.

**Results:**

Compared with healthy controls, a total of 49, 58 and 77 miRNAs were found to be significantly altered in TOF patients (TOF-all), TOF patients with (TOF-HF) and without symptomatic right heart failure (TOF-noHF) (>2.0-fold change, adjusted *P* < 0.05), respectively. Three miRNAs namely miR-181d-5p, miR-206 and miR-625-5p were validated by RT-qPCR in all TOF groups. The area under the receiver operating characteristic curve (AUC) for miR-181d-5p, miR-206 and miR-625-5p were 0.987, 0.993 and 0.769 in TOF-all and 0.990, 0.994 and 0.749 in TOF-noHF, respectively. Moreover, expression levels of miR-625-5p, miR-1233-3p and miR-421 were lower in TOF-HF compared to TOF-noHF (*P* = 0.012).

**Conclusions:**

Altered expression levels of circulating miRNAs were found in TOF patients late after surgical repair and are different to those seen in the right ventricular myocardium of infants with TOF. Expression levels of miR-421, miR-1233-3p and miR-625-5p are lower in TOF patients with symptomatic right heart failure and thus may indicate disease progression in these patients.

**Electronic supplementary material:**

The online version of this article (doi:10.1186/s12967-017-1255-z) contains supplementary material, which is available to authorized users.

## Background

Tetralogy of Fallot (TOF) is the most frequent cyanotic congenital heart defect with a prevalence of 5–7% per 10,000 live births [1]. It is characterized by varying degrees of right ventricular outflow tract (RVOT) obstruction and a large ventricular septal defect requiring surgical repair within the first years of life. After surgical repair, residual lesions such as restenosis of the RVOT or pulmonary regurgitation as well as right or left ventricular dysfunction may occur during long-term follow-up [2–6]. MicroRNAs (miRNAs) are a class of non-coding RNAs of approximately 22 nucleotides in length that regulate gene expression post-transcriptionally via sequence-specific interaction with the 3′ UTR of target mRNAs, resulting in inhibition of translation and/or mRNA degradation [7]. Studies have shown that dysfunctions of miRNAs are associated with congenital heart disease (CHD) [8], including TOF [9–12]. These studies have highlighted the alteration of miRNA profile in the myocardial tissue. Recently, alterations in miRNA expression levels in ROVT myocardial tissue of infants with TOF have been identified [9, 10]. Specifically, O’Brien et al. [10] identified 61 miRNAs with significantly altered expression levels and Bittel et al. [9] found that miR-421 exhibited the most significant expressed miRNA in the RVOT myocardial tissue of infants with TOF. Another study by Liang et al. [13] explored the role of miRNA in TOF patients and identified 75 miRNAs with altered expression levels. Among the altered miRNAs, miR-940 was the most down-regulated one [13]. Apart from their importance in myocardial tissue, miRNAs have also been detected in blood of patients with various cardiovascular diseases, offering the possibility to use them as potential biomarkers [14–18]. In adult patients with dyspnea, circulating miR-423-5p has been identified as a potential biomarker that can distinguish patients with left-sided heart failure from those with other causes of dyspnea [19]. However, this miRNA failed as a biomarker in patients after atrial switch operation for transposition of the great arteries [20]. In addition, Lai et al. identified 23 miRNAs in patients late after atrial switch operation for complete transposition of the great arteries (TGA), of which miR-18a and miR-486-5p were negatively related to the systolic function of the systemic right ventricle [16]. In TOF patients, however, investigations of circulating miRNAs are still lacking. With this study, we aim to identify the specific patterns of circulating miRNAs in blood of patients with repaired, non-syndromic TOF and to assess whether these specific miRNAs may be useful to differentiate patients with and without heart failure.

## Methods

### Patients and blood collection

A total of 37 consecutive patients late after surgical repair of non-syndromic TOF visiting our outpatient clinic specialized in the treatment of adults with congenital heart disease were enrolled in the study and compared to 15 age-matched healthy controls. In all healthy controls, a physical examination including measurement of blood pressure and transcutaneous oxygen saturation at rest as well as two-dimensional echocardiography were performed to verify the absence of any heart abnormality. Mean age of TOF patients and controls was 30.2 ± 10.8 years. In all TOF patients, two- and three-dimensional echocardiography was performed using a Vivid™ E9 Ultrasound System (GE Healthcare, Horten, Norway) to assess right and left ventricular dimensions as well as systolic biventricular function. In all controls and patients, venous blood from the cubital vein was drawn and 2.5 ml collected into PAXgene™ blood tubes (Becton–Dickinson, Heidelberg, Germany) shortly after echocardiographic evaluation. In the patient group, venous blood samples were drawn to determine the routine laboratory parameters as well as the concentrations of NT-proBNP and high sensitive troponin T (hsTNT) that were measured using electrochemiluminescence sandwich immunoassays namely Cobas^®^ proBNP II and Elecsys^®^ Troponin T high sensitive by Roche Diagnostics, Basel, Switzerland. All PAXgene blood tubes were stored at room temperature for 24 h to ensure complete lysis of the blood cells, then stored at −20 °C for several days and finally transferred to −80 °C for long-term storage until RNA isolation. Subjects were recruited and evaluated in accordance with relevant guidelines and regulations following the approval of the local ethics committee (Ethical vote No. 73/09). All participants or their legal guardians gave written informed consent before enrolment.

### RNA isolation and quality assessment

Total RNA including miRNAs from venous blood collected into PAXgene tubes was isolated using PAXgene Blood miRNA Kit on the QIAcube™ robot (Qiagen, Hilden, Germany) following the manufacturer’s recommendations and included DNase I treatment (Qiagen, Hilden, Germany). To confirm the absence of genomic DNA contamination, a conventional PCR with exon spanning primers for GAPDH was performed [21]. The concentration of isolated total RNA, including miRNAs was measured using NanoDrop ND-2000 spectrophotometer (Thermo Fisher Scientific, Massachusetts, USA). RNA purity was estimated by examining the OD 260/280 and the OD 260/230 ratios. The RNA integrity was assessed by Agilent 2100 Bioanalyzer using the RNA Nano 6000 Kit according to the manufacturer’s instructions (Agilent Technologies, California, USA).

### Analysis of circulating miRNAs by microarray

MiRNA expression analysis was performed according to the manufacturer’s instructions using SurePrint™ 8 × 60 K Human v16 miRNA microarrays (Agilent Technologies, California, USA). These microarrays contain ~40 replicates for each probe complement to each of the 1205 mature miRNAs of miRBase v16. These probes act in concert to measure the miRNA of interest, and the data are combined later during software analysis. All probes are randomly distributed on the array, and cross hybridization is prevented by the addition of a G residue and a hairpin at the 50 end of the probe. Microarray hybridizations were done following the manufacturer’s recommendations. In brief, a total of 100 ng total RNA from each sample was dephosphorylated by incubation with calf intestinal phosphatase at 37 °C for 30 min and denatured with the use of 100% dimethyl sulfoxide at 100 °C for 7 min. Samples were labeled with pCp-Cy3 with the use of T4 ligase at 16 °C incubation for 2 h. Each labeled RNA sample was hybridized onto an individual sub-array of the 8 × 60 K format Agilent miRNA microarray slide, with each array containing probes for 1205 human miRNAs according to miRBase v16. Then the microarrays were loaded and incubated at 55 °C for 20 h with rotation. After two washing steps, the arrays were dried and scanned using the Agilent Microarray Scanner at 3 microns in double path mode. Data was acquired using Agilent AGW Feature Extraction software version 10.10.11 (Agilent Technologies, California, USA).

### Analysis of circulating miRNAs by RT-qPCR

RT-qPCR validation analysis was performed according to the manufacturer’s instructions using EPIK™ miRNA Select Assays (Bioline, London, UK) to validate the results obtained from miRNA microarray initial survey experiments. In brief, 100 ng RNA was converted into cDNA using the EPIK™ miRNA RT kit (Bioline, London, UK). The cDNA was diluted 1:10 and 4 µl of cDNA was mixed with 10 µl 2X SensiSMART™ PCR Master Mix, 2 µl PCR Primer for 10 miRNAs (miR-181d-5p, miR-142-5p, miR-140-3p, miR-1233-3p, miR-183-5p, miR-206, miR-339-5p, miR-421 and miR-625-5p) and U6 snRNA as an endogenous control (Bioline, London, UK) in a total volume of 20 µl. All RT-qPCR experiments were performed using the QIAgility™ automated PCR setup robot (Qiagen, Hilden, Germany) before performing RT-qPCR analysis on a StepOnePlus™ Real-Time PCR system (Applied Biosystems, Foster City, USA). The melting curve analysis was used to control the specificity of RT-qPCR products.

### Statistical analysis

For statistical analysis, the freely available R statistical environment v.2.14.2 was used to analyze the differences in miRNA expression in patient groups compared to healthy controls. Raw data generated by Agilent Feature Extraction image analysis software was quantile normalized. A significance level of miRNAs was analyzed by applying an unpaired two-tailed t test and area under the receiver operating characteristic curve (AUC) values for each miRNA were computed. The relative quantitative method of 2^−ΔΔCq^ was used to measure the dynamic change of specifically selected miRNAs [22]. In detail, first, the threshold cycle (Ct) value for each sample was determined. Next, the Delta Ct (ΔCt) value between the Ct value of the target miRNA and the Ct value of the endogenous control was calculated using equation: ΔCt = Ct (target miRNA) − Ct (endogenous control). In the next steps, the ΔΔCt value and the normalized target expression were calculated: ΔΔCt = ΔCt (sample [e.g., TOF sample]) − ΔCt (control [e.g., healthy controls]. Finally, the fold change was calculated using the equation 2^−ΔΔCt^. The analyses of clinical data and their correlation to miRNA expression levels were performed using the statistical software package SPSS (SPSS version 19; SPSS Inc., Chicago, Illinois). Data are presented as the mean ± standard deviation or medians (interquartile range) as appropriate. The effect of miRNAs on target genes and networks has been evaluated using miRTargetLink [23].

## Results

### Patient characteristics

Of the 37 patients enrolled in the study 22 were female and 15 were male. Three of them presented with peripheral edema, hepatomegaly and significant weight gain over a short period of time indicating symptomatic right heart failure. The other 34 patients were in a clinically stable condition without any clinical signs of right heart failure. Clinical characteristics of TOF patients with and without symptomatic right heart failure are displayed in Table 1. In TOF patients with symptomatic right heart failure, NYHA class, right and left ventricular volumes were significantly elevated whereas measures of systolic ventricular function such as ejection fraction or velocity time integral above the aortic valve were significantly reduced. Moreover, NT-proBNP levels were also significantly higher in these patients.

**Table 1:** Characteristics of TOF patients with (TOF-HF) and without symptomatic right heart failure (TOF-noHF)

Variables	Controls (n = 15)	All patients (n = 37)	TOF-noHF (n = 34)	TOF-HF (n = 3)	*P* value*
Age at follow-up (years)	30.2 ± 10.8	30.2 ± 10.8	29.9 ± 10.7	33.0 ± 13.9	NS
Follow-up time (years)	–	24.8 ± 8.9	25.1 ± 8.1	24.7 ± 17.7	NS
Age at primary corrective surgery (years)	–	4.9 ± 3.5	4.6 ± 3.5	8.0 ± 2.6	0.079
Prior BT shunt (%)	–	20/37 (54.1)	19/34 (55.9)	1/3 (33.3)	NS
Presence of pulmonary bioprosthetic valve or homograft (%)	–	15/37 (40.5)	14/34 (41.2)	1/3 (33.3)	NS
Presence of moderate to severe residual pulmonary regurgitation (%)	–	8/37 (21.6)	6/34 (17.6)	2/3 (66.7)	NS
Residual peak systolic pressure gradient across RVOT >40 mmHg (%)	–	11/37 (29.7)	10/34 (29.4)	1/3 (33.3)	NS
NYHA functional class	1.1 ± 0.3	1.3 ± 0.6	1.1 ± 0.3	2.7 ± 0.6	<0.001
Systolic blood pressure (mmHg)	119.7 ± 10.8	122.2 ± 13.2	122.7 ± 13.7	115.7 ± 2.5	NS
Diastolic blood pressure (mmHg)	66.7 ± 6.2	70.4 ± 8.4	70.0 ± 8.6	75.0 ± 3.6	NS
Transcutaneous oxygen saturation at rest (%)	98.1 ± 1.1	97.7 ± 1.4	97.7 ± 1.4	97.3 ± 1.5	NS
Presence of any medication such as β-blocker, ACE inhibitor/AT1 blocker or diuretics (%)	–	14/37 (37.8)	11/34 (32.2)	3/3 (100.0)	0.047
Ejection fraction of RV (%)	62.9 ± 3.7	53.5 ± 8.5	55.5 ± 5.9	33.3 ± 2.3	0.005
Enddiastolic volume of RV (ml/m^2^ BSA)	28.7 ± 8.5	61.8 ± 29.2	55.9 ± 22.3	122.4 ± 23.9	0.007
Endsystolic volume of RV (ml/m^2^ BSA)	10.6 ± 3.35	30.6 ± 21.1	25.5 ± 12.2	84.1 ± 21.4	0.006
Ejection fraction of LV (%)	62.6 ± 3.2	58.4 ± 9.9	60.6 ± 6.0	33.1 ± 11.8	0.005
Enddiastolic volume of LV (ml/m^2^ BSA)	62.5 ± 13.9	58.5 ± 17.3	56.0 ± 14.4	87.0 ± 25.0	0.014
Endsystolic volume of LV (ml/m^2^ BSA)	23.5 ± 6.0	27.2 ± 13.0	24.5 ± 8.1	57.9 ± 20.3	0.005
VTI above aortic valve (cm)	27.4 ± 3.5	22.6 ± 4.6	23.4 ± 3.8	14.0 ± 3.6	0.008
Creatinine (mg/dl)	ND	0.83 (0.69–0.90)	0.82 (0.69–0.88)	0.92 (0.70–1.36)	NS
Estimated glomerular filtration rate (ml/min)	ND	106.2 ± 19.8	107.8 ± 15.6	89.4 ± 49.6	NS
NT-proBNP (pg/ml)	ND	146.0 (74.8–236.2)	139.6 (65.6–215.2)	1747.0 (1531.0–2939.5)	0.005
High sensitive troponin T (pg/ml)	ND	4.5 (3.0–6.0)	4.0 (3.0–6.0)	6.0 (6.0–9.5)	0.057

Mean ± standard deviation or median (interquartile range) are used

*TOF* Tetralogy of Fallot, *BT* Blalock–Taussig, *RVOT* right ventricular outflow tract, *NYHA* New York Heart Association, *RV* right ventricle, *BSA* body surface area, *LV* left ventricle, *VTI* velocity time integral, *ND* not determined, *NS* not significant

* Patients with compared to those without symptomatic right heart failure (TOF-HF versus TOF-noHF)

### Differentially expressed miRNAs

Using the high-throughput SurePrint G3 Human v16 miRNA microarray platform, we measured the expression of 1205 human mature miRNAs of miRBase v16. Following background correction and quantile normalization, expression levels of circulating miRNAs were screened in a total of 21 TOF patients (TOF-all), of whom 3 had symptomatic right heart failure (TOF-HF, n = 3) and 18 had no signs of right heart failure (TOF-noHF, n = 18) as well as 15 healthy controls. Next, we performed an unpaired t test to identify those miRNAs that showed a differential expression in TOF-all compared to healthy controls as well as in TOF-noHF and TOF-HF, each also compared to an age-matched healthy control group (Additional file 1: Table S1). We next considered those miRNAs as significantly differentially expressed that showed a fold change of >2.0 between considered groups and a *P* value <0.05 in an unpaired *t* test (Table 1). In total, 49 significantly deregulated miRNAs were identified in TOF-all patients (27 up-regulated and 22 down-regulated) (Table 2a, P < 0.05, FDR adjusted), 58 significantly altered miRNAs in the TOF-noHF subset (33 up-regulated and 25 down-regulated) (Table 2b, P < 0.05, FDR adjusted) and 77 significantly deregulated miRNAs in the TOF-HF group (42 up-regulated and 35 down-regulated miRNAs) (Table 2c, P < 0.05), each compared to an age-matched healthy controls. Using hierarchic clustering with the euclidian distance measure, we analyzed how the TOF patients and controls were related to each other. For this task, we selected the 50 miRNAs with the highest variance of miRNA levels out of the 1205 miRNAs. Additional file 2: Figure S1 shows the resulting heatmap of the hierarchic clustering. We observed two distinct clusters between TOF-HF and matched controls Additional file 2: Figure S1A. The first cluster contains mostly controls and the second most of the TOF-HF patients. A more detailed distinction between the TOF-noHF and TOF-all subset, each matched to controls based on the clustering dendrogram were, however, not conclusive (Additional file 2: Figure S1B, C).

**Table 2:** The greatest fold change in miRNA expression levels in the blood of patient with TOF-all, TOF-noHF, and TOF-HF compared to age matched healthy controls as determined by microarray (unpaired two-tailed t test, >2.0-fold difference, and FDR, *P* < 0.05)

miRNA	*P* value	Corrected *P* value	Fold change	Regulation	AUC
A) TOF-all patients (n = 21) compared to matched healthy controls (n = 15)					
hsa-miR-1231	0.000379	0.001621	6.94	Up	0.83
hsa-miR-144*	0.000248	0.001198	4.82	Up	0.83
hsa-miR-505*	0.000557	0.002201	4.61	Up	0.83
hsa-miR-625	0.007441	0.017478	3.43	Up	0.77
hsa-miR-15b	0.000412	0.001738	3.03	Up	0.83
hsa-miR-218-1*	0.001196	0.003935	2.94	Up	0.87
hsa-miR-214	0.009782	0.021589	2.93	Up	0.75
hsa-let-7a	0.000013	0.000158	2.66	Up	0.91
hsa-let-7f	0.000643	0.002493	2.46	Up	0.84
hsa-miR-20b	0.000040	0.000335	2.41	Up	0.88
hsa-miR-1274b	0.000002	0.000056	2.38	Up	0.92
hsa-miR-218-2*	0.000000	0.000016	2.31	Up	0.95
hsa-let-7g	0.000008	0.000120	2.29	Up	0.90
hsa-miR-1274a	0.000138	0.000775	2.27	Up	0.86
hsa-let-7d	0.000012	0.000151	2.26	Up	0.92
hsa-miR-1233	0.000100	0.000613	2.23	Up	0.83
hsa-miR-183	0.000221	0.001107	2.21	Up	0.89
hsa-miR-20a	0.000099	0.000609	2.21	Up	0.86
hsa-miR-1288	0.000019	0.000195	2.20	Up	0.89
hsa-miR-138-1*	0.000006	0.000106	2.19	Up	0.90
hsa-miR-4286	0.003120	0.008467	2.18	Up	0.75
hsa-miR-3198	0.000335	0.001482	2.12	Up	0.83
hsa-miR-1181	0.000180	0.000939	2.09	Up	0.85
hsa-miR-34c-3p	0.000070	0.000488	2.07	Up	0.83
hsa-miR-3690	0.000000	0.000010	2.05	Up	0.99
hsa-miR-3125	0.000161	0.000862	2.04	Up	0.83
hsa-let-7b	0.011673	0.025119	2.00	Up	0.73
hsa-miR-181d	0.000014	0.000164	5.52	Down	0.05
hsa-miR-3653	0.002113	0.006271	4.28	Down	0.21
hsa-miR-206	0.000000	0.000018	2.82	Down	0.07
hsa-miR-3201	0.006986	0.016505	2.69	Down	0.16
hsa-miR-142-5p	0.000475	0.001951	2.60	Down	0.19
hsa-miR-181b	0.000777	0.002851	2.56	Down	0.17
hsa-miR-194	0.000000	0.000016	2.49	Down	0.06
hsa-miR-339-5p	0.000000	0.000016	2.43	Down	0.03
hsa-miR-26a	0.002104	0.006259	2.36	Down	0.20
hsa-miR-1268	0.011120	0.024188	2.33	Down	0.22
hsa-miR-595	0.000358	0.001548	2.31	Down	0.17
hsa-miR-151-3p	0.000000	0.000016	2.20	Down	0.04
hsa-miR-4271	0.000012	0.000155	2.19	Down	0.10
hsa-miR-1307	0.002101	0.006259	2.16	Down	0.25
hsa-miR-222	0.000258	0.001225	2.16	Down	0.14
hsa-miR-30c	0.000058	0.000432	2.15	Down	0.13
hsa-miR-620	0.000001	0.000034	2.11	Down	0.07
hsa-miR-623	0.000312	0.001418	2.09	Down	0.12
hsa-miR-30b	0.000336	0.001482	2.04	Down	0.15
hsa-miR-3689b*	0.000000	0.000020	2.04	Down	0.03
hsa-miR-186	0.009757	0.021573	2.03	Down	0.29
hsa-miR-191	0.000000	0.000021	2.00	Down	0.06
B) TOF-noHF patients (n = 18) compared to matched healthy controls (n = 15)					
hsa-miR-1231	0.000171	0.000930	8.72	Up	0.86
hsa-miR-144*	0.000156	0.000861	7.39	Up	0.85
hsa-miR-505*	0.000551	0.002170	4.54	Up	0.83
hsa-miR-214	0.009722	0.021817	3.33	Up	0.76
hsa-miR-625	0.014818	0.031162	3.23	Up	0.76
hsa-miR-218-1*	0.000048	0.000374	3.18	Up	0.95
hsa-miR-15b	0.000582	0.002270	3.00	Up	0.82
hsa-miR-1233	0.000043	0.000353	2.91	Up	0.86
hsa-let-7a	0.000011	0.000160	2.66	Up	0.92
hsa-miR-34c-3p	0.000112	0.000674	2.61	Up	0.85
hsa-let-7f	0.000702	0.002621	2.58	Up	0.84
hsa-miR-218-2*	0.000000	0.000019	2.58	Up	0.96
hsa-miR-138-1*	0.000011	0.000160	2.48	Up	0.90
hsa-miR-20b	0.000262	0.001258	2.48	Up	0.86
hsa-miR-20a	0.000391	0.001640	2.48	Up	0.84
hsa-miR-1181	0.000394	0.001643	2.34	Up	0.85
hsa-miR-4286	0.001466	0.004576	2.32	Up	0.78
hsa-miR-1274b	0.000003	0.000076	2.31	Up	0.91
hsa-let-7d	0.000019	0.000225	2.24	Up	0.91
hsa-miR-3690	0.000000	0.000019	2.23	Up	0.99
hsa-miR-1292	0.001251	0.004106	2.21	Up	0.75
hsa-miR-4258	0.000014	0.000189	2.20	Up	0.91
hsa-miR-1274a	0.000381	0.001627	2.18	Up	0.85
hsa-miR-1288	0.000089	0.000575	2.16	Up	0.87
hsa-miR-183	0.000371	0.001597	2.11	Up	0.87
hsa-miR-4319	0.000039	0.000333	2.10	Up	0.86
hsa-let-7g	0.000031	0.000290	2.08	Up	0.90
hsa-miR-124*	0.000023	0.000235	2.06	Up	0.94
hsa-miR-887	0.000004	0.000079	2.04	Up	0.91
hsa-miR-921	0.000003	0.000067	2.02	Up	0.90
hsa-miR-3907	0.000430	0.001761	2.02	Up	0.84
hsa-miR-196b*	0.000000	0.000019	2.01	Up	0.96
hsa-let-7b	0.006964	0.016423	2.01	Up	0.76
hsa-miR-181d	0.000015	0.000204	5.34	Down	0.06
hsa-miR-3653	0.005185	0.012829	3.99	Down	0.23
hsa-miR-206	0.000001	0.000035	2.88	Down	0.07
hsa-miR-194	0.000000	0.000019	2.70	Down	0.06
hsa-miR-3201	0.003859	0.010042	2.67	Down	0.14
hsa-miR-181b	0.000658	0.002515	2.62	Down	0.17
hsa-miR-339-5p	0.000000	0.000019	2.51	Down	0.03
hsa-miR-26a	0.001287	0.004168	2.38	Down	0.19
hsa-miR-595	0.000403	0.001676	2.36	Down	0.17
hsa-miR-151-3p	0.000000	0.000019	2.28	Down	0.04
hsa-miR-1307	0.001320	0.004219	2.24	Down	0.23
hsa-miR-3689b*	0.000003	0.000069	2.15	Down	0.03
hsa-miR-191	0.000001	0.000025	2.15	Down	0.06
hsa-miR-142-5p	0.001336	0.004247	2.14	Down	0.21
hsa-miR-222	0.000341	0.001515	2.12	Down	0.15
hsa-miR-620	0.000001	0.000035	2.11	Down	0.06
hsa-miR-3129	0.000021	0.000234	2.08	Down	0.09
hsa-miR-186	0.025390	0.049030	2.08	Down	0.33
hsa-miR-30c	0.000279	0.001306	2.07	Down	0.16
hsa-miR-4271	0.000009	0.000143	2.06	Down	0.10
hsa-miR-140-3p	0.000004	0.000079	2.06	Down	0.05
hsa-miR-190b	0.000024	0.000241	2.05	Down	0.08
hsa-miR-623	0.000389	0.001640	2.04	Down	0.13
hsa-miR-1262	0.000000	0.000020	2.03	Down	0.04
hsa-miR-30d	0.000018	0.000218	2.01	Down	0.10
C) TOF-HF patients (n = 3) compared to matched healthy controls (n = 3)					
hsa-miR-625	0.000868	0.040212	11.77	Up	
hsa-miR-183*	0.041803	0.150816	6.16	Up	
hsa-miR-183	0.016584	0.093166	6.08	Up	
hsa-miR-505*	0.020963	0.105695	5.66	Up	
hsa-miR-214	0.000609	0.032735	4.60	Up	
hsa-miR-30e*	0.015156	0.089838	4.59	Up	
hsa-let-7d	0.003245	0.062067	4.44	Up	
hsa-miR-340*	0.045108	0.154000	3.94	Up	
hsa-miR-28-5p	0.009640	0.081007	3.81	Up	
hsa-miR-15b	0.000038	0.015689	3.81	Up	
hsa-miR-151-5p	0.000976	0.040600	3.77	Up	
hsa-let-7a	0.043951	0.153067	3.51	Up	
hsa-miR-1271	0.001328	0.044227	3.34	Up	
hsa-miR-192	0.001655	0.044227	3.30	Up	
hsa-miR-20b	0.011702	0.083679	3.03	Up	
hsa-miR-23b	0.001013	0.040600	3.02	Up	
hsa-miR-148b	0.014520	0.089728	2.95	Up	
hsa-miR-339-3p	0.006304	0.071105	2.89	Up	
hsa-let-7g	0.016852	0.093166	2.85	Up	
hsa-miR-660	0.010161	0.081084	2.71	Up	
hsa-miR-20a	0.035924	0.135700	2.69	Up	
hsa-miR-502-3p	0.000625	0.032735	2.60	Up	
hsa-miR-7-1*	0.002495	0.054656	2.59	Up	
hsa-miR-3677	0.014330	0.089658	2.55	Up	
hsa-miR-1274b	0.027516	0.119268	2.49	Up	
hsa-miR-218-2*	0.004335	0.064409	2.49	Up	
hsa-miR-18a	0.006475	0.071578	2.38	Up	
hsa-miR-330-3p	0.004056	0.064314	2.36	Up	
hsa-miR-1274a	0.004302	0.064409	2.32	Up	
hsa-miR-29a	0.005922	0.070658	2.31	Up	
hsa-miR-138-1*	0.000786	0.039488	2.30	Up	
hsa-miR-744	0.000059	0.015689	2.28	Up	
hsa-miR-132	0.007974	0.075066	2.19	Up	
hsa-miR-1181	0.000288	0.024545	2.19	Up	
hsa-miR-29c	0.026647	0.117616	2.17	Up	
hsa-miR-3195	0.029339	0.121421	2.16	Up	
hsa-miR-24	0.007116	0.073136	2.13	Up	
hsa-miR-3125	0.004598	0.064409	2.10	Up	
hsa-miR-3127	0.004650	0.064409	2.05	Up	
hsa-miR-192*	0.000160	0.022287	2.04	Up	
hsa-miR-1288	0.004046	0.064314	2.03	Up	
hsa-miR-200c	0.000842	0.040212	2.01	Up	
hsa-miR-3653	0.000338	0.024640	9.35	Down	
hsa-miR-181d	0.001044	0.040600	6.90	Down	
hsa-miR-1268	0.001753	0.044227	4.61	Down	
hsa-miR-222	0.011501	0.083679	4.47	Down	
hsa-miR-623	0.007440	0.073136	4.14	Down	
hsa-miR-574-5p	0.000088	0.017647	3.58	Down	
hsa-miR-92a	0.000166	0.022287	3.37	Down	
hsa-miR-99b	0.025374	0.114210	3.36	Down	
hsa-miR-548f	0.000032	0.015689	3.06	Down	
hsa-miR-564	0.003564	0.063109	2.88	Down	
hsa-miR-320a	0.025932	0.115734	2.87	Down	
hsa-miR-206	0.000138	0.022287	2.80	Down	
hsa-miR-181a	0.017001	0.093166	2.78	Down	
hsa-miR-3663-5p	0.014980	0.089808	2.77	Down	
hsa-miR-595	0.005164	0.068567	2.66	Down	
hsa-miR-942	0.029072	0.121421	2.65	Down	
hsa-miR-494	0.003770	0.063109	2.61	Down	
hsa-miR-423-5p	0.007923	0.075066	2.59	Down	
hsa-miR-142-5p	0.025401	0.114210	2.52	Down	
hsa-miR-1228*	0.000065	0.015689	2.52	Down	
hsa-miR-532-3p	0.016610	0.093166	2.41	Down	
hsa-miR-670	0.001332	0.044227	2.41	Down	
hsa-miR-3191	0.007583	0.073136	2.38	Down	
hsa-miR-452*	0.003478	0.062543	2.35	Down	
hsa-miR-940	0.001816	0.044648	2.28	Down	
hsa-miR-30c	0.001368	0.044227	2.27	Down	
hsa-miR-4316	0.013200	0.087753	2.25	Down	
hsa-miR-145	0.010675	0.082458	2.25	Down	
hsa-miR-1306	0.005642	0.068843	2.19	Down	
hsa-miR-186	0.007169	0.073136	2.15	Down	
hsa-miR-3147	0.000301	0.024545	2.14	Down	
hsa-miR-425	0.005463	0.068567	2.08	Down	
hsa-miR-3686	0.007328	0.073136	2.08	Down	
hsa-miR-874	0.023395	0.112764	2.02	Down	
hsa-miR-920	0.029734	0.121421	2.01	Down	

*TOF-all* all patients with Tetralogy of Fallot, *TOF-noHF* TOF patients without heart failure, *TOF-HF* TOF patients with heart failure, *AUC* area under the receiver operating characteristic curve

* *P* ≤ 0.05

### Validation of candidate miRNAs by RT-qPCR

**Fig. 1:**
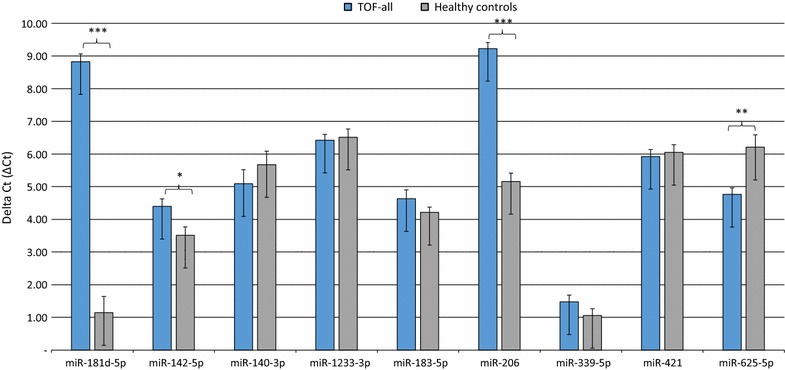
Validation of nine differentially expressed miRNAs in the blood of all TOF patients (TOF-all) (n = 37) compared to healthy controls (n = 15) as determined by RT-qPCR (*P* < 0.05). Mean ΔCt TOF-all and healthy controls (lower ΔCt, higher expression level). RNAU6B as an endogenous control for normalization, unpaired-two-tailed t tests and ±standard deviation (STDV) were used to evaluate differences in expression. * *P* ≤ 0.05; ** *P* ≤ 0.01; *** *P* ≤ 0.001

**Fig. 2:**
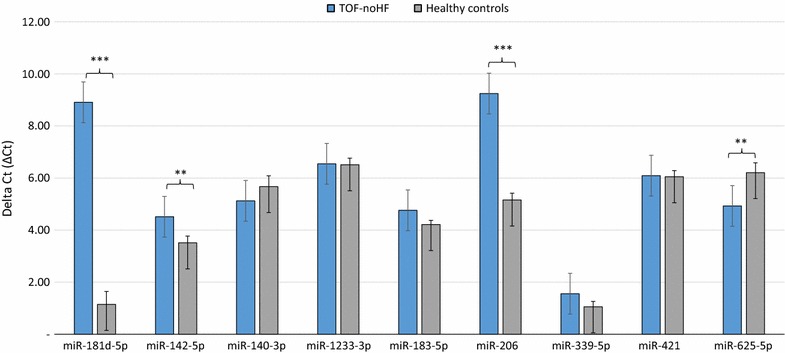
Validation of nine differentially expressed miRNAs in the blood of TOF patients with no signs of right heart failure (TOF-noHF) (n = 34) compared to healthy controls (n = 15) as determined by RT-qPCR (*P* < 0.05). Mean ΔCt TOF-noHF and healthy controls (lower ΔCt, higher expression level). RNAU6B as an endogenous control for normalization, unpaired-two-tailed t tests and ±standard deviation (STDV) were used to evaluate differences in expression. ** *P* ≤ 0.01; *** *P* ≤ 0.001

**Fig. 3:**
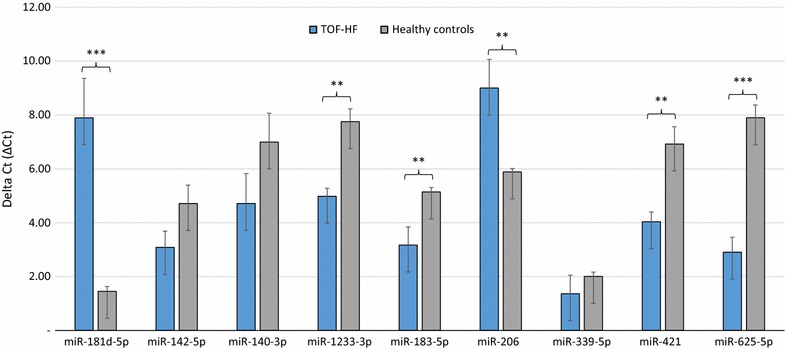
Validation of nine differentially expressed miRNAs in the blood of TOF patients with right heart failure (TOF-HF) (n = 3) compared to healthy controls (n = 3) as determined by RT-qPCR (*P* < 0.05). Mean ΔCt TOF-HF and healthy controls (lower ΔCt, higher expression level). RNAU6B as an endogenous control for normalization, unpaired-two-tailed t tests and ±standard deviation (STDV) were used to evaluate differences in expression. ** *P* ≤ 0.01; *** *P* ≤ 0.001

Using RT-qPCR, the validation of microarray data was performed to re-examine the expression level of 9 miRNAs, namely miR-181d-5p, miR-142-5p, miR-140-3p, miR-1233-3p, miR-183-5p, miR-206, miR-339-5p, miR-421 and miR-625-5p by RT-qPCR. These miRNAs were chosen based on their differential expression level in each patient group and matched controls and based on their known associations with cardiovascular diseases [9, 24–34]. In detail, we selected two miRNAs with high fold changes among the up-regulated (miR-625-5p and miR-183-5p) and four miRNAs with high fold changes among the down-regulated ones (miR-181d-5p, miR-206, miR-142-5p and miR-339-5p). These miRNAs were shared in the three comparisons. In addition, we selected three miRNAs (miR-1233-3p, miR-140-3p and miR-421) with low or moderate expression levels in the three comparisons and miR-421 that had been identified in TOF [9]. In the first validation, a total of 37 TOF-all patients and 15 healthy controls were included in the analysis. The RT-qPCR showed the same direction of expression changes as the microarray analysis for six miRNAs namely miR-181d-5p, miR-142-5p, miR-1233-3p, miR-206, miR-339-5p and miR-625-5p. The significance of the differences in the expression was confirmed for four of the miRNAs, including three down-regulated miRNAs namely miR-181d-5p, miR-140-3p and miR-206 and one up-regulated miRNA namely miR-625-5p (*P* < 0.05) (Fig. 1). In the second validation step, a total of 34 TOF-noHF patients and 15 control samples were included. The RT-qPCR showed the same direction of expression changes as the microarray analysis for five miRNAs namely miR-181d-5p, miR-142-5p, miR-206, miR-339-5p and miR-625-5p. The significance of the differences in the expression was confirmed for four of the miRNAs, including three down-regulated miRNAs namely miR-181d-5p, miR-140-3p and miR-206 and one up-regulated miRNA namely miR-625-5p) (*P* < 0.05) (Fig. 2). In the third validation step, a total of 3 TOF-HF patients and matched controls were included. The RT-qPCR showed the same direction of expression changes as the microarray analysis for six miRNAs namely miR-181d-5p, miR-1233-3p, miR-183-5p, miR-206, miR-421 and miR-625-5p. The significance of the differences in the expression was confirmed for six of the miRNAs, including two down-regulated miRNAs namely miR-181d-5p and miR-206 and four up-regulated miRNA namely miR-1233-3p, miR-183-5p, miR-421 and miR-625-5p) (*P* < 0.05) (Fig. 3). Together, the three miRNAs namely miR-181d-5p, miR-206 and miR-625-5p were significantly deregulated in all subgroups of TOF patients.

### Relation to clinical variables

**Fig. 4:**
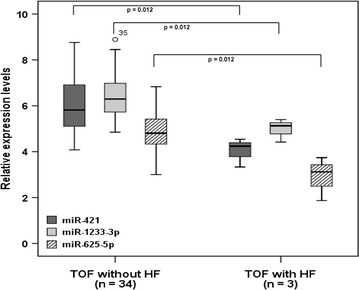
Boxplots representing relative expression levels of miR-421, miR-1233-3p and miR-625-5p in TOF patients with and without symptomatic right heart failure

No correlations were found between six of the nine validated miRNAs by RT-qPCR and various clinical parameters such as NYHA class, blood pressure or transcutaneous oxygen saturation. However, weak correlations were observed between miR-421 and miR-1233-5p and right ventricular volumes, ejection fraction as well as hsTNT (Table 3). Moreover, differential expression levels of miR-421, miR-1233-3p and miR-625-5p were found in TOF-noHF and TOF-HF patients with significantly reduced expression levels in TOF patients with symptomatic right heart failure (Fig. 4). No differences in expression levels of these 3 miRNAs were seen for the presence of residual lesions associated with pressure or volume overload of the right ventricle.

**Table 3:** Relation of miR-421 and miR-1233-3p with different variables in all TOF patients

Variables	miR-421	*P* value	miR-1233-3p	*P* value
	r		r	
Age at follow-up (year)	0.258	NS	0.158	NS
NYHA functional class	−0.175	NS	−0.120	NS
Systolic blood pressure	−0.097	NS	0.020	NS
Diastolic blood pressure	0.081	NS	0.072	NS
Transcutaneous oxygen saturation at rest	0.157	NS	0.168	NS
Ejection fraction of RV	0.398	0.02	0.403	0.018
Enddiastolic volume of RV	−0.364	0.034	−0.384	0.025
Endsystolic volume of RV	−0.405	0.017	−0.411	0.016
Ejection fraction of LV	0.030	NS	0.008	NS
Enddiastolic volume of LV	−0.282	NS	−0.235	NS
Endsystolic volume of LV	−0.264	NS	−0.230	NS
VTI above aortic valve	0.278	NS	0.295	NS
NT-proBNP	0.110	NS	0.059	NS
High sensitive Troponin T	−0.488	0.003	−0.493	0.002

Spearman rank correlation

*NYHA* New York Heart Association, *RV* right ventricle, *LV* left ventricle, *VTI* velocity time integral, *NS* not significant

### Diagnostic accuracy of the validated miRNAs

To evaluate whether specific miRNAs might be capable of discriminating patients from controls, ROC curve analysis was performed for the validated three miRNAs between TOF-all patients, TOF-noHF patients and healthy controls. Compared with controls, the AUCs for miR-181d-5p, miR-206 and miR-625-5p in TOF-all patients were 0.9874, 0.9928 and 0.7694, respectively and in TOF-noHF patients 0.9902, 0.9941 and 0.7490, respectively. Together, these results indicate that these three identified miRNAs can discriminate between TOF patients and healthy controls with high accuracy.

### Comparative pathway analysis

To gain insights into the potential impact of the three validated miRNAs (miR-181d-5p, miR-206 and miR-625-5p) in regulating target genes, we applied miRTargetLink [23]. Our analysis suggested a tripartite network, containing besides the 3 miRNAs, 11 genes that are targeted by two or more of the selected miRNAs in the ‘Strong + Weak’ category. Strong interaction was observed between miR-181d-5p and BCL2 and is highlighted in ‘Green’ in the resulting network (Additional file 3: Figure S2).

## Discussion

MiRNAs are known to be involved in various pathophysiologies and cardiovascular disease including CHD [8, 14, 15, 17]. Recent studies have shown that specific patterns of miRNAs are specifically involved in the development of CHD and TOF [9, 13] and that expression levels of altered miRNAs may also be associated with measures of ventricular function [16] or even disease progression and outcome [35]. In this study, we identified an altered miRNA expression profile in TOF patients with and without symptomatic right heart failure when compared with age-matched healthy controls by microarray and further validated by RT-qPCR analyses. The diagnostic accuracy of only three of the validated miRNAs namely miR-181d-5p, miR-206 and miR-625-5p by ROC analysis was high with AUCs of 0.987, 0.993 and 0.769 respectively in all TOF patients and 0.990, 0.994 and 0.749 respectively in the subset of TOF patients without symptomatic right heart failure. These results indicate the high diagnostic accuracy of miR-181d-5p and miR-206 and a moderate accuracy of miR-625-5p in differentiating TOF patients from healthy controls. It is of note, however, that miR-625-5p showed the highest fold change between healthy controls and the subset of TOF patients with symptomatic right heart failure. The miRNA expression profile found in the blood of surgically repaired TOF patients is different to that found in RVOT tissue of infants with TOF [9, 10]. In the latter group, altered expression levels of miR-421, miR-1275, miR-27b, miR-1201 and miR-122 have been reported. MiR-940 was found as most down-regulated and miR-204 as most up-regulated miRNA [9, 10, 13]. However, miRNA in myocardial tissue of infants undergoing surgical repair reflects a completely different situation than circulating miRNAs in TOF patients late after surgical repair usually associated with the presence of different residual lesions and loading conditions of the right ventricle. Nevertheless, miR-421 was also significantly altered in the blood of patients after long-term repair of TOF suggesting a specific role of miR-421 in the early pathology of TOF as well as during long-term follow-up. For further analysis of the patient group, we additionally selected three miRNAs (miR-1233-3p, miR-140-3p and miR-421) with low or moderate expression levels in the three comparisons and miR-421 that had been identified in myocardial tissue of TOF patients [9]. We found that expression levels of circulating miR-421, miR-1233-3p and miR-625-5p were significantly lower in TOF patients with as compared to those without symptomatic right heart failure indicating a potential role of these miRNAs in identifying disease progression in TOF patients. However, no differences in expression levels of these miRNAs were seen for the presence of residual lesions associated with right ventricular pressure overload such as significant restenosis of the RVOT or right ventricular volume overload due to significant pulmonary regurgitation. In our patient group, symptomatic right heart failure was also associated with left ventricular dysfunction as indicated by the reduced left ventricular ejection fraction, velocity time integral above the aortic valve and markedly elevated NT-proBNP levels. It is known from previous studies that LV dysfunction may also be present late after TOF repair due to various reasons such as RV enlargement/dysfunction associated with interventricular mechanical or electrical dyssynchrony [3, 36, 37] or longstanding cyanosis prior to corrective surgery [2]. This is consistent with our findings of increased right ventricular volumes and older age at corrective surgery in the TOF-HF group. Recently, it has been shown that levels of circulating miRNAs are declining with progression or increasing acuity of heart failure in patients with left heart failure [35]. Although a different expression profile was found in these patients, the results of our study with lower miRNA expression levels found in TOF-HF patients are in agreement with that study and thus may also be indicative of disease progression in TOF patients. However, these results should be confirmed in a larger cohort of patients, especially in the TOF-HF subset. Bioinformatics analysis helped to gain insights into the potential impact of the three validated miRNAs (miR-181d-5p, miR-206 and miR-625-5p) on target genes (Additional file 3: Figure S2). Among the predicted target genes was B-cell lymphoma 2 (Bcl-2), which plays a critical role in the inhibition of apoptosis [38], Bcl-2 expressed in myocytes of the human heart with infarction and participates in the protection or acceleration of cellular damage after infarction [39]. Similarly, Insulin-like growth factor 1 receptor (IGF1R), protects the heart in settings of heart diseases [40] and plays a role in proliferation, cardiac differentiation, and decreases apoptosis in human heart [41]. Bromodomain Adjacent to Zinc Finger Domain 2A (BAZ2A), plays an important role in the transcription deregulation in hypertrophy and heart failure [42].

## Conclusion

Altered expression levels of circulating miRNAs can be found in TOF patients late after surgical repair and are different to those seen in the right ventricular myocardium of infants with TOF. Expression levels of miR-421, miR-1233-3p and miR-625-5p are lower in TOF patients with symptomatic right heart failure and thus may indicate disease progression in these patients.

## Additional files

**Additional file 1: Table S1.** Expression of all significantly miRNAs in the blood samples of TOF-all, TOF-noHF, and TOF-HF patients compared with those of controls (age-matched) as determined by microarray (Unpaired two-tailed t test, P < 0.05).

**Additional file 2: Figure S1.** Unsupervised hierarchical clustering (Euclidian distance, complete linkage) of the patients compared to matched controls based on expression of the 50 with the highest variance. 1A) the first cluster contains mostly controls and the second most of the TOF-HF patients. 1B) and 1C) A more detailed distinction between the TOF-noHF and TOF-all subset, each matched to controls based on the clustering dendrogram were, however, not conclusive.

**Additional file 3: Figure S2.** Target network: for the three validated miRNAs in brown, target genes are presented in blue.

## Abbreviations

BAZ2A: adjacent to zinc finger domain 2A
Bcl-2: B-cell lymphoma 2
CHD: congenital heart defect
DMSO: dimethyl sulfoxide
GAPDH: glyceraldehyde 3-phosphate dehydrogenase
IGF1R: insulin-like growth factor 1 receptor
LV: left ventricle
miRNA: microRNA
NS: not significant
NT-proBNP: N-terminal pro B-type natriuretic peptide
NYHA: New York Heart Association
RT-qPCR: quantitative reverse transcription-polymerase chain reaction
RV: right ventricle
RVOT: right ventricular outflow tract
TGS: total gene signals
TOF: Tetralogy of Fallot
TOF-all: Tetralogy of Fallot-all patients
TOF-HF: Tetralogy of Fallot patients with symptomatic right heart failure
TOF-noHF: Tetralogy of Fallot patients without symptomatic right heart failure
VSD: ventricular septal defect
VTI: velocity time integral
