# Supplementary material for: Analysis of circulating microRNAs in patients with repaired Tetralogy of Fallot with and without heart failure
Source: J Transl Med. 2017 Jul 10;15:156. doi: 10.1186/s12967-017-1255-z (PMC5504636; doi:10.1186/s12967-017-1255-z)
Supplement: Supplementary file 1 — Additional file 1: Table S1. Expression of all significantly miRNAs in the blood samples of TOF-all, TOF-noHF, and TOF-HF patients compared with those of controls (age-matched) as determined by microarray (Unpaired two-tailed t test, P < 0.05). [file 12967_2017_1255_MOESM1_ESM.docx]

**Table S1:** Expression of all significantly miRNAs in the blood samples of TOF-all. TOF-noHF and TOF-HF patients compared with those of controls (age-matched) as determined by microarray (Unpaired two-tailed t-test. *P*< 0.05)

| **A) TOF-all patients (n=21) compared to matched healthy controls (n=15)** | | | | | |
| --- | --- | --- | --- | --- | --- |
| **miRNA** | **Fold Change** | **Regulation** | **T test** | **T test Adj** | **AUC** |
| hsa-miR-1231 | 6.94 | Up | 0.00037933 | 0.00162091 | 0.83 |
| hsa-miR-144* | 4.82 | Up | 0.00024848 | 0.00119766 | 0.83 |
| hsa-miR-505* | 4.61 | Up | 0.00055722 | 0.00220149 | 0.83 |
| hsa-miR-625 | 3.43 | Up | 0.00744082 | 0.01747796 | 0.77 |
| hsa-miR-15b | 3.03 | Up | 0.00041249 | 0.00173792 | 0.83 |
| hsa-miR-218-1* | 2.94 | Up | 0.00119596 | 0.00393491 | 0.87 |
| hsa-miR-214 | 2.93 | Up | 0.00978223 | 0.021589 | 0.75 |
| hsa-let-7a | 2.66 | Up | 1.27E-05 | 0.00015797 | 0.91 |
| hsa-let-7f | 2.46 | Up | 0.00064348 | 0.00249322 | 0.84 |
| hsa-miR-20b | 2.41 | Up | 3.98E-05 | 0.00033517 | 0.88 |
| hsa-miR-1274b | 2.38 | Up | 2.06E-06 | 5.64E-05 | 0.92 |
| hsa-miR-218-2* | 2.31 | Up | 6.59E-08 | 1.58E-05 | 0.95 |
| hsa-let-7g | 2.29 | Up | 8.21E-06 | 0.00011962 | 0.90 |
| hsa-miR-1274a | 2.27 | Up | 0.00013753 | 0.00077527 | 0.86 |
| hsa-let-7d | 2.26 | Up | 1.17E-05 | 0.00015096 | 0.92 |
| hsa-miR-1233 | 2.23 | Up | 9.97E-05 | 0.00061279 | 0.83 |
| hsa-miR-183 | 2.21 | Up | 0.00022142 | 0.00110709 | 0.89 |
| hsa-miR-20a | 2.21 | Up | 9.86E-05 | 0.00060932 | 0.86 |
| hsa-miR-1288 | 2.20 | Up | 1.89E-05 | 0.00019481 | 0.89 |
| hsa-miR-138-1* | 2.19 | Up | 6.28E-06 | 0.0001065 | 0.90 |
| hsa-miR-4286 | 2.18 | Up | 0.00311989 | 0.00846727 | 0.75 |
| hsa-miR-3198 | 2.12 | Up | 0.00033513 | 0.00148204 | 0.83 |
| hsa-miR-1181 | 2.09 | Up | 0.00017984 | 0.00093923 | 0.85 |
| hsa-miR-34c-3p | 2.07 | Up | 6.96E-05 | 0.00048786 | 0.83 |
| hsa-miR-3690 | 2.05 | Up | 8.17E-09 | 9.84E-06 | 0.99 |
| hsa-miR-3125 | 2.04 | Up | 0.0001609 | 0.00086169 | 0.83 |
| hsa-let-7b | 2.00 | Up | 0.0116734 | 0.02511866 | 0.73 |
| hsa-miR-3667-5p | 1.95 | Up | 0.00412091 | 0.01047614 | 0.77 |
| hsa-miR-1271 | 1.93 | Up | 0.0035223 | 0.00922689 | 0.77 |
| hsa-miR-183* | 1.89 | Up | 0.01597013 | 0.0330086 | 0.70 |
| hsa-miR-4279 | 1.89 | Up | 3.74E-07 | 1.88E-05 | 0.93 |
| hsa-miR-921 | 1.89 | Up | 3.65E-07 | 1.88E-05 | 0.91 |
| hsa-miR-554 | 1.85 | Up | 0.00032048 | 0.00143616 | 0.84 |
| hsa-miR-3918 | 1.84 | Up | 6.29E-05 | 0.00045575 | 0.84 |
| hsa-miR-28-5p | 1.83 | Up | 0.00852072 | 0.01937258 | 0.76 |
| hsa-miR-3124 | 1.82 | Up | 0.00110073 | 0.00368438 | 0.84 |
| hsa-miR-124* | 1.82 | Up | 1.73E-05 | 0.00018783 | 0.93 |
| hsa-miR-943 | 1.82 | Up | 1.38E-06 | 4.16E-05 | 0.91 |
| hsa-miR-3935 | 1.81 | Up | 0.01879595 | 0.03793822 | 0.81 |
| hsa-miR-411* | 1.81 | Up | 1.20E-05 | 0.00015213 | 0.88 |
| hsa-miR-151-5p | 1.80 | Up | 0.00809206 | 0.01850272 | 0.72 |
| hsa-miR-449b* | 1.78 | Up | 0.00010023 | 0.00061306 | 0.84 |
| hsa-miR-2114* | 1.78 | Up | 9.34E-06 | 0.00012645 | 0.92 |
| hsa-miR-27a | 1.78 | Up | 0.00233363 | 0.00671127 | 0.78 |
| hsa-miR-196b* | 1.78 | Up | 1.83E-07 | 1.58E-05 | 0.96 |
| hsa-miR-4258 | 1.78 | Up | 5.35E-06 | 9.49E-05 | 0.91 |
| hsa-miR-3907 | 1.77 | Up | 0.00066197 | 0.0025243 | 0.83 |
| hsa-miR-1305 | 1.77 | Up | 0.00023899 | 0.00117066 | 0.86 |
| hsa-miR-4319 | 1.75 | Up | 8.75E-05 | 0.00056065 | 0.83 |
| hsa-miR-3677 | 1.74 | Up | 8.76E-07 | 3.35E-05 | 0.93 |
| hsa-miR-744* | 1.73 | Up | 8.67E-06 | 0.00012007 | 0.91 |
| hsa-miR-2355-5p | 1.72 | Up | 8.35E-06 | 0.00011962 | 0.89 |
| hsa-miR-331-5p | 1.71 | Up | 3.55E-06 | 8.20E-05 | 0.90 |
| hsa-miR-891a | 1.71 | Up | 1.47E-07 | 1.58E-05 | 0.95 |
| hsa-miR-887 | 1.71 | Up | 3.94E-06 | 8.34E-05 | 0.90 |
| hsa-miR-431 | 1.71 | Up | 0.00026207 | 0.00123839 | 0.83 |
| hsa-miR-2277-3p | 1.70 | Up | 7.47E-05 | 0.00051402 | 0.86 |
| hsa-let-7i | 1.69 | Up | 0.00544239 | 0.0134112 | 0.81 |
| hsa-miR-1251 | 1.68 | Up | 5.13E-06 | 9.49E-05 | 0.90 |
| hsa-miR-363 | 1.68 | Up | 5.23E-06 | 9.49E-05 | 0.89 |
| hsa-miR-657 | 1.68 | Up | 4.30E-05 | 0.00035587 | 0.88 |
| hsa-miR-588 | 1.67 | Up | 1.47E-05 | 0.0001644 | 0.87 |
| hsa-miR-505 | 1.66 | Up | 0.02218439 | 0.04318609 | 0.72 |
| hsa-miR-192* | 1.66 | Up | 4.97E-06 | 9.49E-05 | 0.89 |
| hsa-miR-1469 | 1.64 | Up | 0.00102564 | 0.00348148 | 0.77 |
| hsa-miR-668 | 1.64 | Up | 4.58E-06 | 9.43E-05 | 0.89 |
| hsa-miR-1273c | 1.63 | Up | 1.90E-05 | 0.00019481 | 0.87 |
| hsa-let-7c* | 1.63 | Up | 1.78E-07 | 1.58E-05 | 0.93 |
| hsa-miR-1914 | 1.63 | Up | 3.73E-05 | 0.00031653 | 0.85 |
| hsa-miR-663 | 1.62 | Up | 0.00502227 | 0.0125038 | 0.79 |
| hsa-miR-4321 | 1.62 | Up | 0.00132605 | 0.0042497 | 0.80 |
| hsa-miR-1260b | 1.62 | Up | 0.00077832 | 0.00285069 | 0.83 |
| hsa-miR-675* | 1.61 | Up | 6.95E-06 | 0.0001106 | 0.89 |
| hsa-miR-33a* | 1.61 | Up | 4.32E-07 | 2.00E-05 | 0.95 |
| hsa-miR-487b | 1.61 | Up | 0.00011939 | 0.00070523 | 0.85 |
| hsa-miR-4262 | 1.61 | Up | 2.51E-05 | 0.00023632 | 0.88 |
| hsa-miR-934 | 1.60 | Up | 0.02007416 | 0.03991643 | 0.70 |
| hsa-miR-4275 | 1.60 | Up | 2.16E-07 | 1.58E-05 | 0.96 |
| hsa-miR-193a-3p | 1.59 | Up | 9.18E-07 | 3.35E-05 | 0.91 |
| hsa-miR-299-5p | 1.59 | Up | 0.002934 | 0.00809031 | 0.80 |
| hsa-miR-466 | 1.58 | Up | 0.00227578 | 0.00660797 | 0.79 |
| hsa-miR-3181 | 1.58 | Up | 0.00394698 | 0.01012598 | 0.73 |
| hsa-miR-4301 | 1.58 | Up | 2.23E-07 | 1.58E-05 | 0.95 |
| hsa-miR-4305 | 1.57 | Up | 6.99E-07 | 2.91E-05 | 0.94 |
| hsa-miR-4252 | 1.57 | Up | 0.00013889 | 0.00077527 | 0.84 |
| hsa-miR-20b* | 1.57 | Up | 1.16E-06 | 3.77E-05 | 0.93 |
| hsa-miR-676* | 1.57 | Up | 1.74E-06 | 4.87E-05 | 0.93 |
| hsa-miR-1913 | 1.56 | Up | 0.00010367 | 0.00063093 | 0.84 |
| hsa-miR-3941 | 1.55 | Up | 0.00383448 | 0.00993667 | 0.77 |
| hsa-miR-3127 | 1.55 | Up | 0.00474451 | 0.01186126 | 0.74 |
| hsa-miR-4293 | 1.55 | Up | 0.00319705 | 0.00858007 | 0.77 |
| hsa-miR-633 | 1.55 | Up | 1.20E-06 | 3.80E-05 | 0.90 |
| hsa-miR-148a* | 1.55 | Up | 9.11E-07 | 3.35E-05 | 0.92 |
| hsa-miR-107 | 1.54 | Up | 0.00064864 | 0.00250516 | 0.83 |
| hsa-miR-3126-5p | 1.54 | Up | 9.46E-07 | 3.35E-05 | 0.95 |
| hsa-miR-550b | 1.54 | Up | 0.0001689 | 0.00089265 | 0.83 |
| hsa-miR-3939 | 1.54 | Up | 1.66E-06 | 4.77E-05 | 0.92 |
| hsa-miR-18a | 1.53 | Up | 0.00332331 | 0.00878759 | 0.75 |
| hsa-miR-647 | 1.52 | Up | 2.76E-05 | 0.00025174 | 0.88 |
| hsa-miR-600 | 1.51 | Up | 1.82E-05 | 0.0001926 | 0.90 |
| hsa-miR-596 | 1.51 | Up | 6.06E-05 | 0.0004423 | 0.83 |
| hsa-miR-3139 | 1.51 | Up | 1.52E-07 | 1.58E-05 | 0.95 |
| hsa-miR-3184 | 1.51 | Up | 1.05E-06 | 3.61E-05 | 0.92 |
| hsa-miR-451 | 1.51 | Up | 0.02519371 | 0.04803548 | 0.69 |
| hsa-let-7g* | 1.51 | Up | 3.77E-06 | 8.20E-05 | 0.89 |
| hsa-miR-4285 | 1.51 | Up | 0.00441833 | 0.01109184 | 0.86 |
| hsa-miR-4269 | 1.50 | Up | 7.20E-05 | 0.00050128 | 0.88 |
| hsa-miR-542-5p | 1.50 | Up | 0.00013961 | 0.00077527 | 0.85 |
| hsa-miR-3622a-3p | 1.50 | Up | 3.45E-06 | 8.20E-05 | 0.90 |
| hsa-miR-409-5p | 1.50 | Up | 5.90E-05 | 0.00043629 | 0.88 |
| hsa-miR-362-3p | 1.49 | Up | 0.00311895 | 0.00846727 | 0.78 |
| hsa-miR-631 | 1.49 | Up | 0.00785862 | 0.01817589 | 0.79 |
| hsa-miR-155* | 1.49 | Up | 0.00053767 | 0.00214534 | 0.86 |
| hsa-miR-502-3p | 1.49 | Up | 0.00112893 | 0.00374754 | 0.80 |
| hsa-miR-582-3p | 1.49 | Up | 2.80E-05 | 0.00025399 | 0.86 |
| hsa-miR-491-5p | 1.48 | Up | 0.00804757 | 0.01843597 | 0.77 |
| hsa-miR-892b | 1.48 | Up | 0.00201442 | 0.00608748 | 0.79 |
| hsa-miR-621 | 1.48 | Up | 6.46E-06 | 0.00010807 | 0.90 |
| hsa-miR-205 | 1.48 | Up | 0.00094653 | 0.0033156 | 0.80 |
| hsa-miR-1296 | 1.47 | Up | 0.0007336 | 0.00275387 | 0.85 |
| hsa-miR-4277 | 1.47 | Up | 0.00087929 | 0.00311632 | 0.77 |
| hsa-miR-3944 | 1.47 | Up | 1.79E-05 | 0.00019233 | 0.90 |
| hsa-miR-1292 | 1.47 | Up | 0.00102566 | 0.00348148 | 0.75 |
| hsa-miR-4330 | 1.47 | Up | 0.00079232 | 0.00287572 | 0.77 |
| hsa-miR-24-2* | 1.47 | Up | 0.00209678 | 0.00625904 | 0.80 |
| hsa-miR-1291 | 1.46 | Up | 0.01611418 | 0.03324929 | 0.73 |
| hsa-miR-34b | 1.46 | Up | 2.17E-05 | 0.00021074 | 0.88 |
| hsa-miR-2115 | 1.46 | Up | 3.03E-05 | 0.0002724 | 0.87 |
| hsa-miR-378b | 1.46 | Up | 0.00859579 | 0.01950644 | 0.74 |
| hsa-miR-455-3p | 1.45 | Up | 3.69E-06 | 8.20E-05 | 0.91 |
| hsa-miR-1224-3p | 1.45 | Up | 7.87E-05 | 0.00053003 | 0.86 |
| hsa-miR-3126-3p | 1.45 | Up | 2.95E-06 | 7.62E-05 | 0.94 |
| hsa-miR-1286 | 1.45 | Up | 6.03E-05 | 0.0004423 | 0.88 |
| hsa-miR-541 | 1.45 | Up | 0.00039962 | 0.00168962 | 0.79 |
| hsa-miR-885-5p | 1.45 | Up | 8.30E-05 | 0.0005377 | 0.89 |
| hsa-miR-708 | 1.45 | Up | 0.00051724 | 0.00208451 | 0.84 |
| hsa-miR-487a | 1.44 | Up | 6.98E-06 | 0.0001106 | 0.92 |
| hsa-miR-3160 | 1.44 | Up | 5.47E-05 | 0.00041697 | 0.84 |
| hsa-miR-3158 | 1.44 | Up | 1.82E-05 | 0.0001926 | 0.89 |
| hsa-miR-2277-5p | 1.44 | Up | 0.00065554 | 0.00250771 | 0.84 |
| hsa-miR-891b | 1.44 | Up | 0.00099323 | 0.00340981 | 0.83 |
| hsa-miR-1910 | 1.43 | Up | 0.021319 | 0.04183941 | 0.72 |
| hsa-miR-3678-5p | 1.43 | Up | 8.18E-05 | 0.000533 | 0.85 |
| hsa-miR-7-2* | 1.43 | Up | 2.64E-05 | 0.00024283 | 0.88 |
| hsa-miR-4329 | 1.43 | Up | 0.00022964 | 0.00113406 | 0.81 |
| hsa-miR-3622b-3p | 1.43 | Up | 5.74E-05 | 0.000432 | 0.86 |
| hsa-miR-224* | 1.42 | Up | 2.41E-07 | 1.58E-05 | 0.91 |
| hsa-miR-3685 | 1.42 | Up | 1.93E-05 | 0.00019549 | 0.89 |
| hsa-miR-103 | 1.42 | Up | 0.00332543 | 0.00878759 | 0.75 |
| hsa-miR-663b | 1.42 | Up | 0.00095757 | 0.00332527 | 0.76 |
| hsa-miR-489 | 1.42 | Up | 0.00598818 | 0.01451863 | 0.85 |
| hsa-miR-98 | 1.42 | Up | 0.0200066 | 0.03984786 | 0.70 |
| hsa-miR-29a | 1.41 | Up | 0.01445595 | 0.03018963 | 0.74 |
| hsa-miR-3658 | 1.41 | Up | 0.00904807 | 0.02030341 | 0.81 |
| hsa-miR-606 | 1.41 | Up | 0.00672241 | 0.0160406 | 0.75 |
| hsa-miR-296-5p | 1.41 | Up | 0.00430926 | 0.01084063 | 0.77 |
| hsa-miR-1909 | 1.41 | Up | 0.00377844 | 0.00983374 | 0.77 |
| hsa-miR-3670 | 1.41 | Up | 8.73E-05 | 0.00056065 | 0.88 |
| hsa-miR-519e* | 1.40 | Up | 0.01616554 | 0.03329824 | 0.73 |
| hsa-miR-3910 | 1.40 | Up | 4.34E-05 | 0.00035587 | 0.88 |
| hsa-miR-4254 | 1.40 | Up | 0.00057094 | 0.0022483 | 0.85 |
| hsa-miR-607 | 1.40 | Up | 0.00121963 | 0.00398279 | 0.80 |
| hsa-miR-2117 | 1.39 | Up | 3.14E-06 | 7.89E-05 | 0.89 |
| hsa-miR-1260 | 1.39 | Up | 0.00075804 | 0.00279337 | 0.80 |
| hsa-miR-342-5p | 1.39 | Up | 0.01170527 | 0.02514234 | 0.71 |
| hsa-miR-143 | 1.39 | Up | 0.00013341 | 0.00076189 | 0.85 |
| hsa-miR-660 | 1.39 | Up | 0.01588237 | 0.0328836 | 0.73 |
| hsa-miR-4304 | 1.39 | Up | 8.13E-05 | 0.00053217 | 0.82 |
| hsa-miR-377 | 1.38 | Up | 0.00011221 | 0.0006661 | 0.86 |
| hsa-let-7e* | 1.38 | Up | 1.42E-06 | 4.18E-05 | 0.92 |
| hsa-miR-380* | 1.38 | Up | 0.00383275 | 0.00993667 | 0.80 |
| hsa-miR-506 | 1.38 | Up | 2.97E-06 | 7.62E-05 | 0.90 |
| hsa-miR-3684 | 1.38 | Up | 0.00011054 | 0.00066417 | 0.87 |
| hsa-miR-3176 | 1.38 | Up | 0.00014297 | 0.0007831 | 0.83 |
| hsa-miR-363* | 1.38 | Up | 0.0074069 | 0.01743225 | 0.76 |
| hsa-miR-1247 | 1.38 | Up | 0.00404901 | 0.01033698 | 0.77 |
| hsa-miR-1236 | 1.38 | Up | 0.00014753 | 0.00080441 | 0.85 |
| hsa-miR-30e* | 1.38 | Up | 0.0114782 | 0.02483164 | 0.73 |
| hsa-miR-597 | 1.38 | Up | 0.00082547 | 0.00295162 | 0.83 |
| hsa-miR-18b | 1.37 | Up | 0.0134723 | 0.02833179 | 0.73 |
| hsa-miR-31* | 1.37 | Up | 6.73E-06 | 0.0001106 | 0.91 |
| hsa-miR-640 | 1.37 | Up | 2.28E-05 | 0.00021774 | 0.87 |
| hsa-miR-196a* | 1.37 | Up | 0.0120645 | 0.0257761 | 0.74 |
| hsa-miR-34a* | 1.37 | Up | 8.83E-06 | 0.00012091 | 0.87 |
| hsa-miR-3161 | 1.35 | Up | 9.50E-05 | 0.00058982 | 0.85 |
| hsa-miR-4287 | 1.35 | Up | 5.02E-05 | 0.0003925 | 0.84 |
| hsa-miR-509-3-5p | 1.35 | Up | 0.0017193 | 0.00529861 | 0.77 |
| hsa-miR-590-5p | 1.34 | Up | 0.00329884 | 0.00876096 | 0.76 |
| hsa-miR-346 | 1.34 | Up | 5.42E-05 | 0.000416 | 0.85 |
| hsa-let-7e | 1.34 | Up | 0.0056691 | 0.01388468 | 0.75 |
| hsa-miR-1208 | 1.34 | Up | 0.00051409 | 0.00207879 | 0.83 |
| hsa-miR-379* | 1.33 | Up | 8.48E-06 | 0.00011962 | 0.90 |
| hsa-miR-155 | 1.33 | Up | 0.0177246 | 0.0362617 | 0.76 |
| hsa-miR-615-5p | 1.33 | Up | 0.0004941 | 0.00202512 | 0.83 |
| hsa-miR-636 | 1.33 | Up | 0.0108083 | 0.02363702 | 0.73 |
| hsa-miR-4328 | 1.33 | Up | 0.00230597 | 0.00665032 | 0.75 |
| hsa-miR-122* | 1.33 | Up | 8.04E-05 | 0.00053217 | 0.87 |
| hsa-miR-502-5p | 1.32 | Up | 0.01194382 | 0.02556359 | 0.74 |
| hsa-miR-1976 | 1.32 | Up | 0.00422011 | 0.01068327 | 0.76 |
| hsa-miR-1249 | 1.32 | Up | 0.00393486 | 0.01012598 | 0.74 |
| hsa-miR-764 | 1.32 | Up | 0.00243435 | 0.00694392 | 0.81 |
| hsa-miR-593* | 1.32 | Up | 8.08E-06 | 0.00011962 | 0.92 |
| hsa-miR-153 | 1.31 | Up | 0.00587008 | 0.01428979 | 0.74 |
| hsa-miR-654-5p | 1.31 | Up | 0.00053611 | 0.00214534 | 0.83 |
| hsa-miR-3615 | 1.31 | Up | 0.00773697 | 0.01799815 | 0.75 |
| hsa-miR-486-3p | 1.30 | Up | 0.00224126 | 0.00653926 | 0.79 |
| hsa-miR-624* | 1.30 | Up | 0.00063718 | 0.0024768 | 0.82 |
| hsa-miR-1263 | 1.30 | Up | 0.00213875 | 0.0063012 | 0.83 |
| hsa-miR-499-3p | 1.30 | Up | 0.00074609 | 0.00277485 | 0.82 |
| hsa-miR-758 | 1.30 | Up | 0.00045174 | 0.00187059 | 0.84 |
| hsa-miR-133a | 1.30 | Up | 0.00803448 | 0.01843597 | 0.77 |
| hsa-miR-3177 | 1.29 | Up | 0.0022219 | 0.00649852 | 0.74 |
| hsa-miR-412 | 1.29 | Up | 0.00418089 | 0.01060625 | 0.77 |
| hsa-miR-589* | 1.29 | Up | 0.01524794 | 0.0316543 | 0.74 |
| hsa-miR-3122 | 1.28 | Up | 0.0006176 | 0.00241083 | 0.82 |
| hsa-miR-4326 | 1.28 | Up | 0.00201569 | 0.00608748 | 0.78 |
| hsa-miR-24-1* | 1.28 | Up | 0.00691935 | 0.01640991 | 0.75 |
| hsa-let-7i* | 1.28 | Up | 5.29E-06 | 9.49E-05 | 0.90 |
| hsa-miR-216b | 1.28 | Up | 0.00555682 | 0.01366524 | 0.77 |
| hsa-miR-770-5p | 1.28 | Up | 0.00061821 | 0.00241083 | 0.82 |
| hsa-miR-3128 | 1.28 | Up | 0.00071814 | 0.00270423 | 0.79 |
| hsa-miR-635 | 1.28 | Up | 0.00122874 | 0.0040017 | 0.83 |
| hsa-miR-718 | 1.28 | Up | 0.00162916 | 0.00505963 | 0.77 |
| hsa-miR-1193 | 1.28 | Up | 0.00783985 | 0.01816733 | 0.80 |
| hsa-miR-4297 | 1.28 | Up | 0.0013005 | 0.00419011 | 0.84 |
| hsa-miR-105 | 1.28 | Up | 0.01147228 | 0.02483164 | 0.76 |
| hsa-miR-548v | 1.28 | Up | 2.25E-05 | 0.00021664 | 0.89 |
| hsa-miR-1178 | 1.27 | Up | 3.72E-06 | 8.20E-05 | 0.91 |
| hsa-miR-1203 | 1.27 | Up | 0.00597876 | 0.01451863 | 0.76 |
| hsa-miR-3675-3p | 1.27 | Up | 0.00330081 | 0.00876096 | 0.79 |
| hsa-miR-937 | 1.27 | Up | 0.00327346 | 0.00874616 | 0.80 |
| hsa-miR-200a* | 1.27 | Up | 0.00081584 | 0.00293349 | 0.79 |
| hsa-miR-604 | 1.26 | Up | 0.00252478 | 0.0071585 | 0.76 |
| hsa-miR-212 | 1.26 | Up | 0.00541861 | 0.01337997 | 0.75 |
| hsa-miR-504 | 1.26 | Up | 0.00029087 | 0.0013378 | 0.84 |
| hsa-miR-1200 | 1.26 | Up | 0.00075555 | 0.00279276 | 0.83 |
| hsa-miR-575 | 1.26 | Up | 0.01330206 | 0.02802271 | 0.76 |
| hsa-miR-496 | 1.26 | Up | 0.00481175 | 0.01200447 | 0.84 |
| hsa-miR-1276 | 1.25 | Up | 0.00192002 | 0.00582776 | 0.81 |
| hsa-miR-106a* | 1.25 | Up | 0.00258345 | 0.00729052 | 0.80 |
| hsa-miR-3620 | 1.25 | Up | 0.018008 | 0.03665479 | 0.70 |
| hsa-miR-345 | 1.25 | Up | 0.00884408 | 0.0199946 | 0.76 |
| hsa-miR-3142 | 1.25 | Up | 2.60E-05 | 0.00024133 | 0.86 |
| hsa-miR-3157 | 1.25 | Up | 0.00016625 | 0.0008864 | 0.87 |
| hsa-miR-558 | 1.25 | Up | 0.00139222 | 0.00442644 | 0.77 |
| hsa-miR-3133 | 1.25 | Up | 0.00152486 | 0.00479753 | 0.80 |
| hsa-miR-1289 | 1.24 | Up | 1.38E-07 | 1.58E-05 | 0.93 |
| hsa-miR-1280 | 1.24 | Up | 0.01776228 | 0.0362772 | 0.72 |
| hsa-miR-1273 | 1.24 | Up | 0.00012217 | 0.00071203 | 0.86 |
| hsa-miR-182* | 1.24 | Up | 0.00406968 | 0.0103678 | 0.83 |
| hsa-miR-3666 | 1.24 | Up | 0.00181305 | 0.00557327 | 0.81 |
| hsa-miR-1226 | 1.24 | Up | 0.00230166 | 0.00665032 | 0.81 |
| hsa-miR-103-as | 1.24 | Up | 0.000313 | 0.0014179 | 0.82 |
| hsa-miR-509-5p | 1.24 | Up | 0.01898522 | 0.03819231 | 0.73 |
| hsa-miR-3120 | 1.24 | Up | 0.00429984 | 0.01083956 | 0.77 |
| hsa-miR-3165 | 1.24 | Up | 0.00166411 | 0.00515488 | 0.83 |
| hsa-miR-611 | 1.24 | Up | 0.01059744 | 0.02326031 | 0.73 |
| hsa-miR-1253 | 1.23 | Up | 0.00301597 | 0.00825964 | 0.80 |
| hsa-miR-935 | 1.23 | Up | 0.00033864 | 0.00148385 | 0.80 |
| hsa-miR-1197 | 1.23 | Up | 6.87E-05 | 0.00048424 | 0.88 |
| hsa-miR-3667-3p | 1.23 | Up | 0.00095262 | 0.00331764 | 0.79 |
| hsa-miR-129* | 1.23 | Up | 0.02605575 | 0.04944438 | 0.70 |
| hsa-miR-1227 | 1.23 | Up | 0.00239034 | 0.0068417 | 0.77 |
| hsa-miR-488* | 1.23 | Up | 0.00097656 | 0.00338147 | 0.77 |
| hsa-miR-32 | 1.22 | Up | 0.01807413 | 0.03670237 | 0.79 |
| hsa-miR-4294 | 1.22 | Up | 0.00947737 | 0.02103173 | 0.75 |
| hsa-miR-541* | 1.22 | Up | 0.02122656 | 0.04172594 | 0.74 |
| hsa-miR-650 | 1.22 | Up | 0.00142229 | 0.00449831 | 0.77 |
| hsa-miR-616* | 1.22 | Up | 4.98E-06 | 9.49E-05 | 0.89 |
| hsa-miR-129-3p | 1.22 | Up | 0.02188395 | 0.0428087 | 0.73 |
| hsa-miR-555 | 1.21 | Up | 3.21E-05 | 0.00028056 | 0.87 |
| hsa-miR-3911 | 1.21 | Up | 0.01989015 | 0.03974731 | 0.72 |
| hsa-miR-888* | 1.21 | Up | 0.00520207 | 0.01292473 | 0.78 |
| hsa-miR-187* | 1.20 | Up | 0.02256897 | 0.04374312 | 0.73 |
| hsa-miR-582-5p | 1.20 | Up | 0.00139726 | 0.00443077 | 0.85 |
| hsa-miR-548z | 1.20 | Up | 0.00524521 | 0.01297839 | 0.85 |
| hsa-miR-605 | 1.20 | Up | 0.00320735 | 0.00858858 | 0.81 |
| hsa-miR-634 | 1.20 | Up | 0.00106001 | 0.00357791 | 0.83 |
| hsa-miR-625* | 1.20 | Up | 0.01935249 | 0.03886625 | 0.71 |
| hsa-miR-551a | 1.19 | Up | 5.24E-06 | 9.49E-05 | 0.95 |
| hsa-miR-525-5p | 1.19 | Up | 0.01763066 | 0.03613085 | 0.72 |
| hsa-miR-877* | 1.19 | Up | 0.00354531 | 0.00925879 | 0.77 |
| hsa-miR-513a-3p | 1.19 | Up | 0.00263483 | 0.00740087 | 0.81 |
| hsa-miR-19b-1* | 1.19 | Up | 0.00831774 | 0.01898272 | 0.80 |
| hsa-miR-2113 | 1.18 | Up | 0.00748975 | 0.01752457 | 0.77 |
| hsa-miR-767-3p | 1.18 | Up | 0.0008032 | 0.00289777 | 0.81 |
| hsa-miR-222* | 1.18 | Up | 0.01647629 | 0.03388042 | 0.78 |
| hsa-miR-515-5p | 1.18 | Up | 0.00655167 | 0.01566422 | 0.77 |
| hsa-miR-4268 | 1.18 | Up | 0.00620271 | 0.01494854 | 0.79 |
| hsa-miR-551b | 1.18 | Up | 3.81E-06 | 8.20E-05 | 0.91 |
| hsa-miR-1229 | 1.18 | Up | 0.00269211 | 0.00749189 | 0.74 |
| hsa-miR-1237 | 1.17 | Up | 0.00110451 | 0.0036868 | 0.80 |
| hsa-miR-3130-5p | 1.17 | Up | 0.0126749 | 0.02698455 | 0.72 |
| hsa-miR-3074 | 1.17 | Up | 8.12E-05 | 0.00053217 | 0.90 |
| hsa-miR-3166 | 1.17 | Up | 0.02350361 | 0.04523215 | 0.76 |
| hsa-miR-2909 | 1.17 | Up | 0.00644853 | 0.01544828 | 0.75 |
| hsa-miR-661 | 1.17 | Up | 0.00781737 | 0.01815015 | 0.77 |
| hsa-miR-3692 | 1.16 | Up | 0.00939823 | 0.02089459 | 0.74 |
| hsa-miR-589 | 1.16 | Up | 0.00160503 | 0.00499759 | 0.83 |
| hsa-miR-3194 | 1.16 | Up | 0.00448099 | 0.01122576 | 0.70 |
| hsa-miR-3679-3p | 1.16 | Up | 0.00903323 | 0.02030341 | 0.78 |
| hsa-miR-548p | 1.16 | Up | 0.00127827 | 0.0041516 | 0.81 |
| hsa-miR-4290 | 1.15 | Up | 0.02353573 | 0.04523215 | 0.75 |
| hsa-miR-548s | 1.15 | Up | 0.01526237 | 0.0316543 | 0.76 |
| hsa-miR-4276 | 1.15 | Up | 0.02104828 | 0.04151093 | 0.76 |
| hsa-miR-3922 | 1.15 | Up | 0.00922767 | 0.0205533 | 0.78 |
| hsa-miR-518b | 1.15 | Up | 0.00425743 | 0.01075514 | 0.77 |
| hsa-miR-1204 | 1.14 | Up | 0.00687916 | 0.01638218 | 0.75 |
| hsa-miR-514b-3p | 1.14 | Up | 0.00839892 | 0.01913176 | 0.77 |
| hsa-miR-18b* | 1.14 | Up | 0.01809229 | 0.03670237 | 0.75 |
| hsa-miR-675 | 1.14 | Up | 0.01223384 | 0.02609165 | 0.75 |
| hsa-miR-1538 | 1.14 | Up | 0.01875784 | 0.03792482 | 0.78 |
| hsa-miR-3921 | 1.13 | Up | 0.00237342 | 0.00680946 | 0.77 |
| hsa-miR-1225-3p | 1.13 | Up | 0.00218869 | 0.00641698 | 0.74 |
| hsa-miR-15a* | 1.12 | Up | 0.00017044 | 0.00089686 | 0.89 |
| hsa-miR-570 | 1.11 | Up | 0.01279693 | 0.0271963 | 0.75 |
| hsa-miR-523 | 1.10 | Up | 0.02151876 | 0.04216278 | 0.69 |
| hsa-miR-300 | 1.09 | Up | 0.02097966 | 0.04144342 | 0.73 |
| hsa-miR-491-3p | 1.09 | Up | 0.01282443 | 0.02720675 | 0.78 |
| hsa-miR-432* | 1.08 | Up | 0.00793318 | 0.01831317 | 0.80 |
| hsa-miR-185* | 1.07 | Up | 0.01054822 | 0.02322777 | 0.77 |
| hsa-miR-603 | 1.05 | Up | 0.02634142 | 0.04990789 | 0.68 |
| hsa-miR-181d | 5.52 | Down | 1.44E-05 | 0.0001644 | 0.05 |
| hsa-miR-3653 | 4.28 | Down | 0.0021129 | 0.00627106 | 0.21 |
| hsa-miR-206 | 2.82 | Down | 3.20E-07 | 1.84E-05 | 0.07 |
| hsa-miR-3201 | 2.69 | Down | 0.00698563 | 0.01650525 | 0.16 |
| hsa-miR-142-5p | 2.60 | Down | 0.00047451 | 0.00195149 | 0.19 |
| hsa-miR-181b | 2.56 | Down | 0.00077746 | 0.00285069 | 0.17 |
| hsa-miR-194 | 2.49 | Down | 2.20E-07 | 1.58E-05 | 0.06 |
| hsa-miR-339-5p | 2.43 | Down | 1.61E-07 | 1.58E-05 | 0.03 |
| hsa-miR-26a | 2.36 | Down | 0.00210366 | 0.00625904 | 0.20 |
| hsa-miR-1268 | 2.33 | Down | 0.01112032 | 0.02418769 | 0.22 |
| hsa-miR-595 | 2.31 | Down | 0.0003578 | 0.00154829 | 0.17 |
| hsa-miR-151-3p | 2.20 | Down | 6.92E-08 | 1.58E-05 | 0.04 |
| hsa-miR-4271 | 2.19 | Down | 1.24E-05 | 0.00015531 | 0.10 |
| hsa-miR-1307 | 2.16 | Down | 0.0021011 | 0.00625904 | 0.25 |
| hsa-miR-222 | 2.16 | Down | 0.00025831 | 0.00122543 | 0.14 |
| hsa-miR-30c | 2.15 | Down | 5.78E-05 | 0.00043226 | 0.13 |
| hsa-miR-620 | 2.11 | Down | 8.40E-07 | 3.35E-05 | 0.07 |
| hsa-miR-623 | 2.09 | Down | 0.00031236 | 0.0014179 | 0.12 |
| hsa-miR-30b | 2.04 | Down | 0.00033576 | 0.00148204 | 0.15 |
| hsa-miR-3689b* | 2.04 | Down | 4.11E-07 | 1.98E-05 | 0.03 |
| hsa-miR-186 | 2.03 | Down | 0.00975693 | 0.02157267 | 0.29 |
| hsa-miR-191 | 2.00 | Down | 4.73E-07 | 2.11E-05 | 0.06 |
| hsa-miR-30d | 1.96 | Down | 2.51E-05 | 0.00023632 | 0.11 |
| hsa-miR-3147 | 1.95 | Down | 1.73E-07 | 1.58E-05 | 0.08 |
| hsa-miR-1228* | 1.92 | Down | 0.00111439 | 0.00370949 | 0.17 |
| hsa-miR-564 | 1.90 | Down | 0.00158197 | 0.00493853 | 0.24 |
| hsa-miR-3129 | 1.89 | Down | 8.46E-06 | 0.00011962 | 0.09 |
| hsa-miR-1 | 1.88 | Down | 1.41E-05 | 0.0001644 | 0.11 |
| hsa-miR-1262 | 1.87 | Down | 3.70E-07 | 1.88E-05 | 0.06 |
| hsa-miR-140-3p | 1.86 | Down | 5.88E-06 | 0.00010117 | 0.06 |
| hsa-miR-1244 | 1.86 | Down | 1.10E-06 | 3.69E-05 | 0.07 |
| hsa-miR-1246 | 1.85 | Down | 0.01406696 | 0.02947945 | 0.25 |
| hsa-miR-196a | 1.84 | Down | 3.65E-06 | 8.20E-05 | 0.10 |
| hsa-miR-378 | 1.78 | Down | 0.0072537 | 0.01710511 | 0.24 |
| hsa-miR-320a | 1.78 | Down | 0.00055638 | 0.00220149 | 0.19 |
| hsa-miR-1180 | 1.77 | Down | 8.54E-06 | 0.00011962 | 0.09 |
| hsa-miR-432 | 1.75 | Down | 7.49E-06 | 0.00011284 | 0.07 |
| hsa-miR-425 | 1.75 | Down | 3.10E-05 | 0.00027681 | 0.10 |
| hsa-miR-532-3p | 1.74 | Down | 0.00020689 | 0.00104943 | 0.20 |
| hsa-miR-198 | 1.73 | Down | 3.16E-07 | 1.84E-05 | 0.06 |
| hsa-miR-1306 | 1.73 | Down | 0.00068621 | 0.00260026 | 0.16 |
| hsa-miR-616 | 1.71 | Down | 0.00028768 | 0.00133328 | 0.17 |
| hsa-miR-1321 | 1.71 | Down | 3.15E-05 | 0.00027927 | 0.06 |
| hsa-miR-670 | 1.71 | Down | 0.00016704 | 0.00088673 | 0.14 |
| hsa-miR-3145 | 1.71 | Down | 1.44E-05 | 0.0001644 | 0.08 |
| hsa-miR-3686 | 1.71 | Down | 0.0031414 | 0.00850616 | 0.16 |
| hsa-miR-3662 | 1.69 | Down | 1.26E-06 | 3.90E-05 | 0.07 |
| hsa-miR-139-3p | 1.68 | Down | 0.00078445 | 0.00285577 | 0.20 |
| hsa-miR-182 | 1.68 | Down | 0.00188209 | 0.00572706 | 0.22 |
| hsa-miR-25 | 1.67 | Down | 9.05E-05 | 0.00057245 | 0.13 |
| hsa-miR-190b | 1.66 | Down | 9.96E-06 | 0.0001334 | 0.08 |
| hsa-miR-551b* | 1.66 | Down | 4.70E-05 | 0.00037291 | 0.15 |
| hsa-miR-3668 | 1.65 | Down | 1.05E-05 | 0.00013951 | 0.12 |
| hsa-miR-199a-5p | 1.65 | Down | 3.52E-05 | 0.00030106 | 0.10 |
| hsa-miR-33b* | 1.64 | Down | 0.00612418 | 0.01478886 | 0.20 |
| hsa-miR-3131 | 1.64 | Down | 5.27E-06 | 9.49E-05 | 0.09 |
| hsa-miR-3663-5p | 1.64 | Down | 0.00396805 | 0.01015182 | 0.21 |
| hsa-miR-92a | 1.62 | Down | 0.00066786 | 0.00253869 | 0.23 |
| hsa-miR-920 | 1.62 | Down | 4.01E-05 | 0.00033517 | 0.13 |
| hsa-miR-576-3p | 1.62 | Down | 1.29E-07 | 1.58E-05 | 0.04 |
| hsa-miR-4316 | 1.62 | Down | 0.00104013 | 0.00352065 | 0.23 |
| hsa-miR-548f | 1.62 | Down | 0.00025241 | 0.00120697 | 0.06 |
| hsa-miR-93* | 1.61 | Down | 7.22E-06 | 0.00011149 | 0.10 |
| hsa-miR-342-3p | 1.60 | Down | 0.00013096 | 0.00075338 | 0.14 |
| hsa-miR-548n | 1.60 | Down | 1.41E-05 | 0.0001644 | 0.13 |
| hsa-miR-190 | 1.59 | Down | 2.50E-07 | 1.58E-05 | 0.03 |
| hsa-miR-137 | 1.59 | Down | 6.93E-06 | 0.0001106 | 0.13 |
| hsa-miR-3611 | 1.58 | Down | 0.00094964 | 0.00331686 | 0.19 |
| hsa-miR-3152 | 1.56 | Down | 0.00022961 | 0.00113406 | 0.18 |
| hsa-miR-423-5p | 1.55 | Down | 0.00099892 | 0.00341958 | 0.20 |
| hsa-miR-1293 | 1.55 | Down | 0.00010743 | 0.00065049 | 0.12 |
| hsa-miR-3713 | 1.55 | Down | 5.15E-08 | 1.58E-05 | 0.03 |
| hsa-miR-922 | 1.55 | Down | 0.00081797 | 0.00293349 | 0.26 |
| hsa-miR-3619 | 1.55 | Down | 0.00134874 | 0.00431097 | 0.15 |
| hsa-miR-187 | 1.54 | Down | 4.34E-05 | 0.00035587 | 0.13 |
| hsa-miR-494 | 1.54 | Down | 0.00352028 | 0.00922689 | 0.22 |
| hsa-miR-99b | 1.54 | Down | 0.00129262 | 0.00417588 | 0.20 |
| hsa-miR-1265 | 1.54 | Down | 7.52E-05 | 0.00051502 | 0.14 |
| hsa-miR-3617 | 1.53 | Down | 2.20E-07 | 1.58E-05 | 0.07 |
| hsa-miR-3647-3p | 1.53 | Down | 3.63E-06 | 8.20E-05 | 0.06 |
| hsa-miR-548d-5p | 1.53 | Down | 0.00187127 | 0.00570856 | 0.13 |
| hsa-miR-3189 | 1.53 | Down | 1.36E-05 | 0.0001644 | 0.10 |
| hsa-miR-32* | 1.52 | Down | 7.10E-06 | 0.0001111 | 0.10 |
| hsa-miR-545* | 1.51 | Down | 2.24E-06 | 5.98E-05 | 0.07 |
| hsa-miR-3616-3p | 1.51 | Down | 0.0117938 | 0.02528743 | 0.31 |
| hsa-miR-3917 | 1.51 | Down | 0.00346142 | 0.00910701 | 0.20 |
| hsa-miR-548m | 1.51 | Down | 1.46E-05 | 0.0001644 | 0.04 |
| hsa-miR-3115 | 1.51 | Down | 1.08E-05 | 0.00014191 | 0.09 |
| hsa-miR-383 | 1.51 | Down | 1.91E-05 | 0.00019481 | 0.11 |
| hsa-miR-486-5p | 1.51 | Down | 0.02519371 | 0.04803548 | 0.31 |
| hsa-miR-3192 | 1.50 | Down | 1.47E-05 | 0.0001644 | 0.09 |
| hsa-miR-2052 | 1.50 | Down | 4.72E-06 | 9.47E-05 | 0.10 |
| hsa-miR-449b | 1.50 | Down | 1.42E-05 | 0.0001644 | 0.13 |
| hsa-miR-29a* | 1.50 | Down | 2.07E-05 | 0.00020302 | 0.16 |
| hsa-miR-2278 | 1.49 | Down | 1.49E-05 | 0.00016489 | 0.04 |
| hsa-miR-3936 | 1.49 | Down | 8.81E-05 | 0.00056169 | 0.15 |
| hsa-miR-590-3p | 1.49 | Down | 0.0002386 | 0.00117066 | 0.17 |
| hsa-miR-1257 | 1.48 | Down | 7.73E-05 | 0.0005235 | 0.13 |
| hsa-miR-3923 | 1.48 | Down | 0.00012226 | 0.00071203 | 0.13 |
| hsa-miR-4278 | 1.48 | Down | 4.41E-05 | 0.00035625 | 0.16 |
| hsa-miR-3140 | 1.48 | Down | 9.12E-05 | 0.00057245 | 0.15 |
| hsa-miR-34c-5p | 1.47 | Down | 7.49E-06 | 0.00011284 | 0.13 |
| hsa-miR-1258 | 1.47 | Down | 0.00028968 | 0.0013374 | 0.16 |
| hsa-miR-3673 | 1.47 | Down | 1.61E-05 | 0.00017582 | 0.10 |
| hsa-miR-520d-3p | 1.47 | Down | 0.00012232 | 0.00071203 | 0.16 |
| hsa-miR-1252 | 1.46 | Down | 0.00020727 | 0.00104943 | 0.19 |
| hsa-miR-3689a-3p | 1.45 | Down | 3.19E-05 | 0.00028035 | 0.12 |
| hsa-miR-147 | 1.44 | Down | 0.00034679 | 0.00151406 | 0.18 |
| hsa-miR-30a | 1.44 | Down | 0.00120937 | 0.00396003 | 0.18 |
| hsa-miR-3162 | 1.44 | Down | 0.0211048 | 0.04155438 | 0.28 |
| hsa-miR-941 | 1.44 | Down | 0.0007135 | 0.00269521 | 0.15 |
| hsa-miR-3179 | 1.44 | Down | 0.00014044 | 0.00077628 | 0.16 |
| hsa-miR-3650 | 1.43 | Down | 0.00026806 | 0.00126179 | 0.16 |
| hsa-miR-3622a-5p | 1.43 | Down | 7.32E-05 | 0.00050695 | 0.14 |
| hsa-miR-374c | 1.43 | Down | 0.01162792 | 0.02506555 | 0.24 |
| hsa-miR-19b-2* | 1.42 | Down | 0.0002514 | 0.00120694 | 0.19 |
| hsa-miR-3149 | 1.42 | Down | 2.07E-05 | 0.00020302 | 0.10 |
| hsa-miR-3609 | 1.42 | Down | 0.00039267 | 0.00166608 | 0.16 |
| hsa-miR-141 | 1.42 | Down | 4.62E-06 | 9.43E-05 | 0.10 |
| hsa-miR-3669 | 1.42 | Down | 1.39E-05 | 0.0001644 | 0.12 |
| hsa-miR-548b-5p | 1.41 | Down | 4.55E-05 | 0.00036567 | 0.10 |
| hsa-miR-522 | 1.41 | Down | 0.00027588 | 0.00129352 | 0.17 |
| hsa-miR-885-3p | 1.40 | Down | 0.00132389 | 0.0042497 | 0.19 |
| hsa-miR-556-5p | 1.40 | Down | 0.00033828 | 0.00148385 | 0.19 |
| hsa-miR-500a | 1.40 | Down | 0.00967591 | 0.02143284 | 0.20 |
| hsa-miR-3927 | 1.40 | Down | 1.95E-05 | 0.00019563 | 0.10 |
| hsa-miR-3186-3p | 1.40 | Down | 8.05E-05 | 0.00053217 | 0.12 |
| hsa-miR-1182 | 1.39 | Down | 0.00024396 | 0.00118535 | 0.15 |
| hsa-miR-580 | 1.39 | Down | 0.00018005 | 0.00093923 | 0.11 |
| hsa-miR-649 | 1.39 | Down | 0.00207717 | 0.00624186 | 0.15 |
| hsa-miR-3678-3p | 1.38 | Down | 0.00024622 | 0.00119154 | 0.17 |
| hsa-miR-33a | 1.38 | Down | 1.97E-05 | 0.00019641 | 0.13 |
| hsa-miR-4257 | 1.38 | Down | 4.70E-05 | 0.00037291 | 0.14 |
| hsa-miR-374a* | 1.38 | Down | 0.00022375 | 0.00111415 | 0.23 |
| hsa-miR-325 | 1.38 | Down | 1.44E-05 | 0.0001644 | 0.11 |
| hsa-miR-105* | 1.38 | Down | 4.39E-05 | 0.00035625 | 0.16 |
| hsa-miR-92a-2* | 1.38 | Down | 5.96E-07 | 2.57E-05 | 0.03 |
| hsa-miR-548t | 1.37 | Down | 0.00018379 | 0.00094645 | 0.10 |
| hsa-miR-515-3p | 1.37 | Down | 0.00031044 | 0.00141697 | 0.17 |
| hsa-miR-601 | 1.37 | Down | 0.00013129 | 0.00075338 | 0.17 |
| hsa-miR-876-3p | 1.37 | Down | 5.65E-05 | 0.00042787 | 0.16 |
| hsa-miR-3174 | 1.36 | Down | 0.00020868 | 0.00105214 | 0.17 |
| hsa-miR-1290 | 1.36 | Down | 0.00087872 | 0.00311632 | 0.16 |
| hsa-miR-544b | 1.36 | Down | 0.00013866 | 0.00077527 | 0.14 |
| hsa-miR-4311 | 1.36 | Down | 0.0004999 | 0.00203208 | 0.17 |
| hsa-miR-1248 | 1.36 | Down | 0.00041579 | 0.00174572 | 0.18 |
| hsa-miR-1205 | 1.35 | Down | 5.87E-06 | 0.00010117 | 0.08 |
| hsa-miR-1294 | 1.35 | Down | 0.01761252 | 0.03613085 | 0.23 |
| hsa-miR-3915 | 1.35 | Down | 0.00090686 | 0.00318589 | 0.16 |
| hsa-miR-3137 | 1.35 | Down | 0.00690288 | 0.01640626 | 0.25 |
| hsa-miR-27b* | 1.35 | Down | 0.00058584 | 0.00229946 | 0.19 |
| hsa-miR-4302 | 1.34 | Down | 0.00016038 | 0.00086169 | 0.10 |
| hsa-miR-548l | 1.34 | Down | 0.00035848 | 0.00154829 | 0.14 |
| hsa-miR-297 | 1.34 | Down | 0.00042563 | 0.00178086 | 0.16 |
| hsa-miR-3136 | 1.33 | Down | 0.00011134 | 0.00066417 | 0.15 |
| hsa-miR-548a-5p | 1.33 | Down | 4.91E-05 | 0.00038636 | 0.15 |
| hsa-miR-382 | 1.33 | Down | 3.46E-05 | 0.00030017 | 0.09 |
| hsa-miR-4273 | 1.33 | Down | 0.0028073 | 0.00777654 | 0.18 |
| hsa-miR-573 | 1.33 | Down | 0.00266886 | 0.0074565 | 0.22 |
| hsa-miR-592 | 1.33 | Down | 5.38E-05 | 0.00041592 | 0.15 |
| hsa-miR-373 | 1.33 | Down | 0.00014856 | 0.00080636 | 0.18 |
| hsa-miR-3929 | 1.33 | Down | 0.00914633 | 0.02040986 | 0.26 |
| hsa-miR-2110 | 1.33 | Down | 0.00098288 | 0.0033936 | 0.18 |
| hsa-miR-18a* | 1.32 | Down | 0.00033279 | 0.00147975 | 0.11 |
| hsa-miR-33b | 1.32 | Down | 0.00045916 | 0.00189481 | 0.22 |
| hsa-miR-30a* | 1.32 | Down | 0.00043353 | 0.00180762 | 0.20 |
| hsa-miR-1277 | 1.32 | Down | 5.23E-05 | 0.00040696 | 0.15 |
| hsa-miR-125b-1* | 1.32 | Down | 0.00153336 | 0.00481173 | 0.19 |
| hsa-miR-1206 | 1.32 | Down | 0.0007494 | 0.00277854 | 0.16 |
| hsa-miR-4255 | 1.32 | Down | 0.00301518 | 0.00825964 | 0.21 |
| hsa-miR-4307 | 1.32 | Down | 0.00025421 | 0.00121076 | 0.19 |
| hsa-miR-3614-3p | 1.32 | Down | 0.00015472 | 0.00083606 | 0.19 |
| hsa-miR-3169 | 1.31 | Down | 0.00020313 | 0.00103718 | 0.15 |
| hsa-miR-3613-5p | 1.31 | Down | 0.00014277 | 0.0007831 | 0.17 |
| hsa-miR-1283 | 1.31 | Down | 0.00021651 | 0.00108705 | 0.04 |
| hsa-miR-519c-3p | 1.31 | Down | 0.00011132 | 0.00066417 | 0.15 |
| hsa-miR-578 | 1.31 | Down | 6.86E-05 | 0.00048424 | 0.16 |
| hsa-miR-3924 | 1.31 | Down | 0.00210333 | 0.00625904 | 0.23 |
| hsa-miR-552 | 1.31 | Down | 0.00227569 | 0.00660797 | 0.18 |
| hsa-miR-30b* | 1.31 | Down | 0.00049596 | 0.00202586 | 0.19 |
| hsa-miR-367* | 1.31 | Down | 0.0015514 | 0.00485568 | 0.22 |
| hsa-miR-3150 | 1.30 | Down | 0.00038703 | 0.00164797 | 0.20 |
| hsa-miR-450b-5p | 1.30 | Down | 0.00317694 | 0.00854511 | 0.22 |
| hsa-miR-586 | 1.30 | Down | 0.00018255 | 0.00094645 | 0.20 |
| hsa-miR-676 | 1.30 | Down | 0.02038721 | 0.04040558 | 0.26 |
| hsa-miR-3606 | 1.30 | Down | 6.32E-05 | 0.00045575 | 0.14 |
| hsa-miR-520c-3p | 1.30 | Down | 0.0007461 | 0.00277485 | 0.21 |
| hsa-miR-1912 | 1.29 | Down | 0.0240788 | 0.04620216 | 0.29 |
| hsa-miR-548g | 1.29 | Down | 0.00050085 | 0.00203208 | 0.17 |
| hsa-miR-520e | 1.29 | Down | 1.90E-05 | 0.00019481 | 0.11 |
| hsa-miR-3622b-5p | 1.29 | Down | 0.00013889 | 0.00077527 | 0.10 |
| hsa-miR-3146 | 1.29 | Down | 0.00030442 | 0.00139479 | 0.15 |
| hsa-miR-2115* | 1.29 | Down | 0.0002778 | 0.00129747 | 0.19 |
| hsa-miR-4259 | 1.28 | Down | 0.00089737 | 0.0031618 | 0.19 |
| hsa-miR-1273d | 1.28 | Down | 0.00747884 | 0.01752457 | 0.23 |
| hsa-miR-3188 | 1.28 | Down | 0.00907277 | 0.02032097 | 0.24 |
| hsa-miR-3916 | 1.28 | Down | 0.00138293 | 0.00440855 | 0.17 |
| hsa-miR-767-5p | 1.28 | Down | 0.00024109 | 0.00117615 | 0.15 |
| hsa-miR-557 | 1.27 | Down | 0.00078322 | 0.00285577 | 0.18 |
| hsa-miR-520a-3p | 1.27 | Down | 0.00635239 | 0.01527869 | 0.27 |
| hsa-miR-626 | 1.27 | Down | 0.00089669 | 0.0031618 | 0.22 |
| hsa-miR-449a | 1.27 | Down | 6.78E-05 | 0.00048373 | 0.13 |
| hsa-miR-517c | 1.27 | Down | 0.0003206 | 0.00143616 | 0.19 |
| hsa-miR-100* | 1.27 | Down | 6.66E-05 | 0.00047782 | 0.14 |
| hsa-miR-1322 | 1.27 | Down | 0.00757251 | 0.01768386 | 0.23 |
| hsa-miR-553 | 1.27 | Down | 9.12E-05 | 0.00057245 | 0.09 |
| hsa-miR-1261 | 1.27 | Down | 0.00116321 | 0.00385074 | 0.20 |
| hsa-miR-3689a-5p | 1.26 | Down | 0.00243757 | 0.00694392 | 0.32 |
| hsa-miR-302c | 1.26 | Down | 0.00796054 | 0.0183412 | 0.25 |
| hsa-miR-3675-5p | 1.26 | Down | 0.00693165 | 0.01640991 | 0.22 |
| hsa-miR-3164 | 1.26 | Down | 0.00566626 | 0.01388468 | 0.32 |
| hsa-miR-520f | 1.26 | Down | 0.0006527 | 0.00250771 | 0.16 |
| hsa-miR-3134 | 1.25 | Down | 7.71E-05 | 0.0005235 | 0.10 |
| hsa-miR-648 | 1.25 | Down | 0.0026732 | 0.0074565 | 0.21 |
| hsa-miR-548j | 1.25 | Down | 0.00034834 | 0.00151535 | 0.21 |
| hsa-miR-10b* | 1.25 | Down | 0.00044488 | 0.00184855 | 0.22 |
| hsa-miR-302a | 1.25 | Down | 0.00031485 | 0.00142097 | 0.19 |
| hsa-miR-34b* | 1.25 | Down | 0.00329394 | 0.00876096 | 0.24 |
| hsa-miR-2116 | 1.25 | Down | 0.00335431 | 0.00884451 | 0.25 |
| hsa-miR-571 | 1.25 | Down | 0.00099247 | 0.00340981 | 0.13 |
| hsa-miR-3190 | 1.24 | Down | 0.01089866 | 0.02374843 | 0.22 |
| hsa-miR-3908 | 1.24 | Down | 0.00128166 | 0.0041516 | 0.21 |
| hsa-miR-548y | 1.24 | Down | 0.01996259 | 0.03982603 | 0.33 |
| hsa-miR-1323 | 1.24 | Down | 7.93E-05 | 0.00053077 | 0.14 |
| hsa-miR-548i | 1.24 | Down | 0.00216232 | 0.00635512 | 0.23 |
| hsa-miR-518c | 1.24 | Down | 0.02257943 | 0.04374312 | 0.26 |
| hsa-miR-3647-5p | 1.24 | Down | 0.00904238 | 0.02030341 | 0.23 |
| hsa-miR-1254 | 1.24 | Down | 0.00019461 | 0.00099789 | 0.15 |
| hsa-miR-510 | 1.24 | Down | 0.01056334 | 0.02322777 | 0.24 |
| hsa-miR-3153 | 1.24 | Down | 2.53E-05 | 0.00023632 | 0.14 |
| hsa-miR-3937 | 1.24 | Down | 0.00354984 | 0.00925879 | 0.22 |
| hsa-miR-518e* | 1.24 | Down | 1.03E-07 | 1.58E-05 | 0.05 |
| hsa-miR-4289 | 1.23 | Down | 0.00119843 | 0.00393491 | 0.20 |
| hsa-miR-448 | 1.23 | Down | 0.00013118 | 0.00075338 | 0.15 |
| hsa-miR-1267 | 1.23 | Down | 0.00307188 | 0.0083747 | 0.23 |
| hsa-miR-450a | 1.23 | Down | 0.00037728 | 0.00161786 | 0.20 |
| hsa-miR-526b* | 1.23 | Down | 0.00013945 | 0.00077527 | 0.19 |
| hsa-miR-27a* | 1.23 | Down | 1.20E-05 | 0.00015213 | 0.08 |
| hsa-miR-28-3p | 1.23 | Down | 0.00028316 | 0.0013174 | 0.17 |
| hsa-miR-520g | 1.23 | Down | 0.00032215 | 0.00143776 | 0.17 |
| hsa-miR-520b | 1.23 | Down | 0.00074223 | 0.00277485 | 0.18 |
| hsa-miR-200a | 1.22 | Down | 0.0031554 | 0.00850616 | 0.24 |
| hsa-miR-639 | 1.22 | Down | 0.01481724 | 0.03086021 | 0.27 |
| hsa-miR-3171 | 1.22 | Down | 0.00018308 | 0.00094645 | 0.16 |
| hsa-miR-765 | 1.22 | Down | 0.01482827 | 0.03086021 | 0.27 |
| hsa-miR-422a | 1.22 | Down | 0.01327209 | 0.02802271 | 0.28 |
| hsa-miR-122 | 1.21 | Down | 0.00101382 | 0.00346078 | 0.21 |
| hsa-miR-518c* | 1.21 | Down | 0.00571858 | 0.01394916 | 0.23 |
| hsa-miR-3934 | 1.21 | Down | 0.02446087 | 0.04686064 | 0.31 |
| hsa-miR-944 | 1.21 | Down | 0.00183546 | 0.00562781 | 0.24 |
| hsa-miR-644 | 1.21 | Down | 0.0029252 | 0.00808455 | 0.22 |
| hsa-miR-1250 | 1.21 | Down | 0.02201259 | 0.04299055 | 0.27 |
| hsa-miR-1243 | 1.21 | Down | 0.00054994 | 0.00218707 | 0.20 |
| hsa-miR-1179 | 1.20 | Down | 0.00264106 | 0.00740111 | 0.28 |
| hsa-miR-651 | 1.20 | Down | 0.0039464 | 0.01012598 | 0.26 |
| hsa-miR-19a* | 1.20 | Down | 0.0087544 | 0.01982905 | 0.25 |
| hsa-miR-135a | 1.20 | Down | 0.01887018 | 0.03802435 | 0.26 |
| hsa-miR-3945 | 1.20 | Down | 0.01858252 | 0.03763351 | 0.23 |
| hsa-miR-568 | 1.20 | Down | 0.00898431 | 0.02027359 | 0.25 |
| hsa-miR-425* | 1.20 | Down | 0.00772685 | 0.01799815 | 0.25 |
| hsa-miR-3148 | 1.20 | Down | 0.00230691 | 0.00665032 | 0.29 |
| hsa-miR-149* | 1.20 | Down | 0.02503516 | 0.04788471 | 0.25 |
| hsa-miR-205* | 1.19 | Down | 0.02341984 | 0.04515346 | 0.26 |
| hsa-miR-1299 | 1.19 | Down | 0.00079575 | 0.00287953 | 0.20 |
| hsa-miR-3925 | 1.19 | Down | 0.00212861 | 0.00629436 | 0.24 |
| hsa-miR-302b* | 1.19 | Down | 0.0031506 | 0.00850616 | 0.25 |
| hsa-miR-302a* | 1.19 | Down | 0.0027267 | 0.00757067 | 0.25 |
| hsa-miR-136 | 1.19 | Down | 0.00296919 | 0.00816865 | 0.16 |
| hsa-miR-184 | 1.19 | Down | 0.00394955 | 0.01012598 | 0.13 |
| hsa-miR-135b | 1.19 | Down | 0.01425194 | 0.02981526 | 0.26 |
| hsa-miR-561 | 1.19 | Down | 0.00305378 | 0.00834423 | 0.27 |
| hsa-miR-760 | 1.19 | Down | 0.00798169 | 0.01835484 | 0.31 |
| hsa-miR-499-5p | 1.18 | Down | 9.30E-05 | 0.00058044 | 0.15 |
| hsa-miR-3150b | 1.18 | Down | 0.00087355 | 0.0031143 | 0.20 |
| hsa-miR-203 | 1.18 | Down | 0.0021312 | 0.00629436 | 0.25 |
| hsa-miR-924 | 1.18 | Down | 0.00107518 | 0.00360888 | 0.24 |
| hsa-miR-1245 | 1.18 | Down | 0.001074 | 0.00360888 | 0.22 |
| hsa-miR-374b* | 1.18 | Down | 0.01144973 | 0.02483164 | 0.22 |
| hsa-miR-132* | 1.17 | Down | 0.00170384 | 0.00526442 | 0.23 |
| hsa-miR-548w | 1.17 | Down | 0.01083261 | 0.02364727 | 0.33 |
| hsa-miR-614 | 1.17 | Down | 0.01152444 | 0.02488702 | 0.27 |
| hsa-miR-3912 | 1.17 | Down | 0.01073666 | 0.02352305 | 0.24 |
| hsa-miR-9 | 1.17 | Down | 5.84E-05 | 0.00043436 | 0.10 |
| hsa-miR-519b-3p | 1.17 | Down | 0.0025776 | 0.00729052 | 0.22 |
| hsa-miR-377* | 1.17 | Down | 3.49E-05 | 0.00030071 | 0.11 |
| hsa-miR-3928 | 1.17 | Down | 0.00251155 | 0.00713779 | 0.25 |
| hsa-miR-367 | 1.16 | Down | 0.00037023 | 0.0015933 | 0.21 |
| hsa-miR-3657 | 1.16 | Down | 0.00184456 | 0.00564137 | 0.23 |
| hsa-miR-3167 | 1.16 | Down | 0.00053467 | 0.00214534 | 0.21 |
| hsa-miR-3687 | 1.15 | Down | 0.01329996 | 0.02802271 | 0.31 |
| hsa-miR-524-3p | 1.15 | Down | 0.01975692 | 0.03954665 | 0.25 |
| hsa-miR-488 | 1.15 | Down | 0.00522972 | 0.0129667 | 0.22 |
| hsa-miR-1282 | 1.15 | Down | 0.01960596 | 0.0393098 | 0.36 |
| hsa-miR-1255a | 1.15 | Down | 0.02089035 | 0.04133477 | 0.28 |
| hsa-miR-1287 | 1.15 | Down | 0.00913992 | 0.02040986 | 0.23 |
| hsa-miR-581 | 1.15 | Down | 0.00152389 | 0.00479753 | 0.22 |
| hsa-miR-302b | 1.15 | Down | 0.00207347 | 0.00624186 | 0.22 |
| hsa-miR-550a | 1.14 | Down | 0.00571055 | 0.01394916 | 0.22 |
| hsa-miR-299-3p | 1.13 | Down | 0.02326612 | 0.04492897 | 0.27 |
| hsa-miR-508-3p | 1.13 | Down | 0.0257862 | 0.04901004 | 0.26 |
| hsa-miR-873 | 1.12 | Down | 0.00260826 | 0.00734334 | 0.22 |
| hsa-miR-518f* | 1.12 | Down | 0.00603095 | 0.01459297 | 0.33 |
| hsa-miR-370 | 1.12 | Down | 0.01325454 | 0.02802271 | 0.28 |
| hsa-miR-4288 | 1.12 | Down | 0.00119034 | 0.00392977 | 0.18 |
| hsa-miR-376a* | 1.12 | Down | 0.01369259 | 0.02874489 | 0.26 |
| hsa-miR-539 | 1.11 | Down | 0.02212693 | 0.04314393 | 0.28 |
| hsa-miR-548h | 1.10 | Down | 0.00387767 | 0.01002701 | 0.27 |
| hsa-miR-1537 | 1.10 | Down | 0.02025606 | 0.04021178 | 0.30 |
| hsa-miR-512-3p | 1.09 | Down | 0.02254498 | 0.04374312 | 0.27 |
| hsa-miR-3681 | 1.08 | Down | 0.02574213 | 0.04900358 | 0.37 |
| hsa-miR-619 | 1.08 | Down | 0.00643846 | 0.01544828 | 0.31 |
| hsa-miR-186* | 1.07 | Down | 0.02316014 | 0.0447961 | 0.26 |
| hsa-miR-4320 | 1.07 | Down | 0.00065536 | 0.00250771 | 0.22 |
| hsa-miR-609 | 1.02 | Down | 0.01781487 | 0.03632304 | 0.38 |
|  |  |  |  |  |  |
|  |  |  |  |  |  |
| **B) TOF-noHF patients (n=18) compared to matched healthy controls (n=15)** | | | | | |
| **miRNA** | **Fold Change** | **Regulation** | **Ttest** | **Ttest Adj** | **AUC** |
| hsa-miR-1231 | 8.72 | Up | 0.00017052 | 0.00092978 | 0.86 |
| hsa-miR-144* | 7.39 | Up | 0.00015572 | 0.00086073 | 0.85 |
| hsa-miR-505* | 4.54 | Up | 0.00055102 | 0.00216985 | 0.83 |
| hsa-miR-214 | 3.33 | Up | 0.00972244 | 0.02181666 | 0.76 |
| hsa-miR-625 | 3.23 | Up | 0.01481807 | 0.03116191 | 0.76 |
| hsa-miR-218-1* | 3.18 | Up | 4.78E-05 | 0.00037403 | 0.95 |
| hsa-miR-15b | 3.00 | Up | 0.00058219 | 0.0022702 | 0.82 |
| hsa-miR-1233 | 2.91 | Up | 4.26E-05 | 0.00035304 | 0.86 |
| hsa-let-7a | 2.66 | Up | 1.12E-05 | 0.0001603 | 0.92 |
| hsa-miR-34c-3p | 2.61 | Up | 0.0001118 | 0.0006736 | 0.85 |
| hsa-let-7f | 2.58 | Up | 0.00070245 | 0.0026206 | 0.84 |
| hsa-miR-218-2* | 2.58 | Up | 8.26E-08 | 1.86E-05 | 0.96 |
| hsa-miR-138-1* | 2.48 | Up | 1.12E-05 | 0.0001603 | 0.90 |
| hsa-miR-20b | 2.48 | Up | 0.00026207 | 0.00125813 | 0.86 |
| hsa-miR-20a | 2.48 | Up | 0.00039072 | 0.00164048 | 0.84 |
| hsa-miR-1181 | 2.34 | Up | 0.000394 | 0.00164281 | 0.85 |
| hsa-miR-4286 | 2.32 | Up | 0.00146576 | 0.00457574 | 0.78 |
| hsa-miR-1274b | 2.31 | Up | 3.43E-06 | 7.64E-05 | 0.91 |
| hsa-let-7d | 2.24 | Up | 1.91E-05 | 0.00022533 | 0.91 |
| hsa-miR-3690 | 2.23 | Up | 7.48E-08 | 1.86E-05 | 0.99 |
| hsa-miR-1292 | 2.21 | Up | 0.00125062 | 0.00410626 | 0.75 |
| hsa-miR-4258 | 2.20 | Up | 1.36E-05 | 0.00018864 | 0.91 |
| hsa-miR-1274a | 2.18 | Up | 0.00038077 | 0.00162706 | 0.85 |
| hsa-miR-1288 | 2.16 | Up | 8.93E-05 | 0.00057476 | 0.87 |
| hsa-miR-183 | 2.11 | Up | 0.00037103 | 0.00159674 | 0.87 |
| hsa-miR-4319 | 2.10 | Up | 3.92E-05 | 0.00033303 | 0.86 |
| hsa-let-7g | 2.08 | Up | 3.15E-05 | 0.00028956 | 0.90 |
| hsa-miR-124* | 2.06 | Up | 2.28E-05 | 0.00023501 | 0.94 |
| hsa-miR-887 | 2.04 | Up | 3.72E-06 | 7.94E-05 | 0.91 |
| hsa-miR-921 | 2.02 | Up | 2.77E-06 | 6.68E-05 | 0.90 |
| hsa-miR-3907 | 2.02 | Up | 0.00042959 | 0.00176075 | 0.84 |
| hsa-miR-196b* | 2.01 | Up | 6.84E-08 | 1.86E-05 | 0.96 |
| hsa-let-7b | 2.01 | Up | 0.00696441 | 0.01642293 | 0.76 |
| hsa-miR-3935 | 1.99 | Up | 0.01113017 | 0.02442961 | 0.82 |
| hsa-miR-1914 | 1.97 | Up | 5.30E-06 | 9.33E-05 | 0.89 |
| hsa-miR-891a | 1.95 | Up | 6.48E-08 | 1.86E-05 | 0.96 |
| hsa-miR-588 | 1.94 | Up | 2.28E-05 | 0.00023501 | 0.86 |
| hsa-miR-4279 | 1.91 | Up | 4.90E-07 | 2.46E-05 | 0.93 |
| hsa-miR-1271 | 1.91 | Up | 0.00603638 | 0.01457682 | 0.76 |
| hsa-miR-1469 | 1.90 | Up | 0.0018458 | 0.0055466 | 0.76 |
| hsa-miR-554 | 1.90 | Up | 0.00018756 | 0.00097001 | 0.86 |
| hsa-miR-3126-5p | 1.89 | Up | 1.77E-06 | 4.84E-05 | 0.96 |
| hsa-miR-411* | 1.88 | Up | 7.70E-06 | 0.00012534 | 0.90 |
| hsa-miR-33a* | 1.87 | Up | 4.36E-07 | 2.46E-05 | 0.95 |
| hsa-miR-2114* | 1.86 | Up | 4.63E-06 | 8.72E-05 | 0.93 |
| hsa-miR-2277-3p | 1.86 | Up | 6.18E-06 | 0.0001049 | 0.91 |
| hsa-miR-466 | 1.85 | Up | 0.00112815 | 0.00376571 | 0.81 |
| hsa-miR-3667-5p | 1.85 | Up | 0.00985917 | 0.02200055 | 0.76 |
| hsa-miR-943 | 1.84 | Up | 4.73E-07 | 2.46E-05 | 0.94 |
| hsa-miR-3125 | 1.84 | Up | 0.00093473 | 0.00328381 | 0.80 |
| hsa-miR-3124 | 1.84 | Up | 0.00422972 | 0.01075277 | 0.81 |
| hsa-miR-4321 | 1.83 | Up | 0.00131058 | 0.00421132 | 0.81 |
| hsa-miR-1273c | 1.83 | Up | 4.37E-05 | 0.00035681 | 0.87 |
| hsa-miR-675* | 1.82 | Up | 1.68E-05 | 0.0002041 | 0.89 |
| hsa-miR-449b* | 1.81 | Up | 7.36E-05 | 0.00049253 | 0.86 |
| hsa-miR-3677 | 1.81 | Up | 4.34E-06 | 8.31E-05 | 0.91 |
| hsa-miR-3181 | 1.80 | Up | 0.00067124 | 0.00255964 | 0.79 |
| hsa-miR-1305 | 1.79 | Up | 0.00073288 | 0.00271728 | 0.84 |
| hsa-miR-3178 | 1.79 | Up | 0.00068412 | 0.00258422 | 0.84 |
| hsa-miR-28-5p | 1.79 | Up | 0.01847081 | 0.03728195 | 0.74 |
| hsa-miR-1251 | 1.79 | Up | 1.27E-06 | 4.02E-05 | 0.92 |
| hsa-miR-2355-5p | 1.79 | Up | 2.40E-05 | 0.0002409 | 0.89 |
| hsa-miR-744* | 1.78 | Up | 1.11E-05 | 0.0001603 | 0.91 |
| hsa-miR-663 | 1.77 | Up | 0.0027977 | 0.00784006 | 0.80 |
| hsa-miR-331-5p | 1.76 | Up | 3.28E-06 | 7.45E-05 | 0.91 |
| hsa-miR-668 | 1.76 | Up | 5.97E-07 | 2.58E-05 | 0.91 |
| hsa-miR-24-2* | 1.76 | Up | 0.00259385 | 0.00738909 | 0.79 |
| hsa-miR-27a | 1.75 | Up | 0.00645715 | 0.01544876 | 0.76 |
| hsa-miR-676* | 1.75 | Up | 1.00E-06 | 3.49E-05 | 0.94 |
| hsa-miR-3126-3p | 1.75 | Up | 2.03E-07 | 2.02E-05 | 0.99 |
| hsa-miR-3198 | 1.75 | Up | 0.00168919 | 0.00515726 | 0.80 |
| hsa-miR-657 | 1.75 | Up | 2.21E-05 | 0.00023501 | 0.90 |
| hsa-miR-4262 | 1.74 | Up | 2.06E-05 | 0.00023253 | 0.89 |
| hsa-miR-4305 | 1.73 | Up | 4.57E-07 | 2.46E-05 | 0.95 |
| hsa-miR-431 | 1.72 | Up | 0.00018158 | 0.00094783 | 0.86 |
| hsa-miR-4293 | 1.71 | Up | 0.00175595 | 0.00531639 | 0.79 |
| hsa-miR-148a* | 1.71 | Up | 1.72E-06 | 4.84E-05 | 0.92 |
| hsa-miR-631 | 1.71 | Up | 0.00263676 | 0.00747716 | 0.82 |
| hsa-miR-3160 | 1.70 | Up | 7.57E-05 | 0.00050359 | 0.85 |
| hsa-let-7c* | 1.70 | Up | 2.96E-07 | 2.14E-05 | 0.94 |
| hsa-let-7i | 1.69 | Up | 0.00719619 | 0.01690333 | 0.83 |
| hsa-miR-3918 | 1.68 | Up | 0.00035554 | 0.00156358 | 0.81 |
| hsa-miR-596 | 1.68 | Up | 4.62E-05 | 0.00036832 | 0.85 |
| hsa-miR-4329 | 1.68 | Up | 0.0001925 | 0.00098291 | 0.83 |
| hsa-miR-363 | 1.68 | Up | 2.73E-05 | 0.00026313 | 0.87 |
| hsa-miR-4269 | 1.67 | Up | 5.03E-05 | 0.00038228 | 0.89 |
| hsa-miR-934 | 1.66 | Up | 0.00934355 | 0.02112379 | 0.77 |
| hsa-miR-193a-3p | 1.66 | Up | 2.44E-06 | 6.25E-05 | 0.91 |
| hsa-miR-4301 | 1.66 | Up | 1.36E-07 | 1.86E-05 | 0.95 |
| hsa-miR-1913 | 1.66 | Up | 0.00010266 | 0.00063766 | 0.85 |
| hsa-miR-1286 | 1.65 | Up | 4.28E-05 | 0.00035304 | 0.89 |
| hsa-miR-143 | 1.63 | Up | 2.94E-05 | 0.000275 | 0.89 |
| hsa-miR-1260b | 1.62 | Up | 0.00053488 | 0.00212017 | 0.85 |
| hsa-miR-409-5p | 1.61 | Up | 3.44E-05 | 0.0003028 | 0.89 |
| hsa-miR-4304 | 1.61 | Up | 4.84E-05 | 0.00037517 | 0.85 |
| hsa-miR-4275 | 1.61 | Up | 1.58E-07 | 1.86E-05 | 0.96 |
| hsa-miR-3184 | 1.60 | Up | 1.43E-06 | 4.41E-05 | 0.94 |
| hsa-miR-505 | 1.60 | Up | 0.01839602 | 0.03719329 | 0.73 |
| hsa-miR-380* | 1.60 | Up | 0.005626 | 0.01372333 | 0.79 |
| hsa-miR-3941 | 1.59 | Up | 0.00302799 | 0.00831479 | 0.79 |
| hsa-miR-550b | 1.59 | Up | 0.00017437 | 0.00093148 | 0.84 |
| hsa-miR-299-5p | 1.58 | Up | 0.0026532 | 0.00748736 | 0.80 |
| hsa-miR-3622a-3p | 1.58 | Up | 1.76E-06 | 4.84E-05 | 0.92 |
| hsa-miR-296-5p | 1.58 | Up | 0.00208371 | 0.00610919 | 0.81 |
| hsa-miR-621 | 1.58 | Up | 1.03E-05 | 0.00015497 | 0.90 |
| hsa-miR-3622b-3p | 1.58 | Up | 9.01E-05 | 0.00057476 | 0.86 |
| hsa-miR-3939 | 1.57 | Up | 3.66E-06 | 7.94E-05 | 0.92 |
| hsa-miR-1247 | 1.57 | Up | 0.00022043 | 0.00110676 | 0.83 |
| hsa-miR-34b | 1.57 | Up | 2.98E-05 | 0.00027633 | 0.88 |
| hsa-miR-633 | 1.57 | Up | 2.09E-06 | 5.59E-05 | 0.91 |
| hsa-miR-107 | 1.57 | Up | 0.00140678 | 0.00442375 | 0.83 |
| hsa-miR-1910 | 1.56 | Up | 0.01267238 | 0.02721965 | 0.74 |
| hsa-miR-4285 | 1.56 | Up | 0.00565144 | 0.01375754 | 0.85 |
| hsa-miR-455-3p | 1.56 | Up | 2.74E-06 | 6.68E-05 | 0.92 |
| hsa-miR-4252 | 1.56 | Up | 0.00054058 | 0.00213572 | 0.83 |
| hsa-miR-151-5p | 1.56 | Up | 0.01461371 | 0.0308398 | 0.70 |
| hsa-miR-192* | 1.55 | Up | 4.69E-05 | 0.00036919 | 0.87 |
| hsa-miR-1249 | 1.55 | Up | 0.00182674 | 0.00551684 | 0.78 |
| hsa-miR-1291 | 1.54 | Up | 0.00663938 | 0.01581118 | 0.77 |
| hsa-miR-3139 | 1.54 | Up | 1.06E-08 | 1.28E-05 | 0.99 |
| hsa-miR-647 | 1.54 | Up | 1.64E-05 | 0.00020364 | 0.89 |
| hsa-miR-541 | 1.53 | Up | 0.00015167 | 0.00084611 | 0.84 |
| hsa-miR-2117 | 1.53 | Up | 1.59E-05 | 0.00020364 | 0.87 |
| hsa-miR-663b | 1.53 | Up | 0.00020562 | 0.00104545 | 0.82 |
| hsa-let-7g* | 1.53 | Up | 1.07E-06 | 3.54E-05 | 0.92 |
| hsa-miR-2115 | 1.53 | Up | 2.25E-05 | 0.00023501 | 0.89 |
| hsa-miR-582-3p | 1.53 | Up | 0.00011044 | 0.00066876 | 0.85 |
| hsa-miR-122* | 1.52 | Up | 0.00011838 | 0.00070619 | 0.88 |
| hsa-miR-600 | 1.52 | Up | 5.39E-05 | 0.00040308 | 0.89 |
| hsa-miR-1193 | 1.52 | Up | 0.00753948 | 0.01760672 | 0.81 |
| hsa-miR-3944 | 1.52 | Up | 5.01E-06 | 9.15E-05 | 0.93 |
| hsa-miR-3177 | 1.52 | Up | 0.00085583 | 0.00309692 | 0.79 |
| hsa-miR-491-5p | 1.51 | Up | 0.01220926 | 0.0264273 | 0.76 |
| hsa-miR-7-2* | 1.51 | Up | 5.92E-06 | 0.00010184 | 0.91 |
| hsa-miR-1260 | 1.51 | Up | 0.00088029 | 0.00316643 | 0.81 |
| hsa-miR-20b* | 1.51 | Up | 2.39E-06 | 6.25E-05 | 0.92 |
| hsa-miR-153 | 1.51 | Up | 0.00516362 | 0.01280281 | 0.75 |
| hsa-miR-346 | 1.51 | Up | 6.67E-05 | 0.00047305 | 0.86 |
| hsa-miR-1909 | 1.50 | Up | 7.61E-05 | 0.00050359 | 0.86 |
| hsa-miR-1296 | 1.50 | Up | 0.00020714 | 0.00104875 | 0.86 |
| hsa-miR-155* | 1.50 | Up | 0.00108317 | 0.00366635 | 0.86 |
| hsa-miR-205 | 1.49 | Up | 0.00022549 | 0.00112744 | 0.84 |
| hsa-miR-362-3p | 1.49 | Up | 0.00705834 | 0.01661192 | 0.77 |
| hsa-miR-3685 | 1.49 | Up | 6.86E-05 | 0.00047985 | 0.88 |
| hsa-miR-597 | 1.49 | Up | 0.00086792 | 0.00313126 | 0.84 |
| hsa-miR-4277 | 1.49 | Up | 0.00042602 | 0.00175208 | 0.80 |
| hsa-miR-2277-5p | 1.49 | Up | 3.40E-05 | 0.00030114 | 0.91 |
| hsa-miR-542-5p | 1.48 | Up | 0.00055363 | 0.00217305 | 0.83 |
| hsa-miR-34a* | 1.48 | Up | 1.25E-05 | 0.00017564 | 0.87 |
| hsa-miR-377 | 1.47 | Up | 0.00010463 | 0.00063998 | 0.88 |
| hsa-miR-4330 | 1.47 | Up | 0.00139912 | 0.00441345 | 0.77 |
| hsa-miR-487b | 1.47 | Up | 0.00043689 | 0.00177255 | 0.82 |
| hsa-miR-3678-5p | 1.47 | Up | 8.81E-06 | 0.00013781 | 0.91 |
| hsa-miR-328 | 1.47 | Up | 0.0210272 | 0.04167397 | 0.72 |
| hsa-miR-885-5p | 1.47 | Up | 9.23E-05 | 0.00058536 | 0.89 |
| hsa-miR-615-5p | 1.47 | Up | 6.97E-05 | 0.00047985 | 0.89 |
| hsa-miR-640 | 1.47 | Up | 4.41E-05 | 0.00035681 | 0.87 |
| hsa-miR-224* | 1.47 | Up | 5.43E-07 | 2.52E-05 | 0.91 |
| hsa-miR-1224-3p | 1.47 | Up | 0.00011264 | 0.00067531 | 0.85 |
| hsa-miR-342-5p | 1.46 | Up | 0.00844977 | 0.0194684 | 0.74 |
| hsa-miR-891b | 1.46 | Up | 0.00121585 | 0.00400715 | 0.83 |
| hsa-miR-3158 | 1.46 | Up | 4.29E-06 | 8.31E-05 | 0.93 |
| hsa-miR-708 | 1.46 | Up | 0.0001038 | 0.00063813 | 0.92 |
| hsa-miR-487a | 1.45 | Up | 3.89E-06 | 7.94E-05 | 0.93 |
| hsa-miR-506 | 1.45 | Up | 4.14E-06 | 8.31E-05 | 0.90 |
| hsa-miR-3615 | 1.45 | Up | 0.00509234 | 0.0127045 | 0.79 |
| hsa-miR-4287 | 1.44 | Up | 7.28E-05 | 0.00049253 | 0.83 |
| hsa-miR-196a* | 1.44 | Up | 0.0023242 | 0.00670014 | 0.80 |
| hsa-miR-3658 | 1.43 | Up | 0.01600752 | 0.03302922 | 0.81 |
| hsa-miR-4326 | 1.43 | Up | 0.00129571 | 0.00418589 | 0.79 |
| hsa-miR-519e* | 1.43 | Up | 0.02593758 | 0.04961076 | 0.73 |
| hsa-miR-3670 | 1.43 | Up | 0.00013495 | 0.00078182 | 0.89 |
| hsa-miR-3684 | 1.43 | Up | 5.61E-05 | 0.00041493 | 0.89 |
| hsa-miR-489 | 1.43 | Up | 0.01105389 | 0.02435089 | 0.86 |
| hsa-miR-502-3p | 1.42 | Up | 0.00515735 | 0.01280281 | 0.76 |
| hsa-let-7e* | 1.42 | Up | 5.99E-07 | 2.58E-05 | 0.93 |
| hsa-miR-4254 | 1.42 | Up | 0.00038504 | 0.00162872 | 0.85 |
| hsa-miR-607 | 1.42 | Up | 0.00131448 | 0.00421264 | 0.82 |
| hsa-miR-133a | 1.42 | Up | 0.00370571 | 0.00979452 | 0.79 |
| hsa-miR-31* | 1.41 | Up | 1.52E-05 | 0.00020363 | 0.91 |
| hsa-miR-3176 | 1.41 | Up | 0.00017272 | 0.00093148 | 0.85 |
| hsa-miR-770-5p | 1.41 | Up | 0.00116699 | 0.00388461 | 0.82 |
| hsa-miR-935 | 1.40 | Up | 0.0001817 | 0.00094783 | 0.83 |
| hsa-miR-718 | 1.40 | Up | 0.00169585 | 0.00516036 | 0.78 |
| hsa-let-7e | 1.39 | Up | 0.00413966 | 0.01059084 | 0.78 |
| hsa-miR-1236 | 1.39 | Up | 4.45E-05 | 0.00035723 | 0.87 |
| hsa-miR-379* | 1.39 | Up | 1.55E-06 | 4.58E-05 | 0.94 |
| hsa-miR-3127 | 1.38 | Up | 0.02566466 | 0.04932363 | 0.70 |
| hsa-miR-650 | 1.37 | Up | 0.00110945 | 0.00372393 | 0.78 |
| hsa-miR-764 | 1.37 | Up | 0.0009603 | 0.00334439 | 0.83 |
| hsa-miR-758 | 1.37 | Up | 0.00035928 | 0.00156888 | 0.86 |
| hsa-miR-1915* | 1.37 | Up | 0.00912287 | 0.02070256 | 0.77 |
| hsa-miR-636 | 1.37 | Up | 0.00743868 | 0.01743894 | 0.74 |
| hsa-miR-103 | 1.37 | Up | 0.00939901 | 0.02120937 | 0.73 |
| hsa-miR-3128 | 1.36 | Up | 0.0003094 | 0.00140409 | 0.83 |
| hsa-miR-509-3-5p | 1.36 | Up | 0.00059214 | 0.00228946 | 0.82 |
| hsa-miR-1976 | 1.36 | Up | 0.00271821 | 0.00763507 | 0.78 |
| hsa-miR-3122 | 1.36 | Up | 0.00018116 | 0.00094783 | 0.84 |
| hsa-miR-892b | 1.35 | Up | 0.00812126 | 0.01874735 | 0.77 |
| hsa-miR-216b | 1.35 | Up | 0.00398774 | 0.01028957 | 0.81 |
| hsa-miR-3910 | 1.35 | Up | 0.00022702 | 0.00113042 | 0.87 |
| hsa-miR-3161 | 1.35 | Up | 0.00026739 | 0.00127858 | 0.84 |
| hsa-miR-606 | 1.35 | Up | 0.01461245 | 0.0308398 | 0.74 |
| hsa-miR-105 | 1.34 | Up | 0.00059279 | 0.00228946 | 0.83 |
| hsa-miR-345 | 1.34 | Up | 0.01476765 | 0.03111018 | 0.76 |
| hsa-miR-654-5p | 1.34 | Up | 0.00125666 | 0.00411489 | 0.83 |
| hsa-miR-1208 | 1.34 | Up | 0.00098362 | 0.00341574 | 0.82 |
| hsa-miR-24-1* | 1.34 | Up | 0.00746978 | 0.01747784 | 0.76 |
| hsa-miR-499-3p | 1.34 | Up | 0.0003736 | 0.00160208 | 0.86 |
| hsa-miR-1276 | 1.34 | Up | 0.00376104 | 0.00989532 | 0.81 |
| hsa-miR-4328 | 1.34 | Up | 0.00397292 | 0.01027333 | 0.74 |
| hsa-miR-30e* | 1.34 | Up | 0.02214079 | 0.04361864 | 0.72 |
| hsa-miR-3130-5p | 1.33 | Up | 0.00366208 | 0.00974128 | 0.76 |
| hsa-miR-412 | 1.32 | Up | 0.00156711 | 0.00484196 | 0.83 |
| hsa-miR-635 | 1.32 | Up | 0.00100576 | 0.0034726 | 0.84 |
| hsa-miR-593* | 1.32 | Up | 1.66E-05 | 0.0002041 | 0.91 |
| hsa-miR-200a* | 1.31 | Up | 0.00048408 | 0.00194441 | 0.82 |
| hsa-miR-3620 | 1.31 | Up | 0.01045384 | 0.02311354 | 0.73 |
| hsa-miR-486-3p | 1.30 | Up | 0.00299791 | 0.00828552 | 0.81 |
| hsa-miR-4260 | 1.30 | Up | 0.01834408 | 0.03715062 | 0.77 |
| hsa-miR-1263 | 1.30 | Up | 0.00137014 | 0.00433337 | 0.86 |
| hsa-miR-548v | 1.30 | Up | 8.70E-06 | 0.00013781 | 0.93 |
| hsa-miR-4297 | 1.30 | Up | 0.00029666 | 0.00136965 | 0.89 |
| hsa-let-7i* | 1.30 | Up | 8.93E-07 | 3.36E-05 | 0.93 |
| hsa-miR-3675-3p | 1.30 | Up | 0.00409445 | 0.01049748 | 0.79 |
| hsa-miR-513a-3p | 1.30 | Up | 0.0023594 | 0.00676923 | 0.83 |
| hsa-miR-589* | 1.30 | Up | 0.02560312 | 0.04932363 | 0.73 |
| hsa-miR-1280 | 1.29 | Up | 0.00407311 | 0.01046503 | 0.78 |
| hsa-miR-1203 | 1.29 | Up | 0.00111438 | 0.00373008 | 0.84 |
| hsa-miR-3194 | 1.29 | Up | 0.00188701 | 0.00561443 | 0.76 |
| hsa-miR-558 | 1.29 | Up | 0.00187435 | 0.00560443 | 0.77 |
| hsa-miR-1178 | 1.29 | Up | 6.54E-07 | 2.72E-05 | 0.94 |
| hsa-miR-363* | 1.29 | Up | 0.02363666 | 0.04623729 | 0.72 |
| hsa-miR-1229 | 1.29 | Up | 0.0030292 | 0.00831479 | 0.76 |
| hsa-miR-604 | 1.29 | Up | 0.00171021 | 0.00519093 | 0.77 |
| hsa-miR-3667-3p | 1.28 | Up | 0.0002297 | 0.00113905 | 0.86 |
| hsa-miR-1226 | 1.28 | Up | 0.0016645 | 0.00510363 | 0.83 |
| hsa-miR-611 | 1.28 | Up | 0.0032187 | 0.00875516 | 0.79 |
| hsa-miR-3666 | 1.28 | Up | 0.00019189 | 0.00098291 | 0.86 |
| hsa-miR-3142 | 1.28 | Up | 0.0001456 | 0.00081602 | 0.84 |
| hsa-miR-937 | 1.27 | Up | 0.001417 | 0.00443504 | 0.84 |
| hsa-miR-1470 | 1.27 | Up | 0.02081962 | 0.04139874 | 0.72 |
| hsa-miR-4268 | 1.27 | Up | 0.00525565 | 0.01297487 | 0.82 |
| hsa-miR-504 | 1.27 | Up | 0.00011942 | 0.00070886 | 0.86 |
| hsa-miR-590-5p | 1.27 | Up | 0.01454258 | 0.03079757 | 0.73 |
| hsa-miR-888* | 1.27 | Up | 0.00228241 | 0.00662724 | 0.80 |
| hsa-miR-496 | 1.26 | Up | 0.01265157 | 0.02721965 | 0.83 |
| hsa-miR-182* | 1.26 | Up | 0.00027952 | 0.00130551 | 0.88 |
| hsa-miR-1289 | 1.26 | Up | 8.34E-07 | 3.24E-05 | 0.92 |
| hsa-miR-1197 | 1.26 | Up | 2.62E-05 | 0.00025498 | 0.90 |
| hsa-miR-1237 | 1.26 | Up | 0.00047993 | 0.00193418 | 0.85 |
| hsa-miR-488* | 1.25 | Up | 0.00109495 | 0.00369585 | 0.79 |
| hsa-miR-509-5p | 1.25 | Up | 0.02454021 | 0.04769509 | 0.73 |
| hsa-miR-103-as | 1.25 | Up | 0.00058403 | 0.0022702 | 0.83 |
| hsa-miR-3165 | 1.25 | Up | 0.00109882 | 0.00369854 | 0.87 |
| hsa-miR-4294 | 1.25 | Up | 0.00947182 | 0.02133373 | 0.79 |
| hsa-miR-515-5p | 1.25 | Up | 0.01201347 | 0.02617763 | 0.76 |
| hsa-miR-1200 | 1.25 | Up | 0.00119261 | 0.00394807 | 0.82 |
| hsa-miR-605 | 1.25 | Up | 0.00068287 | 0.00258422 | 0.85 |
| hsa-miR-1253 | 1.24 | Up | 0.00346695 | 0.00934022 | 0.81 |
| hsa-miR-3157 | 1.24 | Up | 0.00094284 | 0.00329311 | 0.85 |
| hsa-miR-3133 | 1.24 | Up | 0.00693387 | 0.01638296 | 0.77 |
| hsa-miR-1227 | 1.24 | Up | 0.00435444 | 0.01104652 | 0.77 |
| hsa-miR-624* | 1.24 | Up | 0.00355872 | 0.00952947 | 0.79 |
| hsa-miR-212 | 1.24 | Up | 0.0121842 | 0.0264273 | 0.73 |
| hsa-miR-106a* | 1.23 | Up | 0.00659725 | 0.01574195 | 0.79 |
| hsa-miR-32 | 1.23 | Up | 0.01501376 | 0.03145692 | 0.80 |
| hsa-miR-555 | 1.23 | Up | 3.73E-05 | 0.00032078 | 0.88 |
| hsa-miR-502-5p | 1.23 | Up | 0.02504941 | 0.04845465 | 0.71 |
| hsa-miR-582-5p | 1.22 | Up | 0.00301489 | 0.00831338 | 0.85 |
| hsa-miR-1273 | 1.22 | Up | 0.00016403 | 0.00090254 | 0.85 |
| hsa-miR-518b | 1.22 | Up | 0.00245905 | 0.00702169 | 0.80 |
| hsa-miR-18a | 1.22 | Up | 0.01305964 | 0.02790225 | 0.71 |
| hsa-miR-551a | 1.21 | Up | 4.27E-06 | 8.31E-05 | 0.96 |
| hsa-miR-616* | 1.21 | Up | 8.17E-06 | 0.0001313 | 0.91 |
| hsa-miR-548s | 1.21 | Up | 0.01551161 | 0.03222671 | 0.76 |
| hsa-miR-2113 | 1.21 | Up | 0.00372776 | 0.00982922 | 0.81 |
| hsa-miR-877* | 1.20 | Up | 0.00292904 | 0.00815125 | 0.79 |
| hsa-miR-3120 | 1.20 | Up | 0.01624862 | 0.03346939 | 0.73 |
| hsa-miR-1298 | 1.20 | Up | 0.00688811 | 0.01633893 | 0.79 |
| hsa-miR-200c* | 1.20 | Up | 0.01506277 | 0.03145692 | 0.74 |
| hsa-miR-551b | 1.20 | Up | 7.36E-07 | 2.95E-05 | 0.95 |
| hsa-miR-216a | 1.20 | Up | 0.00582099 | 0.01408492 | 0.77 |
| hsa-miR-525-5p | 1.19 | Up | 0.00691753 | 0.01637648 | 0.76 |
| hsa-miR-222* | 1.19 | Up | 0.01912407 | 0.0384075 | 0.79 |
| hsa-miR-625* | 1.19 | Up | 0.02414308 | 0.0471514 | 0.71 |
| hsa-miR-634 | 1.19 | Up | 0.00169055 | 0.00515726 | 0.83 |
| hsa-miR-548z | 1.19 | Up | 0.00559463 | 0.01372333 | 0.87 |
| hsa-miR-3074 | 1.19 | Up | 5.04E-05 | 0.00038228 | 0.93 |
| hsa-miR-661 | 1.19 | Up | 0.00445679 | 0.01123522 | 0.82 |
| hsa-miR-3692 | 1.18 | Up | 0.01206968 | 0.02625264 | 0.74 |
| hsa-miR-767-3p | 1.18 | Up | 0.00067415 | 0.00256262 | 0.82 |
| hsa-miR-2909 | 1.18 | Up | 0.00561732 | 0.01372333 | 0.75 |
| hsa-miR-514b-3p | 1.18 | Up | 0.00465426 | 0.01168412 | 0.79 |
| hsa-miR-19b-1* | 1.18 | Up | 0.00984049 | 0.02199962 | 0.80 |
| hsa-miR-1269 | 1.17 | Up | 0.00578602 | 0.01405677 | 0.75 |
| hsa-miR-1204 | 1.17 | Up | 0.00043486 | 0.00177031 | 0.85 |
| hsa-miR-589 | 1.17 | Up | 0.00068822 | 0.00258458 | 0.89 |
| hsa-miR-3679-3p | 1.16 | Up | 0.01372119 | 0.02926377 | 0.78 |
| hsa-miR-4276 | 1.16 | Up | 0.0101878 | 0.02265 | 0.79 |
| hsa-miR-523 | 1.16 | Up | 0.01178243 | 0.02572071 | 0.71 |
| hsa-miR-548p | 1.16 | Up | 0.00184338 | 0.0055466 | 0.82 |
| hsa-miR-3921 | 1.15 | Up | 0.00242907 | 0.00695257 | 0.79 |
| hsa-miR-675 | 1.15 | Up | 0.0093078 | 0.02108252 | 0.76 |
| hsa-miR-18b* | 1.14 | Up | 0.01970983 | 0.03938696 | 0.75 |
| hsa-miR-124 | 1.14 | Up | 0.00849042 | 0.01952473 | 0.73 |
| hsa-miR-1538 | 1.14 | Up | 0.00982452 | 0.02199962 | 0.82 |
| hsa-miR-15a* | 1.14 | Up | 0.00017407 | 0.00093148 | 0.89 |
| hsa-miR-1225-3p | 1.13 | Up | 0.00073743 | 0.00272576 | 0.80 |
| hsa-miR-3922 | 1.13 | Up | 0.01655304 | 0.03392248 | 0.77 |
| hsa-miR-570 | 1.12 | Up | 0.0128712 | 0.02754848 | 0.75 |
| hsa-miR-29b-2* | 1.10 | Up | 0.01673205 | 0.03417309 | 0.70 |
| hsa-miR-127-3p | 1.10 | Up | 0.01388172 | 0.02955383 | 0.74 |
| hsa-miR-491-3p | 1.09 | Up | 0.00894628 | 0.02037857 | 0.80 |
| hsa-miR-432* | 1.09 | Up | 0.01227072 | 0.02649859 | 0.79 |
| hsa-miR-185* | 1.08 | Up | 0.00379606 | 0.0099657 | 0.82 |
| hsa-miR-219-5p | 1.07 | Up | 0.0242917 | 0.04728837 | 0.72 |
| hsa-miR-615-3p | 1.06 | Up | 0.01253096 | 0.02701218 | 0.73 |
| hsa-miR-1324 | 1.04 | Up | 0.01992751 | 0.03969034 | 0.69 |
| hsa-miR-4306 | 1.00 | Down | 0.00285024 | 0.00796878 | 0.24 |
| hsa-miR-518d-3p | 1.07 | Down | 0.01661386 | 0.03398932 | 0.25 |
| hsa-miR-186* | 1.08 | Down | 0.02590191 | 0.04961076 | 0.24 |
| hsa-miR-619 | 1.09 | Down | 0.00624029 | 0.01503911 | 0.29 |
| hsa-miR-421 | 1.10 | Down | 0.01771634 | 0.03593971 | 0.30 |
| hsa-miR-4288 | 1.12 | Down | 0.00235539 | 0.00676923 | 0.19 |
| hsa-miR-873 | 1.12 | Down | 0.0111002 | 0.02440828 | 0.25 |
| hsa-miR-539 | 1.13 | Down | 0.02300251 | 0.04514336 | 0.28 |
| hsa-miR-524-3p | 1.16 | Down | 0.02305143 | 0.0451658 | 0.23 |
| hsa-miR-760 | 1.16 | Down | 0.01640521 | 0.03373426 | 0.32 |
| hsa-miR-3148 | 1.16 | Down | 0.00757504 | 0.01765555 | 0.33 |
| hsa-miR-550a | 1.16 | Down | 0.00346272 | 0.00934022 | 0.20 |
| hsa-miR-3687 | 1.16 | Down | 0.01509778 | 0.03147547 | 0.29 |
| hsa-miR-302a* | 1.17 | Down | 0.00526532 | 0.01297487 | 0.24 |
| hsa-miR-3928 | 1.17 | Down | 0.00630654 | 0.01513821 | 0.26 |
| hsa-miR-3689a-5p | 1.17 | Down | 0.0103229 | 0.02288404 | 0.37 |
| hsa-miR-1287 | 1.17 | Down | 0.00041198 | 0.00170013 | 0.16 |
| hsa-miR-30c-2* | 1.17 | Down | 0.02562461 | 0.04932363 | 0.20 |
| hsa-miR-185 | 1.18 | Down | 0.00872123 | 0.01994133 | 0.25 |
| hsa-miR-377* | 1.18 | Down | 8.40E-05 | 0.00054771 | 0.10 |
| hsa-miR-3912 | 1.18 | Down | 0.00580361 | 0.01407113 | 0.20 |
| hsa-miR-518f* | 1.18 | Down | 0.0042021 | 0.01072782 | 0.30 |
| hsa-miR-376a* | 1.19 | Down | 0.00492154 | 0.01230386 | 0.20 |
| hsa-miR-1267 | 1.19 | Down | 0.01080837 | 0.02385364 | 0.26 |
| hsa-miR-425* | 1.19 | Down | 0.02209355 | 0.04361864 | 0.29 |
| hsa-miR-614 | 1.19 | Down | 0.003634 | 0.00968798 | 0.25 |
| hsa-miR-1282 | 1.20 | Down | 0.01857826 | 0.03743612 | 0.33 |
| hsa-miR-374b* | 1.20 | Down | 0.01883385 | 0.0378878 | 0.23 |
| hsa-miR-302b | 1.20 | Down | 0.0048426 | 0.01213167 | 0.23 |
| hsa-miR-3657 | 1.20 | Down | 0.00393316 | 0.01021435 | 0.23 |
| hsa-miR-136 | 1.20 | Down | 0.00442636 | 0.0111819 | 0.16 |
| hsa-miR-132* | 1.20 | Down | 0.00381577 | 0.00997397 | 0.25 |
| hsa-miR-548h | 1.20 | Down | 0.00297891 | 0.00825192 | 0.22 |
| hsa-miR-184 | 1.20 | Down | 0.00288963 | 0.00806019 | 0.13 |
| hsa-miR-4320 | 1.21 | Down | 0.00031855 | 0.00142697 | 0.17 |
| hsa-miR-203 | 1.21 | Down | 0.00013957 | 0.00079617 | 0.15 |
| hsa-miR-3945 | 1.21 | Down | 0.00329704 | 0.00892794 | 0.19 |
| hsa-miR-1179 | 1.21 | Down | 0.00264338 | 0.00747716 | 0.25 |
| hsa-miR-3681 | 1.21 | Down | 0.02588631 | 0.04961076 | 0.36 |
| hsa-miR-548w | 1.21 | Down | 0.01163661 | 0.02544848 | 0.33 |
| hsa-miR-568 | 1.22 | Down | 0.00188119 | 0.00561098 | 0.20 |
| hsa-miR-200a | 1.22 | Down | 0.01564497 | 0.03244783 | 0.27 |
| hsa-miR-19a* | 1.22 | Down | 0.01505877 | 0.03145692 | 0.27 |
| hsa-miR-599 | 1.22 | Down | 0.0116104 | 0.02543734 | 0.23 |
| hsa-miR-3925 | 1.22 | Down | 0.00100126 | 0.00346701 | 0.21 |
| hsa-miR-1304 | 1.22 | Down | 0.00224062 | 0.00652163 | 0.20 |
| hsa-miR-1254 | 1.22 | Down | 0.00029666 | 0.00136965 | 0.12 |
| hsa-miR-1255a | 1.23 | Down | 0.0159583 | 0.03302922 | 0.26 |
| hsa-miR-519b-3p | 1.23 | Down | 0.0023029 | 0.00667066 | 0.21 |
| hsa-miR-488 | 1.23 | Down | 0.00809966 | 0.01873337 | 0.23 |
| hsa-miR-448 | 1.23 | Down | 0.00076836 | 0.00283141 | 0.16 |
| hsa-miR-518c* | 1.23 | Down | 0.00764988 | 0.01779558 | 0.23 |
| hsa-miR-4289 | 1.23 | Down | 0.00017189 | 0.00093148 | 0.13 |
| hsa-miR-302b* | 1.23 | Down | 0.00367377 | 0.00975088 | 0.24 |
| hsa-miR-510 | 1.24 | Down | 0.01929035 | 0.038677 | 0.26 |
| hsa-miR-561 | 1.24 | Down | 0.00200988 | 0.00593603 | 0.24 |
| hsa-miR-3132 | 1.24 | Down | 0.02286509 | 0.04494687 | 0.34 |
| hsa-miR-1299 | 1.24 | Down | 0.00019151 | 0.00098291 | 0.16 |
| hsa-miR-648 | 1.24 | Down | 0.01033105 | 0.02288404 | 0.23 |
| hsa-miR-2116 | 1.25 | Down | 0.00625704 | 0.01504936 | 0.24 |
| hsa-miR-571 | 1.25 | Down | 0.00088518 | 0.00317451 | 0.11 |
| hsa-miR-3202 | 1.25 | Down | 0.01285717 | 0.02754848 | 0.27 |
| hsa-miR-765 | 1.25 | Down | 0.01409848 | 0.02996239 | 0.26 |
| hsa-miR-517c | 1.25 | Down | 0.00133133 | 0.00424404 | 0.21 |
| hsa-miR-581 | 1.25 | Down | 0.00117431 | 0.0038982 | 0.20 |
| hsa-miR-3908 | 1.25 | Down | 0.00102227 | 0.00351953 | 0.19 |
| hsa-miR-422a | 1.25 | Down | 0.00646156 | 0.01544876 | 0.23 |
| hsa-miR-3134 | 1.26 | Down | 4.92E-05 | 0.00037792 | 0.09 |
| hsa-miR-520b | 1.26 | Down | 0.00151892 | 0.00471727 | 0.19 |
| hsa-miR-3934 | 1.26 | Down | 0.02505166 | 0.04845465 | 0.30 |
| hsa-miR-1322 | 1.26 | Down | 0.01452304 | 0.03079757 | 0.23 |
| hsa-miR-3153 | 1.27 | Down | 6.10E-05 | 0.00044582 | 0.13 |
| hsa-miR-626 | 1.27 | Down | 0.00463499 | 0.01166004 | 0.26 |
| hsa-miR-367 | 1.27 | Down | 0.00015449 | 0.0008579 | 0.15 |
| hsa-miR-499-5p | 1.27 | Down | 8.98E-05 | 0.00057476 | 0.13 |
| hsa-miR-3675-5p | 1.27 | Down | 0.01550402 | 0.03222671 | 0.23 |
| hsa-miR-1323 | 1.27 | Down | 7.84E-05 | 0.00051623 | 0.14 |
| hsa-miR-1261 | 1.27 | Down | 0.00216629 | 0.00633586 | 0.21 |
| hsa-miR-3937 | 1.27 | Down | 0.00140973 | 0.00442375 | 0.19 |
| hsa-miR-3188 | 1.28 | Down | 0.01221577 | 0.0264273 | 0.25 |
| hsa-miR-3146 | 1.28 | Down | 0.00068851 | 0.00258458 | 0.14 |
| hsa-miR-135b | 1.28 | Down | 0.0221532 | 0.04361864 | 0.27 |
| hsa-miR-4255 | 1.28 | Down | 0.00560834 | 0.01372333 | 0.22 |
| hsa-miR-122 | 1.29 | Down | 9.69E-05 | 0.00061155 | 0.12 |
| hsa-miR-3167 | 1.29 | Down | 0.00059826 | 0.00230319 | 0.20 |
| hsa-miR-557 | 1.29 | Down | 0.00083976 | 0.00304791 | 0.17 |
| hsa-miR-3647-5p | 1.29 | Down | 0.01599606 | 0.03302922 | 0.24 |
| hsa-miR-450a | 1.29 | Down | 0.00026904 | 0.00128142 | 0.17 |
| hsa-miR-28-3p | 1.29 | Down | 0.00043118 | 0.00176127 | 0.17 |
| hsa-miR-4259 | 1.29 | Down | 0.00158227 | 0.00487632 | 0.19 |
| hsa-miR-1912 | 1.30 | Down | 0.01650331 | 0.03387817 | 0.27 |
| hsa-miR-3190 | 1.30 | Down | 0.0013078 | 0.00421132 | 0.16 |
| hsa-miR-369-3p | 1.30 | Down | 0.01982128 | 0.03954411 | 0.36 |
| hsa-miR-548j | 1.30 | Down | 0.00081984 | 0.00298462 | 0.24 |
| hsa-miR-302c | 1.31 | Down | 0.01695893 | 0.03451945 | 0.28 |
| hsa-miR-367* | 1.31 | Down | 0.00381015 | 0.00997397 | 0.23 |
| hsa-miR-518e* | 1.31 | Down | 1.33E-07 | 1.86E-05 | 0.04 |
| hsa-miR-552 | 1.31 | Down | 0.007829 | 0.0181422 | 0.20 |
| hsa-miR-125b-1* | 1.31 | Down | 0.00355642 | 0.00952947 | 0.21 |
| hsa-miR-520a-3p | 1.31 | Down | 0.00865633 | 0.01986835 | 0.28 |
| hsa-miR-1245 | 1.31 | Down | 0.00105679 | 0.00359727 | 0.21 |
| hsa-miR-450b-5p | 1.32 | Down | 0.00185192 | 0.00555116 | 0.17 |
| hsa-miR-27a* | 1.32 | Down | 5.34E-06 | 9.33E-05 | 0.08 |
| hsa-miR-373 | 1.33 | Down | 0.00092818 | 0.00327034 | 0.20 |
| hsa-miR-548g | 1.33 | Down | 6.97E-05 | 0.00047985 | 0.13 |
| hsa-miR-3929 | 1.33 | Down | 0.01681662 | 0.03428769 | 0.27 |
| hsa-miR-548i | 1.33 | Down | 0.00154829 | 0.00479611 | 0.21 |
| hsa-miR-4311 | 1.33 | Down | 0.00206044 | 0.00605567 | 0.19 |
| hsa-miR-9 | 1.33 | Down | 0.00010342 | 0.00063813 | 0.10 |
| hsa-miR-18a* | 1.33 | Down | 0.00030776 | 0.00140409 | 0.10 |
| hsa-miR-3678-3p | 1.33 | Down | 0.00090371 | 0.0032218 | 0.17 |
| hsa-miR-944 | 1.33 | Down | 0.00090858 | 0.00322963 | 0.20 |
| hsa-miR-449a | 1.33 | Down | 0.00037094 | 0.00159674 | 0.15 |
| hsa-miR-30a* | 1.33 | Down | 6.59E-05 | 0.00047235 | 0.14 |
| hsa-miR-297 | 1.33 | Down | 0.00127402 | 0.00414918 | 0.17 |
| hsa-miR-526b* | 1.34 | Down | 4.13E-05 | 0.00034576 | 0.12 |
| hsa-miR-520g | 1.34 | Down | 0.00078437 | 0.00288161 | 0.18 |
| hsa-miR-1273d | 1.34 | Down | 0.00958019 | 0.02153754 | 0.23 |
| hsa-miR-924 | 1.34 | Down | 0.00128552 | 0.00416826 | 0.24 |
| hsa-miR-2110 | 1.34 | Down | 0.00079047 | 0.00289518 | 0.17 |
| hsa-miR-382 | 1.34 | Down | 6.92E-05 | 0.00047985 | 0.06 |
| hsa-miR-644 | 1.35 | Down | 0.00544369 | 0.01338704 | 0.24 |
| hsa-miR-586 | 1.35 | Down | 0.00034303 | 0.0015195 | 0.18 |
| hsa-miR-3622b-5p | 1.35 | Down | 0.00012975 | 0.00076266 | 0.07 |
| hsa-miR-3150 | 1.35 | Down | 0.00036439 | 0.00157944 | 0.18 |
| hsa-miR-3137 | 1.36 | Down | 0.00779408 | 0.01809609 | 0.25 |
| hsa-miR-520f | 1.36 | Down | 0.00070888 | 0.00263641 | 0.16 |
| hsa-miR-34b* | 1.36 | Down | 0.0026408 | 0.00747716 | 0.22 |
| hsa-miR-302a | 1.36 | Down | 0.00104512 | 0.00356762 | 0.21 |
| hsa-miR-544b | 1.37 | Down | 0.00036206 | 0.00157501 | 0.13 |
| hsa-miR-30b* | 1.37 | Down | 0.00049768 | 0.00198576 | 0.17 |
| hsa-miR-3174 | 1.37 | Down | 1.51E-05 | 0.00020363 | 0.11 |
| hsa-miR-767-5p | 1.37 | Down | 0.00040904 | 0.00169381 | 0.15 |
| hsa-miR-651 | 1.37 | Down | 0.00422345 | 0.01075277 | 0.24 |
| hsa-miR-23a* | 1.37 | Down | 0.02424697 | 0.04727767 | 0.36 |
| hsa-miR-3162 | 1.37 | Down | 0.02202318 | 0.04357625 | 0.28 |
| hsa-miR-3614-3p | 1.38 | Down | 0.0001786 | 0.00094392 | 0.18 |
| hsa-miR-135a | 1.38 | Down | 0.01948214 | 0.03899663 | 0.25 |
| hsa-miR-3915 | 1.38 | Down | 0.00030995 | 0.00140409 | 0.13 |
| hsa-miR-515-3p | 1.39 | Down | 0.00094223 | 0.00329311 | 0.18 |
| hsa-miR-548y | 1.39 | Down | 0.01488086 | 0.03123944 | 0.31 |
| hsa-miR-3150b | 1.39 | Down | 0.00079393 | 0.00289904 | 0.17 |
| hsa-miR-500a | 1.39 | Down | 0.0030448 | 0.00833861 | 0.18 |
| hsa-miR-33b | 1.39 | Down | 0.00027245 | 0.00129253 | 0.19 |
| hsa-miR-1248 | 1.39 | Down | 0.00053008 | 0.00210809 | 0.18 |
| hsa-miR-592 | 1.39 | Down | 2.27E-05 | 0.00023501 | 0.12 |
| hsa-miR-3186-3p | 1.40 | Down | 0.00014007 | 0.00079617 | 0.13 |
| hsa-miR-3916 | 1.40 | Down | 0.00231057 | 0.00667682 | 0.15 |
| hsa-miR-4257 | 1.40 | Down | 7.14E-05 | 0.00048887 | 0.14 |
| hsa-miR-100* | 1.40 | Down | 0.00013078 | 0.000765 | 0.14 |
| hsa-miR-1290 | 1.41 | Down | 0.00038522 | 0.00162872 | 0.13 |
| hsa-miR-1206 | 1.41 | Down | 0.00046796 | 0.00189225 | 0.13 |
| hsa-miR-27b* | 1.42 | Down | 0.00091249 | 0.00323398 | 0.18 |
| hsa-miR-548a-5p | 1.42 | Down | 1.14E-05 | 0.00016175 | 0.11 |
| hsa-miR-601 | 1.42 | Down | 4.86E-05 | 0.00037517 | 0.11 |
| hsa-miR-4273 | 1.42 | Down | 0.00269256 | 0.00758069 | 0.17 |
| hsa-miR-3650 | 1.43 | Down | 0.00061345 | 0.00235415 | 0.16 |
| hsa-miR-34c-5p | 1.43 | Down | 7.33E-05 | 0.00049253 | 0.15 |
| hsa-miR-1205 | 1.43 | Down | 4.05E-05 | 0.00034119 | 0.09 |
| hsa-miR-374a* | 1.43 | Down | 0.00021952 | 0.00110676 | 0.22 |
| hsa-miR-550a* | 1.44 | Down | 0.0174929 | 0.03554629 | 0.27 |
| hsa-miR-374c | 1.44 | Down | 0.00869219 | 0.01991272 | 0.22 |
| hsa-miR-3924 | 1.44 | Down | 0.00347255 | 0.00934022 | 0.24 |
| hsa-miR-649 | 1.44 | Down | 0.00221397 | 0.00645966 | 0.15 |
| hsa-miR-2115* | 1.44 | Down | 0.00014484 | 0.00081558 | 0.16 |
| hsa-miR-325 | 1.45 | Down | 2.77E-05 | 0.00026462 | 0.10 |
| hsa-miR-520c-3p | 1.45 | Down | 0.00149521 | 0.00465562 | 0.22 |
| hsa-miR-548l | 1.45 | Down | 0.00025009 | 0.00121516 | 0.12 |
| hsa-miR-3149 | 1.46 | Down | 6.76E-05 | 0.00047619 | 0.11 |
| hsa-miR-520e | 1.46 | Down | 2.36E-05 | 0.0002409 | 0.10 |
| hsa-miR-580 | 1.46 | Down | 0.00029815 | 0.00137125 | 0.10 |
| hsa-miR-423-5p | 1.46 | Down | 0.00394568 | 0.01022482 | 0.22 |
| hsa-miR-3179 | 1.46 | Down | 9.81E-05 | 0.00061537 | 0.13 |
| hsa-miR-494 | 1.46 | Down | 0.01007727 | 0.02244566 | 0.24 |
| hsa-miR-4307 | 1.47 | Down | 0.00039246 | 0.00164206 | 0.19 |
| hsa-miR-4302 | 1.47 | Down | 0.00031536 | 0.00141795 | 0.09 |
| hsa-miR-876-3p | 1.47 | Down | 0.00017033 | 0.00092978 | 0.17 |
| hsa-miR-3171 | 1.47 | Down | 0.00024416 | 0.00119114 | 0.16 |
| hsa-miR-141 | 1.47 | Down | 2.06E-05 | 0.00023253 | 0.11 |
| hsa-miR-3136 | 1.47 | Down | 0.00013821 | 0.00079308 | 0.14 |
| hsa-miR-3609 | 1.48 | Down | 0.00070185 | 0.0026206 | 0.16 |
| hsa-miR-522 | 1.48 | Down | 0.00025594 | 0.00123364 | 0.16 |
| hsa-miR-520d-3p | 1.48 | Down | 0.00025348 | 0.0012267 | 0.17 |
| hsa-miR-548t | 1.49 | Down | 0.00027678 | 0.0013028 | 0.11 |
| hsa-miR-3936 | 1.49 | Down | 3.39E-05 | 0.00030114 | 0.11 |
| hsa-miR-573 | 1.49 | Down | 0.00297118 | 0.00824947 | 0.21 |
| hsa-miR-1182 | 1.50 | Down | 0.00034425 | 0.0015195 | 0.15 |
| hsa-miR-92a-2* | 1.50 | Down | 2.46E-07 | 2.02E-05 | 0.00 |
| hsa-miR-3917 | 1.50 | Down | 0.00879727 | 0.0200771 | 0.23 |
| hsa-miR-3115 | 1.50 | Down | 2.46E-05 | 0.00024509 | 0.09 |
| hsa-miR-19b-2* | 1.50 | Down | 0.00029974 | 0.00137335 | 0.18 |
| hsa-miR-548b-5p | 1.50 | Down | 8.41E-05 | 0.00054771 | 0.10 |
| hsa-miR-1257 | 1.50 | Down | 7.29E-05 | 0.00049253 | 0.10 |
| hsa-miR-1283 | 1.51 | Down | 0.00038378 | 0.00162872 | 0.03 |
| hsa-miR-3192 | 1.51 | Down | 3.56E-05 | 0.00030879 | 0.09 |
| hsa-miR-3164 | 1.51 | Down | 0.00192778 | 0.0057216 | 0.25 |
| hsa-miR-920 | 1.51 | Down | 0.00012683 | 0.00074918 | 0.13 |
| hsa-miR-93* | 1.51 | Down | 5.20E-05 | 0.00039174 | 0.12 |
| hsa-miR-2278 | 1.52 | Down | 5.84E-05 | 0.00042893 | 0.04 |
| hsa-miR-548d-5p | 1.52 | Down | 0.00383943 | 0.01001411 | 0.15 |
| hsa-miR-99b | 1.52 | Down | 0.00195579 | 0.0057905 | 0.20 |
| hsa-miR-3713 | 1.52 | Down | 1.01E-06 | 3.49E-05 | 0.03 |
| hsa-miR-4316 | 1.52 | Down | 0.00362072 | 0.00967398 | 0.26 |
| hsa-miR-3923 | 1.53 | Down | 0.00033834 | 0.00151002 | 0.15 |
| hsa-miR-3189 | 1.53 | Down | 1.10E-05 | 0.0001603 | 0.10 |
| hsa-miR-1258 | 1.53 | Down | 0.00031207 | 0.00140838 | 0.15 |
| hsa-miR-3622a-5p | 1.53 | Down | 2.17E-05 | 0.00023501 | 0.10 |
| hsa-miR-4278 | 1.54 | Down | 0.00035934 | 0.00156888 | 0.17 |
| hsa-miR-553 | 1.54 | Down | 0.00013203 | 0.0007686 | 0.08 |
| hsa-miR-1252 | 1.54 | Down | 1.58E-05 | 0.00020364 | 0.11 |
| hsa-miR-3617 | 1.54 | Down | 1.56E-06 | 4.58E-05 | 0.07 |
| hsa-miR-32* | 1.54 | Down | 2.14E-05 | 0.00023406 | 0.11 |
| hsa-miR-3606 | 1.55 | Down | 2.21E-05 | 0.00023501 | 0.07 |
| hsa-miR-105* | 1.55 | Down | 0.00023408 | 0.00115128 | 0.19 |
| hsa-miR-147 | 1.55 | Down | 0.0002745 | 0.00129716 | 0.17 |
| hsa-miR-1293 | 1.55 | Down | 0.00023961 | 0.00117368 | 0.13 |
| hsa-miR-30a | 1.55 | Down | 0.00126253 | 0.0041229 | 0.17 |
| hsa-miR-3169 | 1.56 | Down | 6.54E-05 | 0.00047235 | 0.11 |
| hsa-miR-3669 | 1.56 | Down | 3.34E-05 | 0.00030114 | 0.13 |
| hsa-miR-578 | 1.56 | Down | 3.25E-05 | 0.00029676 | 0.11 |
| hsa-miR-2052 | 1.56 | Down | 2.67E-06 | 6.68E-05 | 0.06 |
| hsa-miR-1277 | 1.56 | Down | 1.62E-05 | 0.00020364 | 0.13 |
| hsa-miR-556-5p | 1.56 | Down | 0.00017693 | 0.00093918 | 0.16 |
| hsa-miR-3140 | 1.57 | Down | 0.00013811 | 0.00079308 | 0.13 |
| hsa-miR-92a | 1.57 | Down | 0.00401187 | 0.01032971 | 0.26 |
| hsa-miR-3619 | 1.58 | Down | 0.00440121 | 0.01114172 | 0.16 |
| hsa-miR-3927 | 1.59 | Down | 2.03E-05 | 0.00023253 | 0.08 |
| hsa-miR-1243 | 1.59 | Down | 0.00089509 | 0.00320056 | 0.21 |
| hsa-miR-3613-5p | 1.60 | Down | 0.00018597 | 0.0009659 | 0.16 |
| hsa-miR-3611 | 1.60 | Down | 0.00092603 | 0.00327034 | 0.19 |
| hsa-miR-139-3p | 1.60 | Down | 0.00310498 | 0.00846492 | 0.22 |
| hsa-miR-199a-5p | 1.61 | Down | 2.89E-05 | 0.00027179 | 0.09 |
| hsa-miR-10b* | 1.61 | Down | 6.67E-05 | 0.00047305 | 0.13 |
| hsa-miR-449b | 1.62 | Down | 3.40E-05 | 0.00030114 | 0.13 |
| hsa-miR-519c-3p | 1.62 | Down | 0.00010027 | 0.00062601 | 0.14 |
| hsa-miR-3647-3p | 1.62 | Down | 7.12E-06 | 0.00011749 | 0.06 |
| hsa-miR-29a* | 1.62 | Down | 4.40E-05 | 0.00035681 | 0.16 |
| hsa-miR-3152 | 1.62 | Down | 1.96E-05 | 0.00022757 | 0.11 |
| hsa-miR-3689a-3p | 1.62 | Down | 3.47E-05 | 0.00030297 | 0.10 |
| hsa-miR-3663-5p | 1.63 | Down | 0.00896449 | 0.02038153 | 0.23 |
| hsa-miR-548f | 1.63 | Down | 0.0002308 | 0.00113979 | 0.06 |
| hsa-miR-342-3p | 1.63 | Down | 0.00028237 | 0.00131373 | 0.16 |
| hsa-miR-383 | 1.63 | Down | 4.67E-05 | 0.00036919 | 0.10 |
| hsa-miR-33a | 1.63 | Down | 2.81E-05 | 0.00026634 | 0.12 |
| hsa-miR-1265 | 1.64 | Down | 8.75E-05 | 0.00056678 | 0.13 |
| hsa-miR-941 | 1.64 | Down | 0.00104492 | 0.00356762 | 0.16 |
| hsa-miR-670 | 1.64 | Down | 0.00057613 | 0.00225403 | 0.16 |
| hsa-miR-1306 | 1.65 | Down | 0.00134783 | 0.00427403 | 0.19 |
| hsa-miR-137 | 1.65 | Down | 6.77E-06 | 0.00011331 | 0.12 |
| hsa-miR-3131 | 1.65 | Down | 1.73E-05 | 0.00020897 | 0.10 |
| hsa-miR-576-3p | 1.65 | Down | 3.47E-07 | 2.32E-05 | 0.03 |
| hsa-miR-548m | 1.66 | Down | 2.11E-05 | 0.00023406 | 0.03 |
| hsa-miR-33b* | 1.67 | Down | 0.00510522 | 0.0127103 | 0.20 |
| hsa-miR-551b* | 1.68 | Down | 5.45E-05 | 0.00040523 | 0.13 |
| hsa-miR-3686 | 1.69 | Down | 0.00308177 | 0.00842072 | 0.15 |
| hsa-miR-545* | 1.69 | Down | 2.51E-07 | 2.02E-05 | 0.05 |
| hsa-miR-25 | 1.70 | Down | 2.54E-05 | 0.00025054 | 0.10 |
| hsa-miR-320a | 1.70 | Down | 0.00103865 | 0.00356573 | 0.19 |
| hsa-miR-3668 | 1.71 | Down | 4.61E-07 | 2.46E-05 | 0.06 |
| hsa-miR-616 | 1.71 | Down | 0.0001747 | 0.00093148 | 0.14 |
| hsa-miR-564 | 1.71 | Down | 0.00370647 | 0.00979452 | 0.25 |
| hsa-miR-182 | 1.72 | Down | 0.00328572 | 0.00891732 | 0.23 |
| hsa-miR-1180 | 1.72 | Down | 1.56E-05 | 0.00020364 | 0.10 |
| hsa-miR-187 | 1.72 | Down | 0.00014353 | 0.000812 | 0.15 |
| hsa-miR-590-3p | 1.74 | Down | 0.00011007 | 0.00066876 | 0.12 |
| hsa-miR-3616-3p | 1.74 | Down | 0.02101447 | 0.04167397 | 0.34 |
| hsa-miR-532-3p | 1.74 | Down | 0.00108048 | 0.00366635 | 0.22 |
| hsa-miR-425 | 1.75 | Down | 6.56E-05 | 0.00047235 | 0.11 |
| hsa-miR-885-3p | 1.75 | Down | 0.00205255 | 0.00604725 | 0.20 |
| hsa-miR-3145 | 1.79 | Down | 1.95E-05 | 0.00022757 | 0.07 |
| hsa-miR-1228* | 1.79 | Down | 0.00682564 | 0.01622269 | 0.19 |
| hsa-miR-1321 | 1.79 | Down | 3.92E-05 | 0.00033303 | 0.05 |
| hsa-miR-3673 | 1.80 | Down | 8.94E-06 | 0.00013814 | 0.09 |
| hsa-miR-3662 | 1.81 | Down | 2.97E-06 | 6.88E-05 | 0.06 |
| hsa-miR-1246 | 1.83 | Down | 0.0247498 | 0.04802497 | 0.26 |
| hsa-miR-198 | 1.83 | Down | 3.65E-07 | 2.32E-05 | 0.04 |
| hsa-miR-190 | 1.83 | Down | 3.02E-07 | 2.14E-05 | 0.02 |
| hsa-miR-196a | 1.84 | Down | 2.56E-05 | 0.00025054 | 0.11 |
| hsa-miR-432 | 1.85 | Down | 5.23E-06 | 9.33E-05 | 0.06 |
| hsa-miR-548n | 1.89 | Down | 1.45E-05 | 0.00019809 | 0.12 |
| hsa-miR-1 | 1.89 | Down | 1.61E-05 | 0.00020364 | 0.10 |
| hsa-miR-378 | 1.90 | Down | 0.00161677 | 0.00496991 | 0.20 |
| hsa-miR-3147 | 1.93 | Down | 3.82E-06 | 7.94E-05 | 0.10 |
| hsa-miR-1244 | 1.95 | Down | 4.71E-06 | 8.73E-05 | 0.07 |
| hsa-miR-922 | 1.95 | Down | 0.00049278 | 0.00197276 | 0.23 |
| hsa-miR-30b | 1.96 | Down | 0.00121711 | 0.00400715 | 0.17 |
| hsa-miR-30d | 2.01 | Down | 1.83E-05 | 0.00021779 | 0.10 |
| hsa-miR-1262 | 2.03 | Down | 2.40E-07 | 2.02E-05 | 0.04 |
| hsa-miR-623 | 2.04 | Down | 0.00038929 | 0.0016402 | 0.13 |
| hsa-miR-190b | 2.05 | Down | 2.39E-05 | 0.0002409 | 0.08 |
| hsa-miR-140-3p | 2.06 | Down | 3.82E-06 | 7.94E-05 | 0.05 |
| hsa-miR-4271 | 2.06 | Down | 9.38E-06 | 0.00014308 | 0.10 |
| hsa-miR-30c | 2.07 | Down | 0.00027904 | 0.00130551 | 0.16 |
| hsa-miR-186 | 2.08 | Down | 0.02538976 | 0.04902991 | 0.33 |
| hsa-miR-3129 | 2.08 | Down | 2.13E-05 | 0.00023406 | 0.09 |
| hsa-miR-620 | 2.11 | Down | 1.09E-06 | 3.54E-05 | 0.06 |
| hsa-miR-222 | 2.12 | Down | 0.00034066 | 0.00151475 | 0.15 |
| hsa-miR-142-5p | 2.14 | Down | 0.00133586 | 0.00424724 | 0.21 |
| hsa-miR-191 | 2.15 | Down | 5.11E-07 | 2.47E-05 | 0.06 |
| hsa-miR-3689b* | 2.15 | Down | 2.91E-06 | 6.88E-05 | 0.03 |
| hsa-miR-1307 | 2.24 | Down | 0.00131992 | 0.00421885 | 0.23 |
| hsa-miR-151-3p | 2.28 | Down | 1.70E-07 | 1.86E-05 | 0.04 |
| hsa-miR-595 | 2.36 | Down | 0.00040326 | 0.0016756 | 0.17 |
| hsa-miR-26a | 2.38 | Down | 0.0012868 | 0.00416826 | 0.19 |
| hsa-miR-339-5p | 2.51 | Down | 1.27E-07 | 1.86E-05 | 0.03 |
| hsa-miR-181b | 2.62 | Down | 0.00065751 | 0.00251525 | 0.17 |
| hsa-miR-3201 | 2.67 | Down | 0.00385859 | 0.01004234 | 0.14 |
| hsa-miR-194 | 2.70 | Down | 1.62E-07 | 1.86E-05 | 0.06 |
| hsa-miR-206 | 2.88 | Down | 9.50E-07 | 3.47E-05 | 0.07 |
| hsa-miR-3653 | 3.99 | Down | 0.00518476 | 0.01282883 | 0.23 |
| hsa-miR-181d | 5.34 | Down | 1.54E-05 | 0.00020363 | 0.06 |
|  |  |  |  |  |  |
| **C) TOF-HF patients (n=3) compared to matched healthy controls (n=3)** | | | | | |
| **miRNA** | **Fold Change** | **Regulation** | **Ttest** | **Ttest Adj** |  |
| hsa-miR-625 | 11.77 | Up | 0.00086765 | 0.04021214 |  |
| hsa-miR-183* | 6.16 | Up | 0.04180307 | 0.15081645 |  |
| hsa-miR-183 | 6.08 | Up | 0.01658446 | 0.09316599 |  |
| hsa-miR-505* | 5.66 | Up | 0.0209635 | 0.10569463 |  |
| hsa-miR-214 | 4.60 | Up | 0.00060924 | 0.03273548 |  |
| hsa-miR-30e* | 4.59 | Up | 0.01515596 | 0.08983755 |  |
| hsa-let-7d | 4.44 | Up | 0.003245 | 0.06206702 |  |
| hsa-miR-340* | 3.94 | Up | 0.04510757 | 0.15399986 |  |
| hsa-miR-28-5p | 3.81 | Up | 0.00964018 | 0.08100654 |  |
| hsa-miR-15b | 3.81 | Up | 3.79E-05 | 0.01568949 |  |
| hsa-miR-151-5p | 3.77 | Up | 0.00097558 | 0.04059976 |  |
| hsa-let-7a | 3.51 | Up | 0.0439511 | 0.15306669 |  |
| hsa-miR-1271 | 3.34 | Up | 0.00132817 | 0.04422652 |  |
| hsa-miR-192 | 3.30 | Up | 0.00165518 | 0.04422652 |  |
| hsa-miR-20b | 3.03 | Up | 0.01170214 | 0.08367891 |  |
| hsa-miR-23b | 3.02 | Up | 0.00101268 | 0.04059976 |  |
| hsa-miR-148b | 2.95 | Up | 0.01452024 | 0.08972761 |  |
| hsa-miR-339-3p | 2.89 | Up | 0.00630403 | 0.07110519 |  |
| hsa-let-7g | 2.85 | Up | 0.01685164 | 0.09316599 |  |
| hsa-miR-660 | 2.71 | Up | 0.01016074 | 0.08108402 |  |
| hsa-miR-20a | 2.69 | Up | 0.03592384 | 0.13569977 |  |
| hsa-miR-502-3p | 2.60 | Up | 0.00062483 | 0.03273548 |  |
| hsa-miR-7-1* | 2.59 | Up | 0.00249467 | 0.05465586 |  |
| hsa-miR-3677 | 2.55 | Up | 0.01432991 | 0.0896585 |  |
| hsa-miR-1274b | 2.49 | Up | 0.02751567 | 0.11926758 |  |
| hsa-miR-218-2* | 2.49 | Up | 0.00433459 | 0.06440912 |  |
| hsa-miR-18a | 2.38 | Up | 0.00647465 | 0.07157759 |  |
| hsa-miR-330-3p | 2.36 | Up | 0.00405633 | 0.06431413 |  |
| hsa-miR-1274a | 2.32 | Up | 0.00430173 | 0.06440912 |  |
| hsa-miR-29a | 2.31 | Up | 0.00592239 | 0.07065823 |  |
| hsa-miR-138-1* | 2.30 | Up | 0.00078648 | 0.03948767 |  |
| hsa-miR-744 | 2.28 | Up | 5.91E-05 | 0.01568949 |  |
| hsa-miR-132 | 2.19 | Up | 0.0079738 | 0.07506587 |  |
| hsa-miR-1181 | 2.19 | Up | 0.00028805 | 0.02454494 |  |
| hsa-miR-29c | 2.17 | Up | 0.02664666 | 0.1176162 |  |
| hsa-miR-3195 | 2.16 | Up | 0.02933909 | 0.12142103 |  |
| hsa-miR-24 | 2.13 | Up | 0.00711577 | 0.07313614 |  |
| hsa-miR-3125 | 2.10 | Up | 0.00459799 | 0.06440912 |  |
| hsa-miR-3127 | 2.05 | Up | 0.00465029 | 0.06440912 |  |
| hsa-miR-192* | 2.04 | Up | 0.00016023 | 0.02228672 |  |
| hsa-miR-1288 | 2.03 | Up | 0.00404584 | 0.06431413 |  |
| hsa-miR-200c | 2.00 | Up | 0.00084231 | 0.04021214 |  |
| hsa-miR-532-5p | 1.98 | Up | 0.00455635 | 0.06440912 |  |
| hsa-miR-629 | 1.96 | Up | 0.00860724 | 0.07758951 |  |
| hsa-miR-718 | 1.95 | Up | 0.02479937 | 0.11405818 |  |
| hsa-miR-27a | 1.91 | Up | 0.00499834 | 0.06844312 |  |
| hsa-miR-4291 | 1.91 | Up | 0.03785097 | 0.14077291 |  |
| hsa-miR-27b | 1.91 | Up | 0.03088479 | 0.12476023 |  |
| hsa-miR-193b | 1.91 | Up | 0.00301498 | 0.06206702 |  |
| hsa-miR-378b | 1.90 | Up | 0.00036806 | 0.02463953 |  |
| hsa-miR-363 | 1.89 | Up | 0.00061401 | 0.03273548 |  |
| hsa-miR-3918 | 1.87 | Up | 0.02704271 | 0.11846786 |  |
| hsa-miR-3124 | 1.87 | Up | 0.0013284 | 0.04422652 |  |
| hsa-miR-487b | 1.86 | Up | 5.96E-05 | 0.01568949 |  |
| hsa-miR-542-5p | 1.86 | Up | 0.00019848 | 0.02391692 |  |
| hsa-miR-362-3p | 1.84 | Up | 0.00220647 | 0.05060254 |  |
| hsa-miR-3198 | 1.83 | Up | 0.00317908 | 0.06206702 |  |
| hsa-miR-148a | 1.82 | Up | 0.02350928 | 0.11286326 |  |
| hsa-miR-3690 | 1.79 | Up | 0.01231669 | 0.0841462 |  |
| hsa-miR-3200-3p | 1.78 | Up | 0.0220153 | 0.10827934 |  |
| hsa-miR-17* | 1.77 | Up | 0.03831471 | 0.14205914 |  |
| hsa-miR-3910 | 1.77 | Up | 0.01862867 | 0.09854126 |  |
| hsa-miR-323-3p | 1.73 | Up | 0.01122503 | 0.08349482 |  |
| hsa-miR-502-5p | 1.73 | Up | 0.01756617 | 0.09534791 |  |
| hsa-miR-3163 | 1.73 | Up | 0.00062358 | 0.03273548 |  |
| hsa-miR-921 | 1.72 | Up | 0.00342905 | 0.06254319 |  |
| hsa-miR-193a-3p | 1.71 | Up | 0.00193792 | 0.04670385 |  |
| hsa-miR-362-5p | 1.70 | Up | 0.02149438 | 0.1070278 |  |
| hsa-miR-17 | 1.69 | Up | 0.03131658 | 0.12495523 |  |
| hsa-miR-1305 | 1.68 | Up | 0.01700956 | 0.09316599 |  |
| hsa-miR-373* | 1.67 | Up | 0.03202066 | 0.12609442 |  |
| hsa-miR-22* | 1.65 | Up | 0.02798308 | 0.11999862 |  |
| hsa-miR-744* | 1.65 | Up | 0.01100958 | 0.08343736 |  |
| hsa-miR-590-5p | 1.64 | Up | 0.01341956 | 0.08788352 |  |
| hsa-miR-641 | 1.64 | Up | 0.00226378 | 0.05060254 |  |
| hsa-miR-887 | 1.64 | Up | 0.01602766 | 0.09240829 |  |
| hsa-miR-624* | 1.62 | Up | 0.00176172 | 0.04422652 |  |
| hsa-miR-129-3p | 1.62 | Up | 0.02451151 | 0.11405818 |  |
| hsa-miR-1226* | 1.62 | Up | 0.00559411 | 0.06884254 |  |
| hsa-miR-2355-5p | 1.62 | Up | 0.03130498 | 0.12495523 |  |
| hsa-miR-3610 | 1.61 | Up | 0.00761977 | 0.07313614 |  |
| hsa-miR-500a* | 1.60 | Up | 0.01697794 | 0.09316599 |  |
| hsa-miR-22 | 1.60 | Up | 0.01821029 | 0.0979616 |  |
| hsa-miR-634 | 1.59 | Up | 0.02477691 | 0.11405818 |  |
| hsa-miR-363* | 1.58 | Up | 0.00100198 | 0.04059976 |  |
| hsa-miR-4258 | 1.58 | Up | 0.00366448 | 0.06310873 |  |
| hsa-miR-409-5p | 1.55 | Up | 0.00323834 | 0.06206702 |  |
| hsa-miR-2117 | 1.55 | Up | 0.0100838 | 0.08100654 |  |
| hsa-miR-892b | 1.55 | Up | 0.00916697 | 0.07946903 |  |
| hsa-miR-3126-5p | 1.55 | Up | 0.04328995 | 0.15293946 |  |
| hsa-miR-501-3p | 1.55 | Up | 0.04051195 | 0.14793001 |  |
| hsa-miR-20b* | 1.54 | Up | 0.00544363 | 0.06856746 |  |
| hsa-miR-4262 | 1.54 | Up | 0.045986 | 0.15565486 |  |
| hsa-miR-196b* | 1.54 | Up | 0.00764743 | 0.07313614 |  |
| hsa-miR-647 | 1.54 | Up | 0.00051553 | 0.03269563 |  |
| hsa-miR-593* | 1.53 | Up | 0.01182666 | 0.08367891 |  |
| hsa-miR-891a | 1.53 | Up | 0.02136423 | 0.10682116 |  |
| hsa-miR-3648 | 1.52 | Up | 0.02691184 | 0.11835319 |  |
| hsa-miR-1208 | 1.52 | Up | 0.00565594 | 0.06884254 |  |
| hsa-miR-3161 | 1.52 | Up | 0.03179192 | 0.12561045 |  |
| hsa-miR-1224-3p | 1.51 | Up | 0.01375174 | 0.08874412 |  |
| hsa-miR-449b* | 1.50 | Up | 0.0046417 | 0.06440912 |  |
| hsa-miR-4252 | 1.50 | Up | 0.00426899 | 0.06440912 |  |
| hsa-miR-148a* | 1.50 | Up | 0.00527137 | 0.06856746 |  |
| hsa-miR-1273c | 1.48 | Up | 0.00452261 | 0.06440912 |  |
| hsa-miR-3658 | 1.48 | Up | 0.04041737 | 0.14793001 |  |
| hsa-miR-589* | 1.48 | Up | 0.00854579 | 0.07758951 |  |
| hsa-miR-621 | 1.47 | Up | 0.00342703 | 0.06254319 |  |
| hsa-miR-3157 | 1.47 | Up | 0.01461327 | 0.08980768 |  |
| hsa-miR-299-5p | 1.46 | Up | 0.02951195 | 0.12142103 |  |
| hsa-miR-18b | 1.46 | Up | 0.03977607 | 0.14702504 |  |
| hsa-miR-943 | 1.45 | Up | 0.00831317 | 0.07718403 |  |
| hsa-miR-3120 | 1.44 | Up | 0.01236006 | 0.0841462 |  |
| hsa-miR-640 | 1.44 | Up | 0.00545375 | 0.06856746 |  |
| hsa-miR-33a* | 1.43 | Up | 0.00943047 | 0.0805937 |  |
| hsa-miR-770-5p | 1.43 | Up | 0.02399482 | 0.1138337 |  |
| hsa-miR-4285 | 1.43 | Up | 0.01719252 | 0.09374204 |  |
| hsa-miR-758 | 1.42 | Up | 0.00572376 | 0.06897136 |  |
| hsa-miR-636 | 1.41 | Up | 0.01201365 | 0.08367891 |  |
| hsa-miR-3939 | 1.40 | Up | 0.01485334 | 0.08980768 |  |
| hsa-miR-129* | 1.40 | Up | 0.01325397 | 0.08775293 |  |
| hsa-miR-1233 | 1.40 | Up | 0.02453059 | 0.11405818 |  |
| hsa-miR-491-5p | 1.40 | Up | 0.00370238 | 0.06310873 |  |
| hsa-miR-335 | 1.39 | Up | 0.0216377 | 0.10729806 |  |
| hsa-miR-3685 | 1.39 | Up | 0.00385751 | 0.06310873 |  |
| hsa-let-7c* | 1.38 | Up | 0.04160179 | 0.15081645 |  |
| hsa-miR-148b* | 1.38 | Up | 0.00632505 | 0.07110519 |  |
| hsa-miR-3622b-3p | 1.38 | Up | 0.00725919 | 0.07313614 |  |
| hsa-miR-3663-3p | 1.38 | Up | 0.02713455 | 0.11846786 |  |
| hsa-miR-654-3p | 1.37 | Up | 0.04387065 | 0.15306669 |  |
| hsa-miR-3911 | 1.37 | Up | 0.02474372 | 0.11405818 |  |
| hsa-miR-769-5p | 1.36 | Up | 0.03118345 | 0.12495523 |  |
| hsa-miR-4317 | 1.36 | Up | 0.00985928 | 0.08100654 |  |
| hsa-miR-421 | 1.36 | Up | 0.00332104 | 0.06252892 |  |
| hsa-miR-34b | 1.36 | Up | 0.01520901 | 0.08983755 |  |
| hsa-miR-1229 | 1.35 | Up | 0.00750229 | 0.07313614 |  |
| hsa-miR-654-5p | 1.35 | Up | 0.00832691 | 0.07718403 |  |
| hsa-miR-2115 | 1.35 | Up | 0.04419972 | 0.15341261 |  |
| hsa-miR-4279 | 1.35 | Up | 0.0099332 | 0.08100654 |  |
| hsa-miR-300 | 1.34 | Up | 0.03092212 | 0.12476023 |  |
| hsa-miR-662 | 1.34 | Up | 0.04523037 | 0.15399986 |  |
| hsa-miR-676* | 1.33 | Up | 0.0292346 | 0.12142103 |  |
| hsa-miR-3675-3p | 1.33 | Up | 0.03770751 | 0.14067351 |  |
| hsa-miR-424 | 1.33 | Up | 0.02981049 | 0.12142103 |  |
| hsa-miR-376a | 1.33 | Up | 0.00972825 | 0.08100654 |  |
| hsa-miR-3156 | 1.32 | Up | 0.02304151 | 0.11195572 |  |
| hsa-miR-193b* | 1.32 | Up | 0.04246007 | 0.15182311 |  |
| hsa-miR-582-3p | 1.31 | Up | 0.01984292 | 0.10262112 |  |
| hsa-miR-3943 | 1.31 | Up | 0.0071205 | 0.07313614 |  |
| hsa-miR-885-5p | 1.30 | Up | 0.00663968 | 0.07273468 |  |
| hsa-miR-224* | 1.30 | Up | 0.04524145 | 0.15399986 |  |
| hsa-miR-212 | 1.30 | Up | 0.03401831 | 0.13138483 |  |
| hsa-miR-4301 | 1.29 | Up | 0.02536854 | 0.11420992 |  |
| hsa-miR-3907 | 1.29 | Up | 0.04718008 | 0.15798924 |  |
| hsa-miR-3133 | 1.29 | Up | 0.01384556 | 0.08874412 |  |
| hsa-miR-410 | 1.29 | Up | 0.03722415 | 0.13973553 |  |
| hsa-miR-1286 | 1.28 | Up | 0.04230319 | 0.15171233 |  |
| hsa-miR-4293 | 1.27 | Up | 0.03588182 | 0.13569977 |  |
| hsa-miR-346 | 1.26 | Up | 0.0309571 | 0.12476023 |  |
| hsa-miR-411* | 1.26 | Up | 0.01507709 | 0.08983755 |  |
| hsa-miR-497 | 1.26 | Up | 0.04733122 | 0.15798924 |  |
| hsa-miR-600 | 1.25 | Up | 0.02791483 | 0.11999862 |  |
| hsa-miR-4275 | 1.24 | Up | 0.02509621 | 0.11420992 |  |
| hsa-miR-124* | 1.23 | Up | 0.04757185 | 0.15835381 |  |
| hsa-miR-665 | 1.23 | Up | 0.02935821 | 0.12142103 |  |
| hsa-miR-4254 | 1.22 | Up | 0.02939644 | 0.12142103 |  |
| hsa-miR-657 | 1.22 | Up | 0.01338276 | 0.08788352 |  |
| hsa-miR-122* | 1.22 | Up | 0.02082857 | 0.10569463 |  |
| hsa-miR-487a | 1.22 | Up | 0.04488737 | 0.15399986 |  |
| hsa-miR-518a-5p | 1.22 | Up | 0.02505913 | 0.11420992 |  |
| hsa-miR-3684 | 1.15 | Up | 0.04262077 | 0.15194684 |  |
| hsa-miR-449a | 1.18 | Down | 0.04389463 | 0.15306669 |  |
| hsa-miR-3924 | 1.19 | Down | 0.04430505 | 0.15341261 |  |
| hsa-miR-497* | 1.20 | Down | 0.03373709 | 0.13071768 |  |
| hsa-miR-509-3p | 1.21 | Down | 0.02896327 | 0.12142103 |  |
| hsa-miR-522 | 1.21 | Down | 0.04046363 | 0.14793001 |  |
| hsa-miR-3927 | 1.22 | Down | 0.03691484 | 0.13900746 |  |
| hsa-miR-135b | 1.23 | Down | 0.01908563 | 0.09955926 |  |
| hsa-miR-1537 | 1.23 | Down | 0.02275901 | 0.11103081 |  |
| hsa-miR-520b | 1.23 | Down | 0.02828167 | 0.12084896 |  |
| hsa-miR-302b | 1.23 | Down | 0.03162928 | 0.12561045 |  |
| hsa-miR-33a | 1.23 | Down | 0.04340688 | 0.15293946 |  |
| hsa-miR-1258 | 1.24 | Down | 0.0247182 | 0.11405818 |  |
| hsa-miR-3136 | 1.24 | Down | 0.03179352 | 0.12561045 |  |
| hsa-miR-3607-3p | 1.24 | Down | 0.02963908 | 0.12142103 |  |
| hsa-miR-4315 | 1.25 | Down | 0.04806919 | 0.15956851 |  |
| hsa-miR-137 | 1.25 | Down | 0.01429289 | 0.0896585 |  |
| hsa-miR-374b* | 1.25 | Down | 0.02846182 | 0.12118901 |  |
| hsa-miR-132* | 1.25 | Down | 0.01420242 | 0.0896585 |  |
| hsa-miR-187 | 1.26 | Down | 0.02254285 | 0.11042329 |  |
| hsa-miR-302c | 1.26 | Down | 0.03542639 | 0.13533371 |  |
| hsa-miR-129-5p | 1.26 | Down | 0.01587188 | 0.09215584 |  |
| hsa-miR-1278 | 1.26 | Down | 0.01479585 | 0.08980768 |  |
| hsa-miR-3189 | 1.27 | Down | 0.01892497 | 0.09915038 |  |
| hsa-miR-100* | 1.27 | Down | 0.03283643 | 0.1284672 |  |
| hsa-miR-548c-3p | 1.27 | Down | 0.01383903 | 0.08874412 |  |
| hsa-miR-3689a-5p | 1.28 | Down | 0.03414009 | 0.13143389 |  |
| hsa-miR-3675-5p | 1.28 | Down | 0.04359217 | 0.15306669 |  |
| hsa-miR-520e | 1.28 | Down | 0.01216254 | 0.08374781 |  |
| hsa-miR-99a* | 1.28 | Down | 0.04193269 | 0.1508325 |  |
| hsa-miR-1265 | 1.28 | Down | 0.03735263 | 0.13978237 |  |
| hsa-miR-520g | 1.29 | Down | 0.0159074 | 0.09215584 |  |
| hsa-miR-876-3p | 1.29 | Down | 0.00866748 | 0.07758951 |  |
| hsa-miR-548t | 1.29 | Down | 0.01054877 | 0.08245791 |  |
| hsa-miR-520c-3p | 1.29 | Down | 0.01120729 | 0.08349482 |  |
| hsa-miR-548x | 1.29 | Down | 0.02928259 | 0.12142103 |  |
| hsa-miR-29a* | 1.29 | Down | 0.01774124 | 0.09586632 |  |
| hsa-miR-552 | 1.30 | Down | 0.01063409 | 0.08245791 |  |
| hsa-miR-1262 | 1.30 | Down | 0.02071729 | 0.10569463 |  |
| hsa-miR-553 | 1.30 | Down | 0.02635789 | 0.1172002 |  |
| hsa-miR-135a | 1.30 | Down | 0.04596058 | 0.15565486 |  |
| hsa-miR-325 | 1.30 | Down | 0.00634798 | 0.07110519 |  |
| hsa-miR-514 | 1.31 | Down | 0.04017661 | 0.14793001 |  |
| hsa-miR-1825 | 1.31 | Down | 0.01262767 | 0.08500751 |  |
| hsa-miR-196b | 1.31 | Down | 0.01436024 | 0.0896585 |  |
| hsa-miR-1248 | 1.32 | Down | 0.04704395 | 0.15798924 |  |
| hsa-miR-1205 | 1.33 | Down | 0.01484725 | 0.08980768 |  |
| hsa-miR-517c | 1.33 | Down | 0.00899526 | 0.0785456 |  |
| hsa-miR-519c-3p | 1.34 | Down | 0.01628879 | 0.09316599 |  |
| hsa-miR-580 | 1.34 | Down | 0.01683883 | 0.09316599 |  |
| hsa-miR-3619 | 1.34 | Down | 0.01006453 | 0.08100654 |  |
| hsa-miR-548m | 1.34 | Down | 0.01042308 | 0.08245791 |  |
| hsa-miR-1321 | 1.34 | Down | 0.01938032 | 0.10066073 |  |
| hsa-miR-676 | 1.34 | Down | 0.01134071 | 0.08367891 |  |
| hsa-miR-4278 | 1.35 | Down | 0.01115797 | 0.08349482 |  |
| hsa-miR-147b | 1.36 | Down | 0.00968668 | 0.08100654 |  |
| hsa-miR-520f | 1.36 | Down | 0.02982624 | 0.12142103 |  |
| hsa-miR-190 | 1.36 | Down | 0.00527976 | 0.06856746 |  |
| hsa-miR-3186-3p | 1.36 | Down | 0.02581792 | 0.11565276 |  |
| hsa-miR-1283 | 1.36 | Down | 0.0145183 | 0.08972761 |  |
| hsa-miR-367* | 1.36 | Down | 0.02336928 | 0.11276363 |  |
| hsa-miR-3171 | 1.36 | Down | 0.00727611 | 0.07313614 |  |
| hsa-miR-383 | 1.37 | Down | 0.00385242 | 0.06310873 |  |
| hsa-miR-1182 | 1.37 | Down | 0.01848446 | 0.09854126 |  |
| hsa-miR-510 | 1.37 | Down | 0.00841567 | 0.07741135 |  |
| hsa-let-7f-1* | 1.37 | Down | 0.02033973 | 0.10429522 |  |
| hsa-miR-515-3p | 1.37 | Down | 0.00447714 | 0.06440912 |  |
| hsa-miR-3669 | 1.38 | Down | 0.04651089 | 0.15699055 |  |
| hsa-miR-3923 | 1.38 | Down | 0.01284293 | 0.08597626 |  |
| hsa-miR-1 | 1.38 | Down | 0.01058209 | 0.08245791 |  |
| hsa-miR-3617 | 1.38 | Down | 0.00753649 | 0.07313614 |  |
| hsa-miR-449b | 1.38 | Down | 0.00309593 | 0.06206702 |  |
| hsa-miR-885-3p | 1.38 | Down | 0.04173108 | 0.15081645 |  |
| hsa-miR-3655 | 1.39 | Down | 0.02393857 | 0.1138337 |  |
| hsa-miR-3662 | 1.39 | Down | 0.00752229 | 0.07313614 |  |
| hsa-miR-548c-5p | 1.39 | Down | 0.04316347 | 0.15293946 |  |
| hsa-miR-658 | 1.39 | Down | 0.03549 | 0.13533371 |  |
| hsa-miR-219-1-3p | 1.39 | Down | 0.00387556 | 0.06310873 |  |
| hsa-miR-3647-3p | 1.39 | Down | 0.01006913 | 0.08100654 |  |
| hsa-miR-3609 | 1.39 | Down | 0.0327221 | 0.12843692 |  |
| hsa-miR-3621 | 1.40 | Down | 0.00887672 | 0.0785456 |  |
| hsa-miR-3193 | 1.40 | Down | 0.01864515 | 0.09854126 |  |
| hsa-miR-448 | 1.40 | Down | 0.00611967 | 0.07090577 |  |
| hsa-miR-4311 | 1.41 | Down | 0.01156809 | 0.08367891 |  |
| hsa-miR-3689a-3p | 1.41 | Down | 0.00261457 | 0.05625998 |  |
| hsa-miR-105* | 1.41 | Down | 0.00213146 | 0.05036104 |  |
| hsa-miR-626 | 1.42 | Down | 0.01829647 | 0.09798776 |  |
| hsa-miR-302a | 1.42 | Down | 0.01188335 | 0.08367891 |  |
| hsa-miR-141 | 1.43 | Down | 0.00699317 | 0.07313614 |  |
| hsa-miR-373 | 1.43 | Down | 0.00274837 | 0.05810142 |  |
| hsa-miR-320c | 1.43 | Down | 0.0278955 | 0.11999862 |  |
| hsa-miR-941 | 1.43 | Down | 0.01412576 | 0.0896585 |  |
| hsa-miR-3144-5p | 1.44 | Down | 0.01163996 | 0.08367891 |  |
| hsa-miR-512-3p | 1.44 | Down | 0.03449187 | 0.1323653 |  |
| hsa-miR-320e | 1.45 | Down | 0.00604168 | 0.07090577 |  |
| hsa-miR-2278 | 1.45 | Down | 0.00157126 | 0.04422652 |  |
| hsa-miR-342-3p | 1.45 | Down | 0.04116406 | 0.14985709 |  |
| hsa-miR-1267 | 1.45 | Down | 0.00741826 | 0.07313614 |  |
| hsa-miR-4307 | 1.46 | Down | 0.02185178 | 0.10791554 |  |
| hsa-miR-34c-5p | 1.46 | Down | 0.02521385 | 0.11420992 |  |
| hsa-miR-3613-5p | 1.46 | Down | 0.0170061 | 0.09316599 |  |
| hsa-miR-3148 | 1.47 | Down | 0.01181136 | 0.08367891 |  |
| hsa-miR-4313 | 1.47 | Down | 0.0043413 | 0.06440912 |  |
| hsa-miR-200a | 1.47 | Down | 0.00896971 | 0.0785456 |  |
| hsa-miR-3135 | 1.47 | Down | 0.0429324 | 0.15260633 |  |
| hsa-miR-548b-5p | 1.48 | Down | 0.00170213 | 0.04422652 |  |
| hsa-miR-1293 | 1.49 | Down | 0.00121205 | 0.04422652 |  |
| hsa-miR-4312 | 1.49 | Down | 0.00321103 | 0.06206702 |  |
| hsa-miR-151-3p | 1.50 | Down | 0.00606244 | 0.07090577 |  |
| hsa-miR-3129 | 1.50 | Down | 0.00169708 | 0.04422652 |  |
| hsa-miR-3185 | 1.51 | Down | 0.02095155 | 0.10569463 |  |
| hsa-miR-548n | 1.52 | Down | 0.0009495 | 0.04059976 |  |
| hsa-miR-520d-3p | 1.52 | Down | 0.00173518 | 0.04422652 |  |
| hsa-miR-620 | 1.53 | Down | 0.00158755 | 0.04422652 |  |
| hsa-miR-23c | 1.53 | Down | 0.01089245 | 0.08307219 |  |
| hsa-miR-1294 | 1.53 | Down | 0.01553954 | 0.09134218 |  |
| hsa-miR-591 | 1.53 | Down | 0.00170537 | 0.04422652 |  |
| hsa-miR-3145 | 1.53 | Down | 0.01195933 | 0.08367891 |  |
| hsa-miR-190b | 1.54 | Down | 0.00168886 | 0.04422652 |  |
| hsa-miR-205* | 1.54 | Down | 0.02002028 | 0.10309588 |  |
| hsa-miR-425* | 1.55 | Down | 0.00976498 | 0.08100654 |  |
| hsa-miR-297 | 1.56 | Down | 0.02426075 | 0.11405818 |  |
| hsa-miR-3192 | 1.58 | Down | 0.0149786 | 0.08980768 |  |
| hsa-miR-140-3p | 1.58 | Down | 0.02426966 | 0.11405818 |  |
| hsa-miR-639 | 1.59 | Down | 0.02378071 | 0.11371332 |  |
| hsa-miR-1246 | 1.61 | Down | 0.01254023 | 0.08489313 |  |
| hsa-miR-3115 | 1.61 | Down | 0.02966458 | 0.12142103 |  |
| hsa-miR-1471 | 1.64 | Down | 0.01210716 | 0.08374781 |  |
| hsa-miR-711 | 1.65 | Down | 0.0473169 | 0.15798924 |  |
| hsa-miR-3611 | 1.65 | Down | 0.01183023 | 0.08367891 |  |
| hsa-miR-648 | 1.65 | Down | 0.03370141 | 0.13071768 |  |
| hsa-miR-3650 | 1.65 | Down | 0.00157501 | 0.04422652 |  |
| hsa-let-7c | 1.65 | Down | 0.03352165 | 0.13071768 |  |
| hsa-miR-3616-3p | 1.65 | Down | 0.02110256 | 0.10595241 |  |
| hsa-miR-146a | 1.66 | Down | 0.02661016 | 0.1176162 |  |
| hsa-miR-3689b* | 1.67 | Down | 0.01079075 | 0.08282076 |  |
| hsa-miR-1238 | 1.68 | Down | 0.00747171 | 0.07313614 |  |
| hsa-miR-1273e | 1.68 | Down | 0.04504559 | 0.15399986 |  |
| hsa-miR-3929 | 1.70 | Down | 0.00637291 | 0.07110519 |  |
| hsa-miR-339-5p | 1.73 | Down | 0.00131882 | 0.04422652 |  |
| hsa-miR-125b-1* | 1.75 | Down | 0.02725528 | 0.11856538 |  |
| hsa-miR-32* | 1.80 | Down | 0.00030554 | 0.02454494 |  |
| hsa-miR-1180 | 1.81 | Down | 0.01649941 | 0.09316599 |  |
| hsa-miR-198 | 1.81 | Down | 0.04513265 | 0.15399986 |  |
| hsa-miR-320b | 1.81 | Down | 0.01877634 | 0.09880126 |  |
| hsa-miR-548d-5p | 1.82 | Down | 0.01382481 | 0.08874412 |  |
| hsa-miR-196a | 1.83 | Down | 0.00029426 | 0.02454494 |  |
| hsa-miR-1244 | 1.85 | Down | 0.00035849 | 0.02463953 |  |
| hsa-miR-3201 | 1.87 | Down | 0.03576633 | 0.13569977 |  |
| hsa-miR-139-3p | 1.87 | Down | 0.00422831 | 0.06440912 |  |
| hsa-miR-191 | 1.87 | Down | 0.00226767 | 0.05060254 |  |
| hsa-miR-1275 | 1.91 | Down | 0.01685732 | 0.09316599 |  |
| hsa-miR-93* | 1.92 | Down | 0.0086926 | 0.07758951 |  |
| hsa-miR-3149 | 1.94 | Down | 0.00022769 | 0.02454494 |  |
| hsa-miR-30b | 1.96 | Down | 0.00924008 | 0.07953072 |  |
| hsa-miR-3917 | 1.99 | Down | 0.015678 | 0.09170867 |  |
| hsa-miR-875-3p | 1.99 | Down | 0.00536616 | 0.06856746 |  |
| hsa-miR-3713 | 1.99 | Down | 0.005339 | 0.06856746 |  |
| hsa-miR-920 | 2.01 | Down | 0.02973416 | 0.12142103 |  |
| hsa-miR-874 | 2.02 | Down | 0.02339494 | 0.11276363 |  |
| hsa-miR-3686 | 2.08 | Down | 0.00732764 | 0.07313614 |  |
| hsa-miR-425 | 2.08 | Down | 0.00546264 | 0.06856746 |  |
| hsa-miR-3147 | 2.14 | Down | 0.000301 | 0.02454494 |  |
| hsa-miR-186 | 2.15 | Down | 0.00716873 | 0.07313614 |  |
| hsa-miR-1306 | 2.19 | Down | 0.00564163 | 0.06884254 |  |
| hsa-miR-145 | 2.25 | Down | 0.01067505 | 0.08245791 |  |
| hsa-miR-4316 | 2.25 | Down | 0.01319953 | 0.08775293 |  |
| hsa-miR-30c | 2.27 | Down | 0.00136829 | 0.04422652 |  |
| hsa-miR-940 | 2.28 | Down | 0.00181558 | 0.04464837 |  |
| hsa-miR-452* | 2.35 | Down | 0.00347751 | 0.06254319 |  |
| hsa-miR-3191 | 2.38 | Down | 0.00758278 | 0.07313614 |  |
| hsa-miR-670 | 2.41 | Down | 0.00133179 | 0.04422652 |  |
| hsa-miR-532-3p | 2.41 | Down | 0.01660983 | 0.09316599 |  |
| hsa-miR-1228* | 2.52 | Down | 6.51E-05 | 0.01568949 |  |
| hsa-miR-142-5p | 2.52 | Down | 0.02540104 | 0.11420992 |  |
| hsa-miR-423-5p | 2.59 | Down | 0.00792332 | 0.07506587 |  |
| hsa-miR-494 | 2.61 | Down | 0.0037696 | 0.06310873 |  |
| hsa-miR-942 | 2.65 | Down | 0.02907213 | 0.12142103 |  |
| hsa-miR-595 | 2.66 | Down | 0.00516414 | 0.06856746 |  |
| hsa-miR-3663-5p | 2.77 | Down | 0.01498037 | 0.08980768 |  |
| hsa-miR-181a | 2.78 | Down | 0.01700127 | 0.09316599 |  |
| hsa-miR-206 | 2.80 | Down | 0.00013762 | 0.02228672 |  |
| hsa-miR-320a | 2.87 | Down | 0.02593208 | 0.1157339 |  |
| hsa-miR-564 | 2.88 | Down | 0.0035641 | 0.06310873 |  |
| hsa-miR-548f | 3.06 | Down | 3.24E-05 | 0.01568949 |  |
| hsa-miR-99b | 3.36 | Down | 0.02537365 | 0.11420992 |  |
| hsa-miR-92a | 3.37 | Down | 0.00016646 | 0.02228672 |  |
| hsa-miR-574-5p | 3.58 | Down | 8.79E-05 | 0.01764673 |  |
| hsa-miR-623 | 4.14 | Down | 0.00743978 | 0.07313614 |  |
| hsa-miR-222 | 4.47 | Down | 0.01150077 | 0.08367891 |  |
| hsa-miR-1268 | 4.61 | Down | 0.00175265 | 0.04422652 |  |
| hsa-miR-181d | 6.90 | Down | 0.00104448 | 0.04059976 |  |
| hsa-miR-3653 | 9.35 | Down | 0.00033814 | 0.02463953 |  |

**TOF-all.** all patients with Tetralogy Of Fallot; **TOF-noHF.** TOF patients without Heart Failure; **TOF-HF**= TOF patients with Heart Failure; **AUC.** Area Under the receiver operating characteristic Curve
