# Supplementary figures and images for: Analysis of circulating microRNAs in patients with repaired Tetralogy of Fallot with and without heart failure
Source: J Transl Med. 2017 Jul 10;15:156. doi: 10.1186/s12967-017-1255-z (PMC5504636; doi:10.1186/s12967-017-1255-z)

## Slide 1
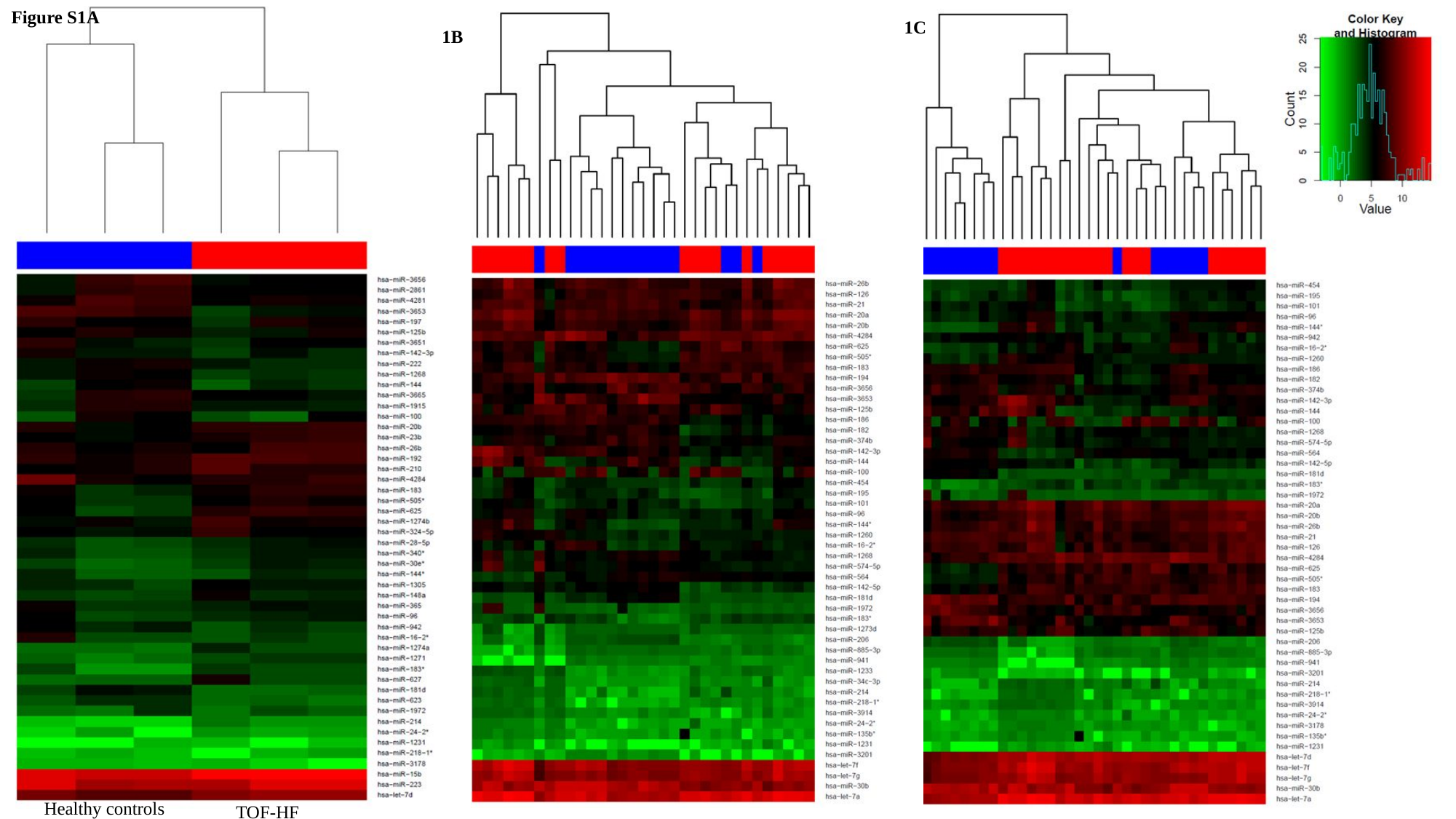

Figure S1A
1C
1B
Healthy controls
TOF-HF

Supplement: Supplementary file 2 — Additional file 2: Figure S1. Unsupervised hierarchical clustering (Euclidian distance, complete linkage) of the patients compared to matched controls based on expression of the 50 with the highest variance. 1A) the first cluster contains mostly controls and the second most of the TOF-HF patients. 1B) and 1C) A more detailed distinction between the TOF-noHF and TOF-all subset, each matched to controls based on the clustering dendrogram were, however, not conclusive. [file 12967_2017_1255_MOESM2_ESM.pptx]
