# Supplementary material for: Analysis of circulating microRNAs in patients with repaired Tetralogy of Fallot with and without heart failure
Source: J Transl Med. 2017 Jul 10;15:156. doi: 10.1186/s12967-017-1255-z (PMC5504636; doi:10.1186/s12967-017-1255-z)
Supplement: Supplementary file 3 — Additional file 3: Figure S2. Target network: for the three validated miRNAs in brown, target genes are presented in blue. [file 12967_2017_1255_MOESM3_ESM.pptx]

## Slide 1
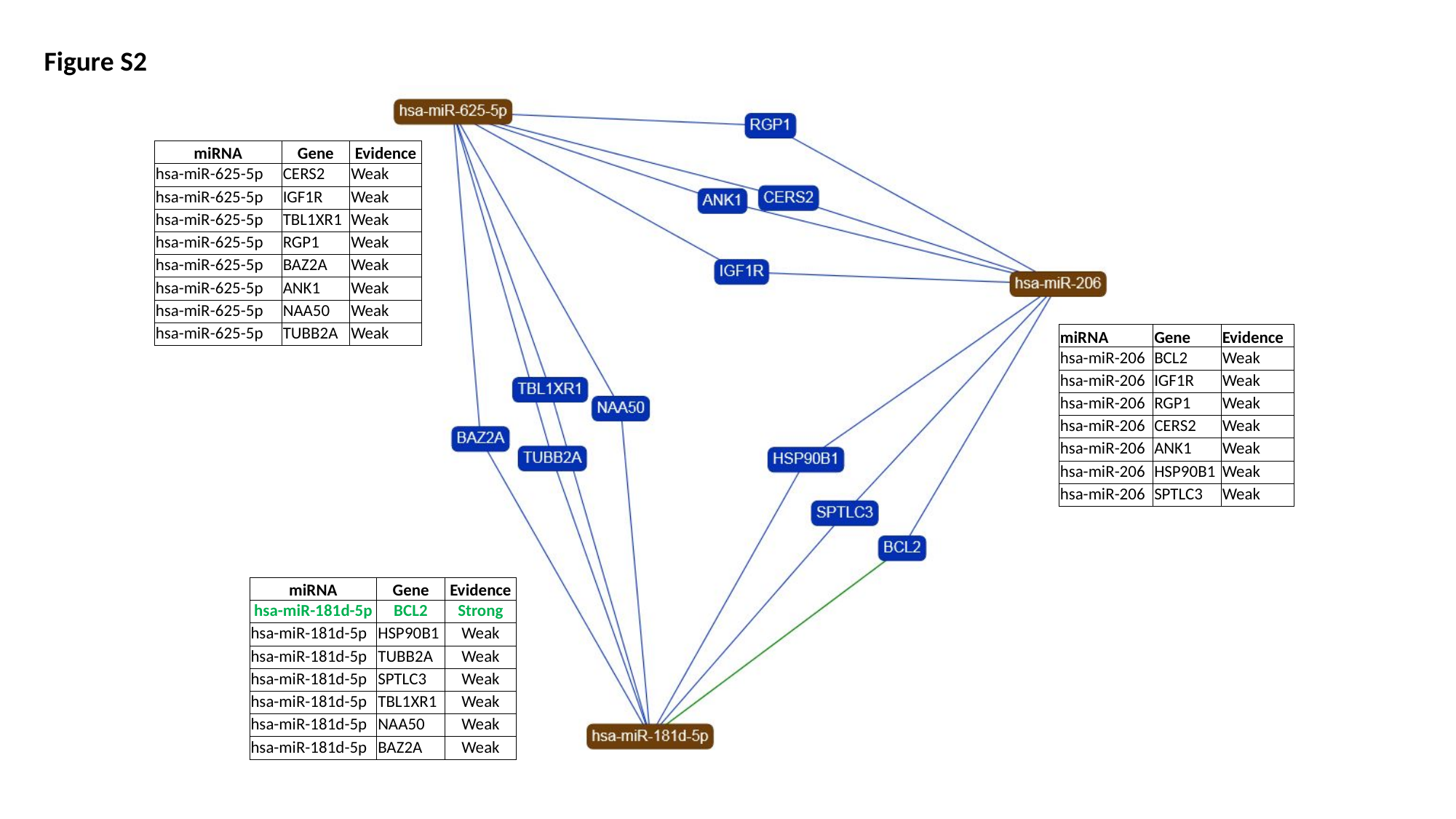

Figure S2
| miRNA | Gene | Evidence |
| --- | --- | --- |
| hsa-miR-625-5p | CERS2 | Weak |
| hsa-miR-625-5p | IGF1R | Weak |
| hsa-miR-625-5p | TBL1XR1 | Weak |
| hsa-miR-625-5p | RGP1 | Weak |
| hsa-miR-625-5p | BAZ2A | Weak |
| hsa-miR-625-5p | ANK1 | Weak |
| hsa-miR-625-5p | NAA50 | Weak |
| hsa-miR-625-5p | TUBB2A | Weak |
| miRNA | Gene | Evidence |
| --- | --- | --- |
| hsa-miR-206 | BCL2 | Weak |
| hsa-miR-206 | IGF1R | Weak |
| hsa-miR-206 | RGP1 | Weak |
| hsa-miR-206 | CERS2 | Weak |
| hsa-miR-206 | ANK1 | Weak |
| hsa-miR-206 | HSP90B1 | Weak |
| hsa-miR-206 | SPTLC3 | Weak |
| miRNA | Gene | Evidence |
| --- | --- | --- |
| hsa-miR-181d-5p | BCL2 | Strong |
| hsa-miR-181d-5p | HSP90B1 | Weak |
| hsa-miR-181d-5p | TUBB2A | Weak |
| hsa-miR-181d-5p | SPTLC3 | Weak |
| hsa-miR-181d-5p | TBL1XR1 | Weak |
| hsa-miR-181d-5p | NAA50 | Weak |
| hsa-miR-181d-5p | BAZ2A | Weak |
